# Molecular, morphological and acoustic assessment of the genus *Ophryophryne* (Anura, Megophryidae) from Langbian Plateau, southern Vietnam, with description of a new species

**DOI:** 10.3897/zookeys.672.10624

**Published:** 2017-05-03

**Authors:** Nikolay A. Poyarkov Jr., Tang Van Duong, Nikolai L. Orlov, Svetlana S. Gogoleva, Anna B. Vassilieva, Luan Thanh Nguyen, Vu Dang Hoang Nguyen, Sang Ngoc Nguyen, Jing Che, Stephen Mahony

**Affiliations:** 1 Department of Vertebrate Zoology, Biological faculty, Lomonosov Moscow State University, Leninskiye Gory, Moscow, GSP-1, 119991, Russia; 2 Joint Russian-Vietnamese Tropical Research and Technological Center, 63 Nguyen Van Huyen Road, Nghia Do, Cau Giay, Hanoi, Vietnam; 3 Vietnam National Museum of Nature, Vietnam Academy of Science and Technology (VAST), 18 Hoang Quoc Viet Road, Hanoi, Vietnam; 4 Zoological Institute, Russian Academy of Sciences, Universitetskaya nab., 1, St. Petersburg, 199034, Russia; 5 A.N. Severtsov Institute of Ecology and Evolution, Russian Academy of Sciences, Leninskii pr., 33, Moscow 119071, Russia; 6 Zoological Museum of the Lomonosov Moscow State University, Bolshaya Nikitskaya st. 6, Moscow 125009, Russia; 7 Institute of Tropical Biology, Vietnam Academy of Science and Technology (VAST), 85 Tran Quoc Toan St., District 3, Ho Chi Minh City, Vietnam; 8 State Key Laboratory of Genetic Resources and Evolution State, Kunming Institute of Zoology, Chinese Academy of Sciences, Kunming 650223, Yunnan, China; 9 Southeast Asia Biodiversity Research Institute, Chinese Academy of Sciences, Yezin, Nay Pyi Taw 05282, Myanmar; 10 UCD School of Biology and Environmental Science, UCD Science Centre (West), University College Dublin, Belfield, Dublin 4, Ireland; 11 Department of Life Sciences, The Natural History Museum, South Kensington, London, United Kingdom

**Keywords:** 12S rRNA, 16S rRNA, advertisement call, amphibian, biodiversity, Da Lat Plateau, frog, Indochina, southeast Asia, taxonomy, Truong Son

## Abstract

Asian Mountain Toads (*Ophryophryne*) are a poorly known genus of mostly small-sized anurans from southeastern China and Indochina. To shed light on the systematics within this group, the most comprehensive mitochondrial DNA phylogeny for the genus to date is presented, and the taxonomy and biogeography of this group is discussed. Complimented with extensive morphological data (including associated statistical analyses), molecular data indicates that the Langbian Plateau, in the southern Annamite Mountains, Vietnam, is one of the diversity centres of this genus where three often sympatric species of *Ophryophryne* are found, *O. gerti*, *O. synoria* and an undescribed species. To help resolve outstanding taxonomic confusion evident in literature (reviewed herein), an expanded redescription of *O. gerti* is provided based on the examination of type material, and the distributions of both *O. gerti* and *O. synoria* are considerably revised based on new locality records. We provide the first descriptions of male mating calls for all three species, permitting a detailed bioacoustics comparison of the species. We describe the new species from highlands of the northern and eastern Langbian Plateau, and distinguish it from its congeners by a combination of morphological, molecular and acoustic characters. The new species represents one of the smallest known members of the genus *Ophryophryne*. At present, the new species is known from montane evergreen forest between 700–2200 m a.s.l. We suggest the species should be considered Data Deficient following IUCN’s Red List categories.

## Introduction

Asian Mountain toads (*Ophryophryne* Boulenger, 1903) are a small group of frogs from southeast Asia with a rather limited distribution mostly in mountains of eastern Indochina and adjacent parts of southern China (Yunnan and Guangxi) and northern Thailand (Inger et al. 1999, Ohler 2003, Orlov and Ananjeva 2007, Yang 2008). The genus *Ophryophryne* is still poorly studied, to date five (Ohler 2003, Stuart et al. 2006) or six (Stuart et al. 2010) species are recognized, with little consensus on the taxonomic status of several forms. All of the known *Ophryophryne* species have been reported from the Truong Son or Annamite mountains in Vietnam, which may be considered as an area of highest diversity for this group (Orlov and Ananjeva 2007).

The systematic status of *Ophryophryne* has long been a source of confusion. Boulenger (1903) described the genus and species *O. microstoma* Boulenger, 1903, and though he clearly stated that *Ophryophryne* is closely allied to *Megophrys* Kuhl & Van Hasselt, 1822, he noted characters also shared by members of the family Bufonidae Gray, 1825 (lacking vomerine and maxillary teeth and presence of horizontal pupil). Subsequently, Noble (1926), mostly based on morphology of the pectoral girdle, clearly demonstrated that the genus *Ophryophryne* is a member of Pelobatidae (at the time including the subfamily Megophryinae Bonaparte, 1850), and assumed its close affinities to *Megophrys*. However, due to widespread misinterpretation of Boulengers’ original statement, the genus *Ophryophryne* was nevertheless incorrectly listed as a member of Bufonidae in several classical works on batrachians (Bourret 1937, 1942, Gorham 1974, Guibé 1950, Nguyen and Ho 1996, Taylor 1962).

The systematic status of the genus *Ophryophryne* among the Megophryidae has been discussed in several works. Liu and Hu (1962) provided the first description of the *Ophryophryne* tadpole which was remarkably similar to those of *Megophrys*, which led Dubois (1980) to rank Ophryophryne at the level of subgenus within Megophrys. Soon afterwards, Dubois re-evaluated his proposition and elevated *Ophryophryne* back to the genus-level status (Dubois 1987). Summarizing available cytological, morphological and ecological evidence, Rao and Yang (1997) proposed to split *Megophrys* *s. lato*, regarding *Ophryophryne* as a separate genus, as well as the former *Megophrys* subgenera, *Megophrys* *s. stricto*, *Atympanophrys* Tian & Hu, 1983, *Brachytarsophrys* Tian & Hu, 1983 and *Xenophrys* Günther, 1864. Several studies indicated close affinities of *Ophryophryne* to the genus *Xenophrys* (Tian and Hu 1983, 1985, Frost 1985, Ye et al. 1993, Rao and Yang 1997, Manthey and Grossmann 1997). Delorme et al. (2006) recognized the tribe Xenophryini, containing two genera *Ophryophryne* and *Xenophrys*, and most recent faunal reviews have treated *Ophryophryne* as a valid genus within Megophryidae (Fei et al. 1999, 2005, 2009, 2010, 2012, Orlov and Ananjeva 2007, Nguyen et al. 2009). Recently, Mahony (2011a) suggested that insufficient evidence was available for the morphological distinction of *Xenophrys* from *Megophrys* and suggested to retain the historical usage of *Megophrys* *s. lato* for species of both genera pending a taxonomic review of the group, however, he did not discussed the status of *Ophryophryne*.

Though a comprehensive phylogeny of the genus *Ophryophryne* is still pending, preliminary molecular data were contradictory, suggesting both as sister-clade relationships of *Ophryophryne* with respect to a monophyletic group composed of *Xenophrys*, *Megophrys*, and *Brachytarsophrys* (Pyron and Wiens 2011), or providing evidence of the paraphyly of *Xenophrys* with respect to *Ophryophryne* (e.g. Wang et al. 2012). A recent phylogenetic study on Megophryinae by Chen et al. (2017) provides new insights on evolutionary relationships within this group, indicating contrasting (though poorly supported) phylogenetic positions of *Ophryophryne* in their multilocus nuclear-gene based phylogeny and matrilineal mtDNA genealogy. Chen et al. (2017) preliminarily recognized *Ophryophryne* as one of the five monophyletic genera within Megophryinae (*Ophryophryne*, *Brachytarsophrys*, *Xenophrys*, *Atympanophrys* and *Megophrys*). However, Mahony et al. (2017) provides an alternative hypothesis based on extensive morphological studies and a larger nuclear gene dataset. They provided compelling evidence (recent diversification, insufficient morphological or biological distinction of major clades) for the consideration of Megophryinae to be treated as a single genus, *Megophrys*, with seven sub-clades (including *Ophryophryne*) being treated as subgenus level taxa. Their phylogenetic analyses provided strong support for the sister taxa relationship of *Ophryophryne* and a clade corresponding to *Panophrys* (previously considered a synonym or subclade of *Xenophrys*, e.g., Delorme et al. 2006, Chen et al. 2017).

For a long time after its’ description, the genus *Ophryophryne* was thought to include a single species, *O. microstoma*, described from “Mau Son” in Tonkin (northern Vietnam). Later, Bourret (1937) described a second species, *O. poilani* Bourret, 1937, based upon a single, badly preserved specimen from “Dong Tam Ve” in Quang Tri Prov. of Annam (central Vietnam). Almost half a century later a third species, *O. pachyproctus* Kou, 1985, was described by Kou (1985) from Mengla County in Yunnan Prov. (southern China). Ohler (2003) revised the available material on the genus and, mostly based on samples collected by M. Smith (in southern Vietnam), and I.S. Darevsky and N.L. Orlov (in central Vietnam), described two more species: *O. gerti* Ohler, 2003 and *O. hansi* Ohler, 2003, respectively (the type series of *O. gerti* included specimens from the Langbian Plateau in southern Vietnam, and Laos). Ohler (2003) revised diagnostic characters and provided a key for the genus; she also examined the type specimen of *O. poilani* and considered it to be a junior synonym of *O. microstoma* (opinion not shared by Stuart et al. 2010). The last major progress on the taxonomy of *Ophryophryne* was made by Stuart et al. (2006), who reported the genus for Cambodia and described one more species, *O. synoria* Stuart, Sok & Neang, 2006 from Mondolkiri Prov. in eastern hilly Cambodia, near the Vietnamese border. A recent review of southern Vietnamese herpetofauna by Vassilieva et al. (2016) based on morphological evidence recorded *O. synoria* for lowland areas of Dong Nai and Binh Phuoc provinces.

The Langbian (or Da Lat) Plateau forms the southernmost edge of the Annamite Mountains, or Truong Son Range, a mountain chain spanning the breadth of Indochina, including parts of Vietnam, Laos and Cambodia. To date, following the review by Ohler (2003), only *Ophryophryne gerti* has been recorded from the high elevations (above 1000 m a.s.l.) of the Langbian Plateau (Stuart et al. 2010, Nguyen et al. 2009, 2014). However, our recent fieldwork in this area from 2007 until 2016 revealed the presence of at least three morphologically distinct species, often recorded in syntopy (Orlov et al. 2008, Poyarkov and Vassilieva 2011). Further investigation of partial 12S rRNA–16S rRNA mtDNA gene sequences, as well as the study of advertisement calls from the Langbian *Ophryophryne* populations, herein confirm their specific status and reveals that one of the lineages represents a previously undescribed species. We also provide the first preliminary mtDNA phylogeny for the genus *Ophryophryne* and discuss the biogeography of the genus in Indochina in light of our new data.

## Materials and methods

**Figure 1.**
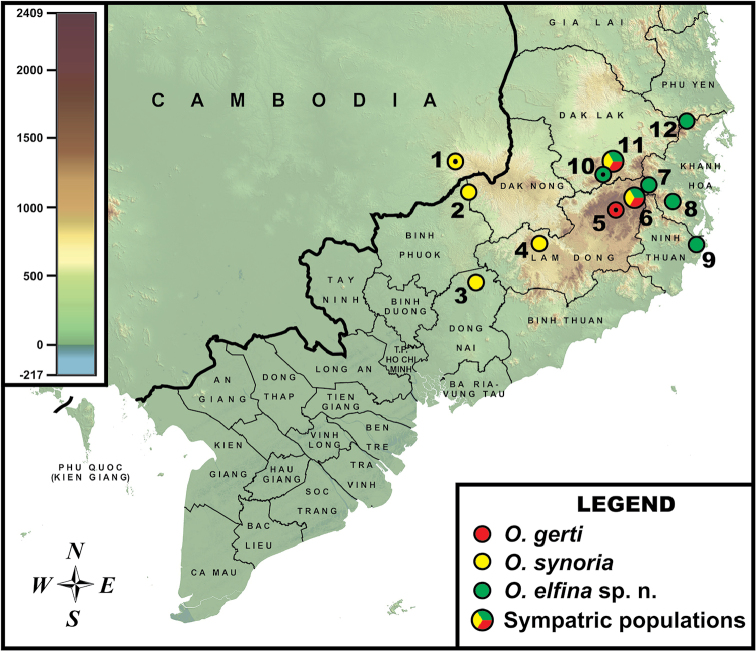
Distribution of *Ophryophryne* species in southern Indochina (Langbian Plateau in Vietnam, and adjacent regions of Camodia), indicating localities sampled in this study. Black dot in the center of an icon indicates the type locality of the new species. Locality information. **1** O Chung Chry stream, Samling Logging Concession, O’Rang Dist., Mondolkiri Prov., Cambodia (500 m a.s.l.) (Stuart et al. 2006; type locality of *O. synoria*; Mahony et al. 2017) **2** Bu Gia Map N.P., Binh Phuoc Prov., Vietnam (400 m a.s.l.) (Vassilieva et al. 2016; this study) **3** Nam Cat Tien sector of Dong Nai Biosphere Reserve, Dong Nai Prov., Vietnam (200 m a.s.l.) (Vassilieva et al. 2016; this study) **4** Loc Bac forest, Bao Loc Dist., Lam Dong Prov., Vietnam (830 m a.s.l.) (this study) **5** Cam Ly River (Ohler 2003: type locality of *O. gerti*) and Nui Ba Mt. in environs of Dalat city, Lam Dong Prov., Vietnam (ca. 1000–1800 m a.s.l.) (this study) **6** Environs of Bidoup Mt. (2000 m a.s.l.), and Giang Ly Ranger Station (1500 m a.s.l.), Bidoup–Nui Ba N.P., Lam Dong Prov., Vietnam (Poyarkov [Paiarkov] and Vassilieva 2011; this study) **7** Hon Giao Mt., Bidoup–Nui Ba N.P., Lam Dong and Khanh Hoa provincial border, Vietnam (1900–2000 m a.s.l.) (this study) **8** Hon Ba Mt., Hon Ba N.R., Dien Khanh Dist., Khanh Hoa Prov., Vietnam (950–1510 m a.s.l.) (Nguyen et al. 2014; this study) **9** Nui Chua Mt., Nui Chua N.P., Ninh Hai Dist., Ninh Thuan Prov., Vietnam (780 m a.s.l.) (this study) **10** Chu Pan Fan Mt., Chu Yang Sin N.P., Lak Dist., Dak Lak Prov., Vietnam (1900 m a.s.l.) (type locality of *Ophryophryne elfina* sp. n.; this study) **11** Chu Yang Sin Mt. environs, Krong Kmar Commune, Krong Bong Dist., Dak Lak Prov., Vietnam (700–2000 m a.s.l.) (Orlov et al. 2008; this study) **12** Tay Hoa Dist., Phu Yen Prov., Vietnam (700 m a.s.l.) (this study).

**Sample collection.** All specimens were collected during fieldwork in southern Vietnam in 2007–2016. Frogs were collected mostly during night excursions by opportunistic visual searching, or by sound when calling. Geographic coordinates were obtained using a Garmin GPSMAP 60CSx GPS receiver and recorded in datum WGS 84. The geographic position of the surveyed localities and the distribution of *Ophryophryne* species in the southern Annamite Mountains (Truong Son) and adjacent regions of southern Indochina (eastern Cambodia) are shown in Fig. 1. The newly collected specimens were deposited in the herpetological collection of the Zoological Museum of Moscow State University in Moscow, Russia (ZMMU) and the Institute of Tropical Biology Zoological Collection, Ho Chi Minh City, Vietnam (ITBCZ). Examined specimens of compared species are stored in herpetological collections of ZMMU, Zoological Institute R.A.S., St. Petersburg, Russia (ZISP), Natural History Museum, London, United Kingdom (NHMUK, formerly BMNH, though the latter acronym is retained for specimen numbers for comparability with older literature), Field Museum of Natural History, Chicago, USA (FMNH), and Yunnan University, Faculty of Biology, Kunming, China (YU).

**Morphology.** Specimens were photographed in life, and tissue samples for genetic analyses were taken prior to preservation, and stored in 96% ethanol. We recorded morphological data from specimens fixed and stored in 75% ethanol.

Measurements to the nearest 0.1 mm were taken using either a digital caliper, or a dissecting microscope; morphometrics of adult frogs and character terminology follows Mahony (2011a) and Mahony et al. (2013). Morphometric abbreviations are as follows:

**SVL** snout to vent length;

**HW** head width;

**HL** head length;

**ED** eye diameter;

**TYD** tympanum diameter;

**TYE** eye to tympanum distance;

**SL** snout length;

**EN** eye to narial distance;

**SN** narial to snout distance;

**IUE** interorbital distance, between upper eyelids;

**IN** internarial distance;

**UEW** upper eyelid width;

**FAL** forearm length;

**HAL** hand length;

**FIL** first finger length;

**FIIL** second finger length;

**FIIIL** third finger length;

**FIVL** fourth finger length;

**SHL** shank length;

**TL** thigh length;

**FOL** foot length;

**TFOL** tibiotarsal articulation to tip of fourth toe distance;

**IMT** inner metatarsal tubercle length.

Additionally, for the description of the type series we measured the distance between anterior orbital borders (**IFE**); distance between posterior orbital borders (**IBE**); first toe length (**TIL**); second toe length (**TIIL**); third toe length (**TIIIL**); fourth toe length (**TIVL**); fifth toe length (**TVL**). All measurements were taken on the right side of the specimen, except when a character was damaged, in which case the measurement was taken on the left side. Entire skin surface of all specimens were examined by microscope for the presence of dermal microstructures. Sex was determined by direct observation of calling in life and/or gonadal inspection by dissection.

Morphological description of larval stages included the following 15 measurements: total length (**TOL**); body length (**BL**); tail length (**TAL**); maximum body width (**BW**); maximum body height (**BH**); maximum tail height (**TH**); snout to vent length (**SVL**); snout to spiracle distance (**SSp**); maximum upper tail fin height (**UF**); maximum lower tail fin height (**LF**); internarial distance (**IN**); interpupilar distance (**IP**); rostro-narial distance (**RN**); naro-pupilar distance (**NP**); eye diameter (**ED**). The oral disk width and the labial tooth row formula were not recorded since in *Ophryophryne* the oral disk is modified to an extensive funnel which is closed when fixed in preservative, and oral disk structures typical for most other anurans are absent. Tadpoles were staged after Gosner (1960); morphometrics followed Grosjean (2001, 2003) and Poyarkov et al. (2015b).

All statistical analyses were performed with Statistica 6.0 (StatSoft, Inc. 2001). Morphometric characters were used for univariate analyses, corrected by body size. Sexes were separated for subsequent comparisons among the samples. One-way ANOVA and Duncan’s post hoc test were used for morphometric comparisons. Multivariate statistical analyses were conducted for examination of overall morphological variation among studied populations. If some characters showed high correlation between each other, all but one of them were omitted in order to exclude the overweighting effect of these characters on the analyses. After metric values were log e-transformed, a principal component analysis (PCA) was conducted. The additional specimens of the undescribed *Ophryophryne* species, measured by LTN, were not included in the PCA to avoid potential error due to inter-observer variation of measurement techniques. A significance level of 95% was used in all statistical tests.

Comparative morphological data were obtained from museum specimens of *Ophryophryne* and (when available) photographs of these specimens in life (see Appendix 1). Data on morphology and taxonomy of *Ophryophryne* are also available from the following literature: *O. microstoma* (Bourret 1942, Liu and Hu 1962, Yang 1991, Ye et al. 1993, Fei et al. 1999, 2009, 2010, 2012, Zhang and Wen 2000, Ohler 2003, Bain et al. 2007, Yang 2008), *O. pachyproctus* (Kou 1985, Yang 1991, Ye et al. 1993, Fei et al. 1999, 2009, 2010, 2012), *O. poilani* (Bourret 1937, 1942, Ohler 2003). However, due to the considerable undiagnosed diversity within Megophryidae (Chen et al. 2017, Mahony et al. 2017), where available, we relied on the examination of type specimens, topotypic material and/or original species descriptions. Only characters verified on all specimens in the type series and referred specimens are used to represent the new species in the comparison and diagnosis sections. Specimens of O. cf. poilani listed in Appendix 1 were not used in the comparison of the undescribed species with *O. poilani*.

**DNA isolation and sequencing.** For molecular analysis, total genomic DNA was extracted from ethanol-preserved muscle or liver tissues using either standard phenol-chloroform extraction procedures (Hillis et al. 1996) followed with isopropanol precipitation (at Moscow State University: hereafter MSU), or a Qiagen DNeasy® Blood & Tissue Kit primarily following manufacturers’ instructions, with the exception of an extended (10 minute) soaking step prior to the elution of extracted DNA from the column, and additional final elution step using 40 μl H_2_O (at University College Dublin: hereafter UCD). The isolated total genomic DNA was visualized using agarose gel electrophoresis in the presence of ethidium bromide (MSU), or SafeView^TM^ (Applied Biological Materials Inc. — at UCD). The concentration of total DNA was measured using NanoDrop 2000 (MSU) or NanoDrop 1000 (Thermo Scientific) (UCD), and consequently either adjusted to ca. 100 ng DNA/μl (MSU), or 10 ng DNA/μl (UCD).

We amplified sequences of a continuous fragment including partial sequences of 12S rRNA and 16S rRNA genes and complete t-val gene sequence, to obtain a fragment of up to 2077 bp (base pairs) of mtDNA. For some adult specimens and larvae a partial ca. 460–500 bp fragment of the 16S rRNA gene was sequenced for molecular identification purposes. 16S rRNA is a molecular marker widely applied for biodiversity surveys in amphibians (Vences et al. 2005a, 2005b, Vieites et al. 2009), and has proven to be particularly useful in studies of megophryid diversity (Matsui et al. 2010, 2014, Ohler et al. 2011, Stuart et al. 2011, 2012, Hamidy et al. 2012, Rowley et al. 2010a, 2011a, 2012, Jiang et al. 2013, Poyarkov et al. 2015a and references therein). Amplification was performed in 25 μl reactions using either ca. 50 ng genomic DNA, 10 nmol of each primer, 15 nmol of each dNTP, 50 nmol additional MgCl_2_, Taq PCR buffer (10 mM Tris-HCl, pH 8.3, 50 mM KCl, 1.1 mM MgCl_2_ and 0.01% gelatine) and 1 U of Taq DNA polymerase (MSU), or 2.0 μl of genomic DNA (10 ng/μl), 2.5 μl Sigma 10x PCR buffer (excluding MgCL_2_), 0.5 μl MgCL_2_, 0.5 μl dNTP mix, 0.5 μl forward and reverse primer (10 ng/μl), 0.2 μl Platinum® *Taq* DNA Polymerase (Invitrogen) and 18.3 μl PCR grade H_2_O (UCD). Primers used in PCR and sequencing were as follows: forward primers: 12SAL (AAACTGGGATTAGATACCCCACTAT; Zhang et al. 2008), LX12SN1 (TACACACCGCCCGTCA; Zhang et al. 2008), 12SA (CTGGGATTAGATACCCCACTA; Palumbi 1996), L1879 (CGTACCTTTTGCATCATGGTC; Matsui et al. 2010), L2188 (AAAGTGGGCCTAAAAGCAGCCA; Matsui et al. 2006), 16L-1 (CTGACCGTGCAAAGGTAGCGTAATCACT; Hedges 1994); reverse primers: 16S2000H (GTGATTAYGCTACCTTTGCACGGT; Zhang et al. 2008), LX16S1R (GACCTGGATTACTCCGGTCTGAACTC; Zhang et al. 2008), 16SBr (CCGGTCTGAACT-CAGATCACGT; Palumbi 1996), H1923 (AAGTAGCTCGCTTAGTTTCGG; Matsui et al. 2010), H2317 (TTCTTGTTACTAGTTCTAGCAT; Shimada et al. 2011), Will6 (CCCTCGTGATGCCGTTGATAC; Wilkinson et al. 2002). Tadpoles were assigned to species based on short 16S rRNA sequences obtained using the primer pair 16L-1 (see above) and 16H-1 (CTCCGGTCTGAACTCAGATCACGTAGG; Hedges 1994). Two Touch-Down (TD) PCR reaction protocols (Murphy and O’Brien 2007) were used: TD 63–57 for 12SA and 16SBr primers and TD 55 for all other primer pairs. Slight differences in reaction protocol were used between MSU, and UCD reactions (in parentheses). TD 55 included an initial denaturation step of 5 (2) min. at 94°C and followed with 10 cycles of denaturation for 30 (45) sec. at 96°C, primer annealing for 30 (40) sec. with annealing temperature decreasing by 1°C per cycle from 65°C to 55°C and extension step for 1 min. at 72°C, followed with 35 cycles of 30 (45) sec. at 96°C, 30 (40) sec. at 55°C and 4 (1) min. at 60°C (72°C), with the final extension step for 10 min. at 72°C. TD 63–57 consisted of 2 min. at 95°C, 6 cycles of 45 sec. at 95°C, 40 sec. at 63°C with a reduction of 1°C each cycle, 1 min. at 72°C, followed by 35 cycles of 45 sec. at 95°C, 40 sec. at 57°C and 1 min at 72°C, and a final step of 10 min. at 72°C. PCR products were loaded onto 1% agarose gels, stained with either GelStar gel stain (Cambrex: at MSU) or SafeView^TM^ (at UCD), and visualized in a Dark reader transilluminator (Clare Chemical). If distinct bands were produced, PCR products were purified either using 2 μl, from a 1:4 dilution of ExoSapIt (Amersham), per 5 μl of PCR product prior to cycle sequencing (MSU), or using TSAP (Promega) following manufacturers’ instructions (UCD). At MSU, a 10 μl sequencing reaction included 2 μL of template, 2.5 μl of sequencing buffer, 0.8 μl of 10 pmol primer, 0.4 μl of BigDye Terminator version 3.1 Sequencing Standard (Applied Biosystems) and 4.2 μl of water. The cycle-sequencing reaction was 35 cycles of 10 sec. at 96°C, 10 sec. at 50°C and 4 min. at 60°C. Cycle sequencing products were purified by ethanol precipitation. Sequence data collection and visualization were performed on an ABI 3730xl automated sequencer (Applied Biosystems). At UCD, purified PCR products were Sanger sequenced in both directions by Macrogen (Europe). The forward and reverse sequences were checked visually either in Chromas Pro software (Technelysium Pty Ltd., Tewntin, Australia: at MSU) and a consensus sequence was compiled with BioEdit 5.0.9 (Hall 1999: at MSU), or using CodonCodeAligner 3.7.1 (CodonCode Corporation, Dedham, Massachusetts: at UCD). Sequences were submitted to a BLAST search in GenBank to confirm that the intended sequences had been amplified. The obtained sequences are deposited in GenBank under the accession numbers KY425352–KY425411 and KY515232–KY515233 (see Table 1).

**Table 1.** Specimens and GenBank sequences of *Ophryophryne* and outgroup Megophryidae representatives used in molecular analyses. AN – Accession number. Numbers of localities (No. 1–12) correspond to those in Figures 1 and 2. For detailed specimen information see Appendix 1. Asterisk marks holotype specimen, double asterisk marks topotype specimens (continues on next two pages). Museum abbreviations for the specimens from which sequences were generated in this study are explained in the Materials and methods section.

GenBank AN	Voucher ID	Species	Locality	Elevation (m a.s.l.)	Reference
KY022198	FMNH 262778*	*O. synoria*	**1** – Cambodia, Mondolkiri Prov., O’Reang	500	Mahony et al. 2017
KY425353	ZMMU ABV-00379	*O. synoria*	**2** – Vietnam, Binh Phuoc Prov., Bu Gia Map N.P., Dac Ca River	400	this paper
KY425354	ZMMU NAP-00731	*O. synoria*	**2** – Vietnam, Binh Phuoc Prov., Bu Gia Map N.P., Dac Ca River	400	this paper
KY425355	ZMMU ABV-00380	*O. synoria*	**2** – Vietnam, Binh Phuoc Prov., Bu Gia Map N.P., Dac Ca River	400	this paper
KY425356	ZMMU ABV-00376	*O. synoria*	**2** – Vietnam, Binh Phuoc Prov., Bu Gia Map N.P., Dac Ca River	400	this paper
KY425357	ZMMU NAP-00834	*O. synoria*	**3** – Vietnam, Dong Nai Prov., Nam Cat Tien N.P., Da Ta Po River	200	this paper
KY425358	ZMMU ABV-00209	*O. synoria*	**4** – Vietnam, Lam Dong Prov., Bao Loc, Loc Bac forestry	830	this paper
KY425359	ZMMU ABV-00159	*O. synoria*	**4** – Vietnam, Lam Dong Prov., Bao Loc, Loc Bac forestry	830	this paper
KY425360	ZMMU NAP-01756	*O. synoria*	**6** – Vietnam, Lam Dong Prov., Bidoup–Nui Ba N.P., Giang Ly	1500	this paper
KY425361	ZMMU NAP-01787	*O. synoria*	**6** – Vietnam, Lam Dong Prov., Bidoup–Nui Ba N.P., Giang Ly	1500	this paper
KY425362	ZMMU NAP-01835	*O. synoria*	**6** – Vietnam, Lam Dong Prov., Bidoup–Nui Ba N.P., Giang Ly	1500	this paper
KY425363	ZISP NLO-36349	*O. synoria*	**11** – Vietnam, Dak Lak Prov., Chu Yang Sin N.P., Chu Yang Sin Mt.	1000	this paper
KY425364	ZISP NLO-36554	*O. synoria*	**11** – Vietnam, Dak Lak Prov., Chu Yang Sin N.P., Chu Yang Sin Mt.	1000	this paper
KY425365	KIZ-013663**	*O. gerti*	**5** – Vietnam, Lam Dong Prov., Bidoup–Nui Ba N.P., Langbian Mt.	1800	this paper
KY425366	KIZ-013664**	*O. gerti*	**5** – Vietnam, Lam Dong Prov., Bidoup–Nui Ba N.P., Langbian Mt.	1800	this paper
KY425367	KIZ-013662**	*O. gerti*	**5** – Vietnam, Lam Dong Prov., Bidoup–Nui Ba N.P., Langbian Mt.	1800	this paper
KY425368	ZMMU NAP-01878	*O. gerti*	**6** – Vietnam, Lam Dong Prov., Bidoup–Nui Ba N.P., Giang Ly	1500	this paper
KY425369	ZMMU NAP-01789	*O. gerti*	**6** – Vietnam, Lam Dong Prov., Bidoup–Nui Ba N.P., Giang Ly	1500	this paper
KY425370	ZMMU NAP-02471	*O. gerti*	**6** – Vietnam, Lam Dong Prov., Bidoup–Nui Ba N.P., Giang Ly	1500	this paper
KY425371	ZMMU NAP-01790	*O. gerti*	**6** – Vietnam, Lam Dong Prov., Bidoup–Nui Ba N.P., Giang Ly	1500	this paper
KY425372	ZMMU NAP-01788	*O. gerti*	**6** – Vietnam, Lam Dong Prov., Bidoup–Nui Ba N.P., Giang Ly	1500	this paper
KY425373	ZMMU NAP-02758	*O. gerti*	**11** – Vietnam, Dak Lak Prov., Chu Yang Sin N.P.	1000	this paper
KY425374	ZMMU ABV-00530	*O. gerti*	**11** – Vietnam, Dak Lak Prov., Chu Yang Sin N.P.	1000	this paper
KY425375	ZMMU NAP-02759	*O. gerti*	**11** – Vietnam, Dak Lak Prov., Chu Yang Sin N.P.	1000	this paper
KY425376	ZMMU ABV-00577	*O. gerti*	**11** – Vietnam, Dak Lak Prov., Chu Yang Sin N.P.	1000	this paper
KY425377	ZMMU NAP-02760	*O. gerti*	**11** – Vietnam, Dak Lak Prov., Chu Yang Sin N.P.	1000	this paper
KY425378	ZISP NLO-36510	*O. gerti*	**11** – Vietnam, Dak Lak Prov., Chu Yang Sin N.P.	1000	this paper
KY425379	ZMMU ABV-00454	*O. elfina* sp. n.	**6** – Vietnam, Lam Dong Prov., Bidoup–Nui Ba N.P., Bidoup Mt.	2000	this paper
KY425380	ZMMU ABV-00455	*O. elfina* sp. n.	**6** – Vietnam, Lam Dong Prov., Bidoup–Nui Ba N.P., Bidoup Mt.	2000	this paper
KY515233	ZMMU NAP-01169	*O. elfina* sp. n. (larva)	**6** – Vietnam, Lam Dong Prov., Bidoup–Nui Ba N.P., Bidoup Mt.	2000	this paper
KY425381	ZMMU NAP-01782	*O. elfina* sp. n.	**7** – Vietnam, Lam Dong Prov., Bidoup–Nui Ba N.P., Hon Giao Mt.	2000	this paper
KY425382	ZMMU NAP-01783	*O. elfina* sp. n.	**7** – Vietnam, Lam Dong Prov., Bidoup–Nui Ba N.P., Hon Giao Mt.	2000	this paper
KY425383	ZMMU NAP-01757	*O. elfina* sp. n.	**7** – Vietnam, Lam Dong Prov., Bidoup–Nui Ba N.P., Hon Giao Mt.	2000	this paper
KY425384	ZMMU NAP-01758	*O. elfina* sp. n.	**7** – Vietnam, Lam Dong Prov., Bidoup–Nui Ba N.P., Hon Giao Mt.	2000	this paper
KY425385	ZMMU ABV-00316	*O. elfina* sp. n.	**8** – Vietnam, Khanh Hoa Prov., Hon Ba N.R., Hon Ba Mt.	1500	this paper
KY425386	KIZ YPX-05429	*O. elfina* sp. n.	**9** – Vietnam, Ninh Thuan Prov., Nui Chua N.P.	780	this paper
KY425387	KIZ YPX-05457	*O. elfina* sp. n.	**9** – Vietnam, Ninh Thuan Prov., Nui Chua N.P.	780	this paper
KY425388	KIZ YPX-05428	*O. elfina* sp. n.	**9** – Vietnam, Ninh Thuan Prov., Nui Chua N.P.	780	this paper
KY425389	ZMMU NAP-02658*	*O. elfina* sp. n.	**10** – Vietnam, Dak Lak Prov., Chu Yang Sin N.P., Chu Pan Fan Mt.	1900	this paper
KY515232	ZMMU NAP-02673**	*O. elfina* sp. n. (larva)	**10** – Vietnam, Dak Lak Prov., Chu Yang Sin N.P., Chu Pan Fan Mt.	1900	this paper
KY425390	ZISP NLO-36522	*O. elfina* sp. n.	**11** – Vietnam, Dak Lak Prov., Chu Yang Sin N.P., Chu Yang Sin Mt.	2000	this paper
KY425391	ZMMU ABV-00581	*O. elfina* sp. n.	**11** – Vietnam, Dak Lak Prov., Chu Yang Sin N.P., Chu Yang Sin Mt.	1800	this paper
KY425392	ZMMU ABV-00580	*O. elfina* sp. n.	**11** – Vietnam, Dak Lak Prov., Chu Yang Sin N.P., Chu Yang Sin Mt.	1975	this paper
KY425393	DVT-00393	*O. elfina* sp. n.	**12** – Vietnam, Phu Yen Prov., Tay Hoa	700	this paper
KY022203	AMNH A163680	*O. hansi*	Vietnam, Quang Nam Prov., Tra My, Tra Don	930	Mahony et al. 2017
DQ283377	AMNH A163669	*O. hansi*	Vietnam, Quang Nam Prov., Tra My, Tra Don	970	Frost et al. 2006
KY425395	ZMMU NAP-06485	*O. hansi*	Vietnam, Kon Tum Prov., Thac Nham forest	1100	this paper
KY425396	ZMMU NAP-06501	*O. hansi*	Vietnam, Gia Lai Prov., Kon Chu Rang N.R.	1000	this paper
KY425397	ZMMU NAP-06524	*O. hansi*	Vietnam, Gia Lai Prov., Kon Chu Rang N.R.	1000	this paper
KY425398	ZMMU NAP-06502	*O. hansi*	Vietnam, Gia Lai Prov., Kon Chu Rang N.R.	1000	this paper
KY425399	ZMMU NAP-06525	*O. hansi*	Vietnam, Gia Lai Prov., Kon Chu Rang N.R.	1000	this paper
KY022200	KUH 311601	*O. microstoma*	China, Guangxi Prov., Shiwan Dashang N.R., Fulong	500	Mahony et al. 2017
KY022199	AMNH A168682	*O. microstoma*	Vietnam, Lao Cai Prov., Van Ban Dist., Nam Tha	330	Mahony et al. 2017
KY022201	AMNH A163668	O. cf. poilani	Vietnam, Quang Nam Prov., Tra My, Tra Don	980	Mahony et al. 2017
KY022202	AMNH A169287	O. cf. poilani	Vietnam, Thua Thien–Hue Prov., A Luoi Dist., A Roang	680	Mahony et al. 2017
JX564854	ZP-AM 44	*Brachytarsophrys carinense*	–	–	Zhang et al. 2013
KY425404	ZMMU NAP-06324	*Brachytarsophrys feae*	Vietnam, Vinh Phuc Prov., Tam Dao	–	this paper
KY425405	ZMMU NAP-03994	Xenophrys cf. aceras	Thailand, Satun Prov.	–	this paper
KY425406	ZMMU NAP-05005	Xenophrys cf. daweimontis	Vietnam, Dien Bien Prov., Muong Nhe, Muong Nhe N.R.	–	this paper
KY425407	ZMMU NAP-04137	Xenophrys cf. parva	Thailand, Suratthani Prov.	–	this paper
KY425408	ZMMU NAP-04423	Xenophrys cf. maosonensis	Vietnam, Dien Bien Prov., Muong Nhe, Muong Nhe N.R.	–	this paper
AY561308	ZYC1500	*Xenophrys minor*	China	–	Zheng et al. 2004
AY561307	ZYC1513	*Xenophrys omeimontis*	China, Sichuan	–	Zheng et al. 2004
KY425409	DVT-04135	*Xenophrys* sp.	Vietnam, Lao Cai Prov.	–	this paper
KY425410	ZMMU NAP-05095	*Megophrys nasuta*	Malaysia, Sarawak	–	this paper
KJ630505	SCUM120630	*Leptobrachium boringii*	China, Sichuan Prov., Emei Shan Mt.	–	Xu et al. 2014
KY425411	DVT-00298	*Leptobrachium banae*	Vietnam, Phu Yen Prov., Tay Hoa	–	this paper
JX564874	MVZ-Herp-223642	Leptolalax cf. pelodytoides	–	–	Zhang et al. 2013

**Phylogenetic analyses.** Sequences coding for the 12S rRNA–16S rRNA mtDNA genes of 66 megophryid specimens: 53 *Ophryophryne*, representing all currently recognized species, and outgroup sequences of two *Brachytarsophrys* species, eight *Megophrys* *s. lato* species (including seven *Xenophrys* and one *Megophrys* *s. stricto* species), two *Leptobrachium* Tschudi, 1838, and one *Leptolalax* Dubois, 1980 species (Table 1), were included in the final alignment and subjected to phylogenetic analyses. Nucleotide sequences were initially aligned using ClustalX 1.81 (Thompson et al. 1997) with default parameters, and then optimized manually in BioEdit 7.0.5.2 (Hall 1999) and MEGA 6.0 (Tamura et al. 2013). Mean uncorrected genetic distances (*p*-distances) between sequences were determined with MEGA 6.0 (Tamura et al. 2013); the existence of “barcode gap” was estimated using the online version of ABGD (Puillandre et al. 2012). MODELTEST v.3.06 (Posada and Crandall 1998) was used to estimate the optimal evolutionary models to be used for the data set analysis. The best-fitting model as suggested by the Akaike Information Criterion (AIC) was the general time-reversible (GTR) model of DNA evolution with a gamma shape parameter (G).

Maximum Likelihood (ML) analysis was conducted using Treefinder (Jobb et al. 2004). Transitions and transversions were equally weighted, and gaps were treated as missing data. Confidence in tree topology was tested by non-parametric bootstrap analysis (Felsenstein 1985) with 1000 replicates. Bayesian inference (BI) was conducted using MrBayes 3.1.2 (Huelsenbeck and Ronquist 2001, Ronquist and Huelsenbeck 2003); Metropolis-coupled Markov chain Monte Carlo (MCMCMC) analyses were run with one cold chain and three heated chains for four million generations and sampled every 1,000 generations. Five independent MCMCMC runs were performed and 1,000 trees were discarded as burn-in. Confidence in tree topology was assessed by posterior probability (Huelsenbeck and Ronquist 2001).

We *a priori* regarded tree nodes with bootstrap (BS) values 75% or greater and posterior probabilities (BPP) values over 0.95 as sufficiently resolved, BS values between 75% and 50% (BPP between 0.95 and 0.90) were regarded as tendencies, and BS values below 50% (BPP below 0.90) were considered to be unresolved (Huelsenbeck and Hillis 1993).

**Acoustic analyses.** Calls were recorded using a portable digital audio recorder Zoom h4n (ZOOM Corporation, Tokyo, Japan) in stereo mode with 96 kHz sampling frequency and 16-bit precision, or using a Marantz 660 digital tape recorder (D&M Professional, Kanagawa, Japan) in mono mode with sampling rate at 48 kHz and 16-bit precision with a high-sensitivity Sennheiser K6 ME66 cardioid electret condenser microphone (Sennheiser electronic, Wedemark, Germany), or using a Nikon D 600 digital SLR camera (Nikon Corporation, Japan) in video mode with audio tracks removed from video recordings using Avisoft SASLab Pro software v. 5.2.05 (Avisoft Bioacoustics, Germany) with a 48 kHz sampling frequency and 16-bit precision. Temperature was measured at the calling sites immediately after audio recording using a digital thermometer, KTJ TA218A Digital LCD Thermometer-Hydrometer. All recordings were made in situ in the natural habitats of respective specimens. Advertisement calls of the undescribed *Ophryophryne* species were recorded on the mountain summit of Bidoup in the Bidoup-Nui Ba National Park (hereafter N.P.), Lam Dong Prov., eastern edge of the Langbian Plateau, Vietnam (12°06'42.4"N; 108°39'33.6"E, 1930–1940 m a.s.l.), on 10 and 15 April 2014, and 10 February 2015, between 16:05–18:35 h and at temperatures from 11.3°C in February to 17.5°C in April. In total, we made five recordings from three vocalizing males. Advertisement calls of *O. gerti* were recorded in Chu Yang Sin N.P., Dak Lak Prov., northern edge of the Langbian Plateau, Vietnam (12°24'01.6"N; 108°21'11.0"E, 1020 m a.s.l. and 12°25'25.7"N; 108°21'52.5"E, 1040–1045 m a.s.l.), on 22–27 May 2014, between 20:40–23:10 h at 22–22.5°C. In total, we made three recordings from three males. Advertisement calls of *O. synoria* were recorded in Chu Yang Sin N.P., Dak Lak Prov., Tay Nguyen region, Vietnam (12°28'0.94"N; 108°20'45.4"E, 700–800 m a.s.l.) on 25 May 2008, between 21:56–22:30 h at 21°C. In total, three recordings from three males were made.

All recordings were standardized by Avisoft SASLab Pro software v. 5.2.05 in mono format with sampling rate at 48 kHz and 16-bit precision, and low-frequency noise was reduced using the low-pass filter (up to 1000 Hz). Calls were analyzed using Avisoft SASLab Pro software v. 5.2.05; all parameters were measured using the reticule and standard cursors in the spectrogram window of Avisoft. Spectrograms for analyses were created using the Hamming window, FFT-length 1024 points, frame 100%, and overlap 87.5%. Figure spectrograms were created using the Hamming window, FFT-length 512 points, frame 100%, and overlap 75%. In total, we measured 1797 calls of the new *Ophryophryne* sp., 533 calls of *O. gerti* and 200 calls of *O. synoria*.

Four temporal parameters were measured: the duration of each call, the interval between successive calls within each series, the duration of series, the interval between successive series, and five frequency parameters: the initial and final fundamental frequency, the minimum and maximum of fundamental frequency and the frequency of maximum amplitude (also “F peak”). Then we calculated the frequency range as the difference between the maximum and minimum of fundamental frequencies and the call repetition rate per recording/series (calls/s) for each recording/series as a ratio of number of all calls within the recording/series (excluding series consisting of just one call) to recording/series duration. All numerical parameters are given as mean ± SE, the minimum and maximum values are given in parentheses (min–max).

To compare acoustic characteristics between three species of *Ophryophryne* we applied one-way ANOVA with Tukey post hoc for the values of the parameters for which distributions did not differ from normality (p > 0.05, Kolmogorov–Smirnov test). Otherwise we used nonparametric Kruskal-Wallis ANOVA with Mann-Whitney U post hoc test.

The records of advertisement calls were deposited at the Fonoteca Zoologica and are available at the website http://www.fonozoo.com (under the accession numbers 9954–9964).

## Results

### Sequence data

The final alignment of the studied 12S rRNA–16S rRNA mtDNA gene fragment consisted of 2077 sites: 1439 sites were conserved and 567 sites were variable, of which 465 were found to be parsimony-informative. The transition–transversion bias (R) was estimated as 2.06. Nucleotide frequencies were A = 32.8%, T = 27.6%, C = 21.6%, and G = 17.9% (all data given for ingroup only).

### Phylogenetic relationships and geographic distribution of mtDNA haplotypes

**Figure 2.**
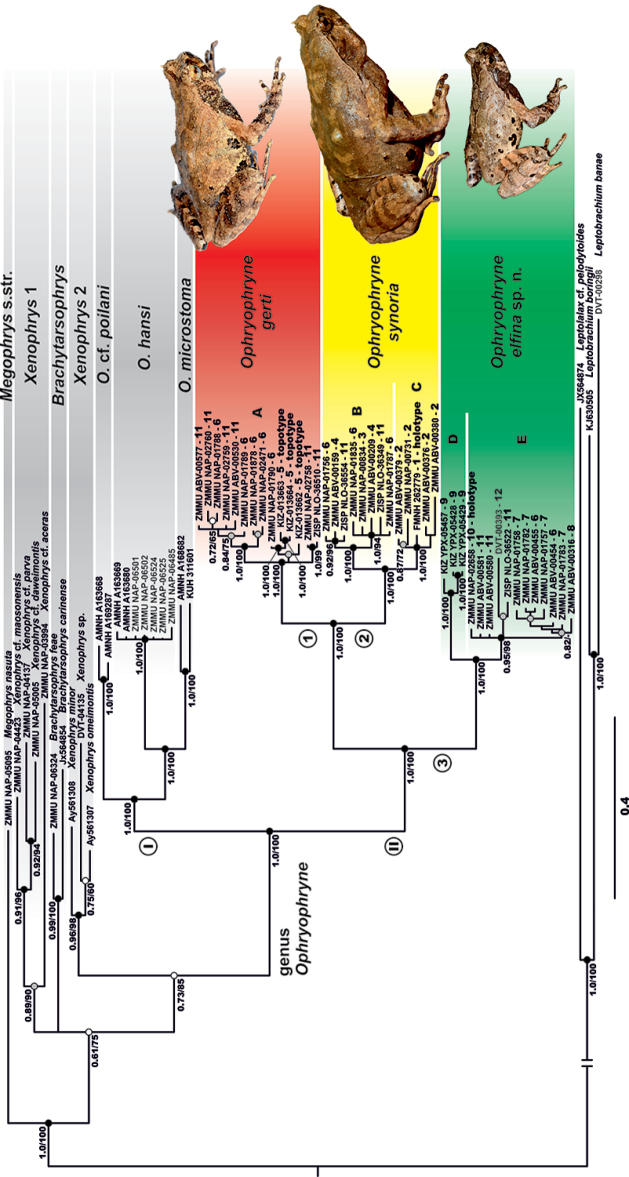
Bayesian inference dendrogram of *Ophryophryne* (including other megophryid outgroups) derived from the analysis of a 2077 bp DNA sequences of the 12S rRNA–16S rRNA mtDNA gene. Voucher specimen IDs and GenBank accession numbers are given in Table 1. Numbers near branches represent BPP/BS support values for BI/ML inferences respectively. Color of clade labels for *Ophryophryne* corresponds to icon colors on Fig. 1. **I** and **II** represent Group I and Group II defined in the results section; numbers **1**, **2** and **3** next to nodes in Group II represent species level clades; **A–E** represent subclades within species level clades.

We achieved high resolution of phylogenetic relationships among taxa within *Ophryophryne*, with all major nodes fully resolved (BPP = 1.0; BS = 100%: Fig. 2). Monophyly of species-level groups and species complexes in *Ophryophryne* were also significantly supported (BS > 90%; BPP ≥ 0.95). However, phylogenetic relationships between the taxa of outgroup Megophryinae are poorly resolved with major nodes in the tree having low or insignificant levels of support (BPP < 0.95; BS < 75%). Bayesian and Maximum Likelihood analyses resulted in essentially similar topologies (see Fig. 2) slightly differing from each other only in associations for several poorly supported outgroup nodes.

Our analyses (Fig. 2) inferred the following set of phylogenetic relationships among studied megophryid taxa:

Our data confirm the monopyly of Megophryinae with respect to outgroup taxa (*Leptobrachium* and *Leptolalax*) (1.0/100; hereafter node support values are given for BPP/BS respectively). Within Megophryinae, the sample of *Megophrys nasuta* (Schlegel, 1858), representing the genus *Megophrys* *s. stricto*, forms the most basal split; this lineage is recovered as a sister group with respect to all other Megophryinae. Phylogenetic relationships among other genera of Megophryinae remain essentially unresolved; while monophyly of *Brachytarsophrys* received high support (0.99/100), species assigned to *Xenophrys* form two weakly supported groups, paraphyletic with respect to *Ophryophryne* and *Brachytarsophrys*.

Monophyly of the genus *Ophryophryne* is strongly supported by all analyses (1.0/100). General topology of the Bayesian tree suggests that the genus *Ophryophryne* is divided into two major groups: the first group joins taxa from southern China, northern and central Indochina (Group I, see Fig. 2), while the second group comprises lineages of *Ophryophryne* confined to the Langbian Plateau in southern Vietnam and adjacent Cambodia (Group II, see Fig. 2). Both clades are reciprocally monophyletic with high support values (1.0/100).

Within Group I, the clade consisting of two specimens from central Vietnam (AMNH A-169287, Thua Thien-Hue Prov., and AMNH A-163668, Quang Nam Prov.: identified as *O. gerti* by Bain et al. 2007) forms a sister clade to all other species in this group (1.0/100). This lineage is determined to be distantly related to the topotype specimens of *O. gerti* from the Langbian Plateau, and based on examination of these specimens we tentatively regard these specimens as O. cf. poilani (see discussion below).

Within Group I, specimens of *O. microstoma* from southern China (KUH 311601) and northern Vietnam (AMCC 141231) are clustered together (1.0/100) forming a sister clade to a group comprising specimens identified as *O. hansi* Ohler, 2003, from Kon Tum Plateau in central Vietnam; monophyly of the latter group is also strongly supported (1.0/100) (Fig. 2).

Within Group II, Subclade A joins the medium-sized specimens from environs of the type locality of *O. gerti* (Langbian Mt., environs of Da Lat city, Lam Dong Prov., Vietnam, up to 1800 m a.s.l.; see Fig. 1: Loc. 5) with two other populations found at medium elevation (1000–1500 m a.s.l.) on the eastern (Bidoup–Nui Ba N.P., Lam Dong Prov., Vietnam, see Fig. 1: Loc. 6) and northern (Chu Yang Sin N.P., Dak Lak Prov., Vietnam; see Fig. 1: Loc. 11) edges of the Langbian Plateau (Fig. 2). In the present paper we treat this lineage as the species level clade representing *O. gerti* *s. stricto* (see discussion below).

The second species level clade comprises large-sized *Ophryophryne* from comparatively lowland populations in the western foothills of the Langbian Plateau (Fig. 1, Locs 1–4, < 1000 m a.s.l.) and large-sized *Ophryophryne* from medium elevations in the northern parts of the plateau (Fig. 1, Locs 6 and 11; 1000–1500 m a.s.l.). Two reciprocally monophyletic (1.0/100) subclades are revealed in this clade (Fig. 2). Subclade B joins montane populations from 1000–1500 m a.s.l. (Fig. 1, Locs 4, 6 and 11) and a recently discovered lowland (200 m a.s.l.) population from Cat Tien N.P. in Dong Nai Prov. Subclade C joins two populations from the westernmost edge of the Langbian Plateau, which include the holotype of *O. synoria* from Mondolkiri Prov. in eastern Cambodia (Fig. 1, Loc. 1; 500 m a.s.l.) and a population from Bu Gia Map N.P. in the adjacent Vietnamese province of Binh Phuoc (Fig. 1, Loc. 2; 400 m a.s.l.). These localities are close to each other and geographically belong to one hilly region on the western edge of the Langbian Plateau. Based on phylogenetic and morphological data we herein regard both subclades B and C as the species level clade representing *O. synoria*.

The third species level clade forms a sister clade with respect to a clade comprised of *O. gerti* *s. stricto* and *O. synoria* (Fig. 2). It joins small-sized *Ophryophryne* specimens, all collected from both high elevations (> 1750 m a.s.l.) in the northern and eastern parts of the Langbian Plateau (see Fig. 1: Bidoup and Hon Giao Mts., Lam Dong Prov., Locs 6–7; and Chu Pan Fan and Chu Yang Sin Mts., Dak Lak Prov., Locs 10–11), and from lower elevations (700–1510 m a.s.l.) on the summits of three mountains representing the easternmost outcrops of the Langbian Plateau (see Fig. 1: Hon Ba Mt., Khanh Hoa Prov., Loc. 8; Nui Chua Mt., Ninh Thuan Prov., Loc. 9; Tay Hoa, Phu Yen Prov., Loc. 12). Among these populations, samples from the summit of Nui Chua Mt. (Fig. 1, Loc. 9) form Subclade D (Fig. 2), forming a sister clade with respect to all other populations (Fig. 2, Subclade E; monophyly support 0.95/98). This clade of small-sized *Ophryophryne* from the northern and eastern parts of the Langbian Plateau currently cannot be assigned to any of the recognized species and represents a new species described herein.

### Intra- and interspecific differentiation of mtDNA haplotypes

The observed interspecific sequence divergence within the genus *Ophryophryne* varied from *p* = 4.1% to *p* = 13.0% (Table 2). The values of uncorrected genetic *p*-distances in ingroup and outgroup comparisons slightly overlapped: sequence divergence between *Ophryophryne* and outgroup taxa varied from *p* = 8.8% to *p* = 24.7%. The minimal interspecific *p*-distance between recognized nominal species in our analysis was found between the sister species *O. gerti* and *O. synoria* (*p* = 4.1%–5.0%). The maximum *p*-distance for *Ophryophryne* was observed between *O. synoria* and *O. hansi* (*p* = 12.6%–13.0%) (see Table 2). The ABGD analysis revealed the existence of a “barcoding gap” at genetic distance value *p* = 4.0% in the 16S rRNA gene.

**Table 2.** Uncorrected *p*-distance (percentage) between 16S rRNA sequences of *Ophryophryne* species and outgroup species included in phylogenetic analyses (below the diagonal), and standard error estimates (above the diagonal). The ingroup mean uncorrected *p*-distances are shown on the diagonal and shaded with grey.

	Species	1	2	3	4	5	6	7	8	9	10	11	12	13	14	15	16	17	18	19	20
**1**	*O. gerti* (Subclade A)	**0.5**	1.1	1.1	1.7	1.6	1.9	1.8	1.7	2.0	1.9	1.9	2.1	2.2	2.1	2.2	2.2	2.1	1.9	2.2	2.4
**2**	*O. synoria* (Subclade B)	4.1	**0.4**	0.9	1.6	1.6	1.9	1.8	1.8	1.8	1.8	1.7	2.0	2.1	2.1	2.2	2.2	2.0	1.9	2.2	2.3
**3**	*O. synoria* (Subclade C)	5.0	2.6	**0.9**	1.7	1.6	1.8	1.9	1.7	1.8	1.8	1.6	2.0	2.1	2.1	2.1	2.1	2.0	1.9	2.2	2.2
**4**	*O. elfina* sp. n. (Subclade D)	9.1	9.3	10.0	**0.0**	0.9	1.8	1.6	1.7	2.1	1.8	1.9	2.2	2.0	2.2	2.2	2.3	2.1	2.1	2.3	2.2
**5**	*O. elfina* sp. n. (Subclade E)	8.2	8.9	8.7	3.1	**0.8**	1.8	1.6	1.6	2.1	2.0	2.1	2.2	1.8	2.3	2.2	2.1	2.1	2.0	2.3	2.3
**6**	*O. microstoma*	10.9	11.8	11.6	9.7	9.5	**3.7**	1.3	1.5	1.6	1.9	1.7	1.7	1.8	1.9	2.1	2.2	2.0	1.9	2.4	2.4
**7**	*O. hansi*	10.9	12.6	13.0	9.7	10.0	7.6	**0.0**	1.3	1.7	1.8	1.7	1.9	1.8	2.1	2.2	2.3	1.9	1.8	2.4	2.5
**8**	O. cf. poilani	9.1	10.5	10.6	8.6	9.4	7.8	7.3	**3.3**	1.8	1.7	1.7	1.8	1.7	2.0	2.1	2.5	1.9	1.7	2.2	2.2
**9**	*Xenophrys minor*	12.8	13.0	12.7	10.3	10.9	8.0	10.8	8.8	–	1.8	1.4	1.9	2.0	2.0	2.2	2.1	1.5	1.7	2.3	2.4
**10**	*Xenophrys* sp.	11.6	12.2	11.9	11.1	11.7	10.3	9.5	8.8	7.8	–	1.3	1.8	1.8	1.8	2.2	2.4	1.7	1.7	2.2	2.2
**11**	*Xenophrys omeimontis*	10.8	11.4	11.0	9.9	10.2	8.8	9.6	8.4	5.3	3.7	–	1.7	1.8	1.8	2.1	2.1	1.2	1.6	2.2	2.2
**12**	Xenophrys cf. maosonensis	13.6	14.7	15.1	14.4	15.2	10.5	12.8	9.7	11.1	10.3	9.9	–	1.4	1.4	1.9	2.2	1.8	1.7	2.4	2.4
**13**	Xenophrys cf. parva	13.6	14.2	14.3	11.1	10.7	10.1	11.4	9.7	9.5	9.1	9.1	7.4	–	1.6	1.9	2.3	1.7	1.8	2.5	2.4
**14**	Xenophrys cf. daweimontis	12.8	13.4	14.3	14.8	14.5	11.3	14.0	11.5	11.1	9.5	9.1	6.6	6.2	–	2.0	2.3	2.0	2.1	2.6	2.3
**15**	Xenophrys cf. aceras	16.5	16.3	16.0	18.1	18.3	16.9	18.9	14.8	14.8	14.0	12.8	13.6	14.4	13.6	–	2.5	2.0	1.9	2.3	2.4
**16**	*Megophrys nasuta*	14.5	16.0	16.4	16.0	14.5	15.8	17.8	15.8	14.0	14.4	13.6	13.6	13.2	14.8	16.5	–	2.0	2.2	2.6	2.4
**17**	*Brachytarsophrys feae*	12.8	13.0	13.7	10.7	11.5	11.5	11.7	10.9	7.0	7.4	5.3	11.1	9.1	11.1	12.3	14.4	–	1.4	2.4	2.4
**18**	*Brachytarsophrys carinense*	11.5	11.4	12.0	11.5	11.5	11.5	11.3	10.1	8.6	7.4	7.4	10.3	9.5	11.9	12.3	12.8	4.9	–	2.2	2.3
**19**	*Leptolalax*	17.5	18.8	19.4	18.5	19.9	21.0	21.1	18.9	21.8	19.8	21.0	20.2	19.3	21.4	22.6	23.9	20.2	18.9	–	2.2
**20**	*Leptobrachium*	23.1	23.9	24.0	22.2	21.8	23.5	24.7	23.1	22.8	22.0	21.8	23.3	22.4	23.7	23.7	22.2	20.4	20.8	21.8	–

Intraspecific distances within *Ophryophryne* species in our analysis varied from *p* = 0.5% (in *O. gerti*), to *p* = 3.3% among two samples of O. cf. poilani from Quang Nam and Thua Thien-Hue provinces of Vietnam, and to *p* = 3.7% among two samples of *O. microstoma* from China and Vietnam respectively. The latter two values are higher than usual intraspecific distances in the 16S rRNA gene in Anura (Vences et al. 2005a, 2005b, Vieites et al. 2009); we recognize that identification of these lineages as conspecific is preliminary, based on morphology and topology of the mtDNA tree. Further studies are needed to clarify their taxonomic status. We also found significant genetic differentiation between intraspecific lineages in two of the three *Ophryophryne* species inhabiting the Langbian Plateau. Sequence divergence between Subclades B and C of *O. synoria* is *p* = 2.6%, while the differentiation between the Nui Chua population (Subclade D) and all other populations of the new species (Subclade E) was even greater at *p* = 3.1%.

The newly discovered lineage of *Ophryophryne* from highlands of the northern and eastern parts of the Langbian Plateau was found to have the lowest genetic distance with respect to *O. gerti* (*p* = 8.2%–9.1%). This value is much higher than the minimum genetic distances observed in intraspecific comparisons between species of *Ophryophryne* included in this study (Table 2).

### Morphological differentiation

**Figure 3.**
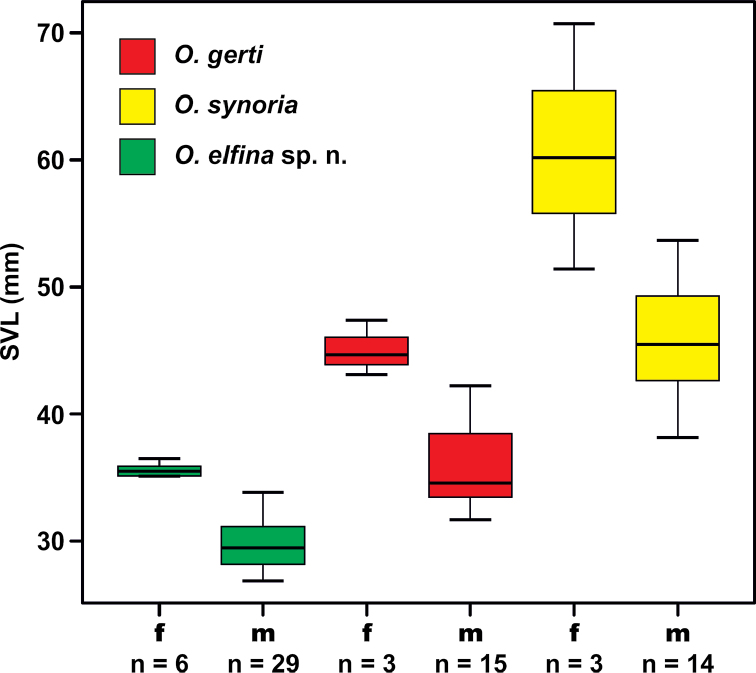
Boxplots of SVL showing body size variation among adult *Ophryophryne* males and females from the Langbian Plateau. Horizontal lines within each box represent the median, and boxes encompass the 75^th^ and 25^th^ quartiles. Color of boxes/*Ophryophryne* species corresponds to icon colors on Figs 1, 2.

Among the three species examined, mean SVL varied significantly, ranging from 26.9 to 53.7 mm in males and from 35.1 to 70.7 mm in females (Table 3). For SVL, *post hoc* analyses of one-way ANOVA revealed that males were significantly smaller than females in all three species of Langbian *Ophryophryne* (one-way ANOVA, p < 0.05; Duncan test, p < 0.05). Body size variation among adult males and females of Langbian *Ophryophryne* is shown in Figure 3. All three species are clearly different in body size, with *O. synoria* being the largest, and the undescribed *Ophryophryne* species being the smallest species known for the genus (male SVL values overlap with values for *O. pachyproctus*, N.A. Poyarkov, pers. observ.). *Ophryophryne gerti* occupies an intermediate position between these two species, with SVL values of males (31.7–42.2 mm) slightly overlapping both with those for *O. synoria* (38.2–53.7 mm) and the undescribed *Ophryophryne* species (26.9–33.9 mm).

**Table 3.** Measurements of the three species of *Ophryophryne* found on the Langbian Plateau (southern Vietnam) and in adjacent Cambodia. For abbreviations see Material and methods. Values are given as means ± SE (min–max), *N* — number of specimens. All measurements are given in mm (continues on next page).

**Species**	**SVL**	**HW**	**HL**	**ED**	**TYD**	**TYE**	**SL**	**EN**	**NS**	**IUE**	**IN**	**UEW**	**FAL**	**HAL**	**FIL**	**FIIL**	**FIIIL**	**FIVL**	**SHL**	**TL**	**FOL**	**TFOL**	**IMT**
*Ophryophryne gerti*												
Males	35.9 ± 3.4	10.3 ± 0.8	10.2 ± 0.9	4.4 ± 0.4	2.4 ± 0.4	2.0 ± 0.3	3.2 ± 0.5	1.4 ± 0.3	1.6 ± 0.3	3.3 ± 0.5	2.7 ± 0.4	3.1 ± 0.4	8.7 ± 1.0	8.7 ± 0.9	3.7 ± 0.6	3.7 ± 0.4	5.9 ± 0.6	3.5 ± 0.6	17.2 ± 2.8	16.8 ± 2.5	14.7 ± 2.4	23.7 ± 2.6	2.0 ± 0.5
*N* = 15	(31.7–42.2)	(9.2–12.0)	(9.0–12.0)	(3.8–5.3)	(1.8–3.1)	(1.7–2.5)	(2.6–3.8)	(0.7–1.8)	(1.1–2.2)	(2.2–4.1)	(1.9–3.4)	(2.2–3.8)	(7.2–11.0)	(7.2–10.1)	(2.7–5.0)	(3.1–4.4)	(4.2–7.2)	(2.3–4.6)	(14.4–24.6)	(13.9–23.3)	(11.2–18.8)	(19.2–27.6)	(0.9–2.7)
Females	45.1 ± 2.2	11.3 ± 0.4	11.4 ± 0.6	4.8 ± 0.4	2.7 ± 0.2	2.5 ± 0.2	3.4 ± 0.4	1.6 ± 0.3	1.7 ± 0.4	3.8 ± 0.2	2.8 ± 0.1	3.9 ± 0.3	10.4 ± 0.6	10.5 ± 1.4	4.7 ± 0.7	4.3 ± 0.6	7.2 ± 0.5	4.6 ± 0.7	19.7 ± 1.0	19.6 ± 1.6	16.7 ± 0.5	27.0 ± 0.9	2.9 ± 0.2
*N* = 3	(43.1–47.4)	(10.9–11.7)	(10.9–12.1)	(4.4–5.1)	(2.5–3.0)	(2.3–2.6)	(2.9–3.6)	(1.2–1.9)	(1.4–2.2)	(3.6–4.0)	(2.7–2.9)	(3.5–4.2)	(9.8–10.9)	(9.0–11.9)	(3.9–5.3)	(3.8–4.9)	(6.7–7.7)	(4.1–5.4)	(18.7–20.7)	(17.8–20.6)	(16.3–17.2)	(26.0–27.9)	(2.6–3.0)
*Ophryophryne elfina* sp. n.												
Males	29.7 ± 1.8	8.6 ± 0.7	8.9 ± 0.6	3.7 ± 0.3	2.2 ± 0.2	1.5 ± 0.2	2.7 ± 0.4	1.2 ± 0.2	1.4 ± 0.3	2.7 ± 0.3	2.3 ± 0.5	2.9 ± 1.0	7.3 ± 0.8	7.0 ± 0.9	2.6 ± 0.5	2.9 ± 0.4	4.8 ± 0.5	3.0 ± 0.6	14.0 ± 1.7	13.7 ± 1.3	11.9 ± 1.0	19.8 ± 2.2	1.6 ± 0.4
*N* = 29	(26.9–33.9)	(7.2–10.1)	(7.4–9.8)	(3.1–4.3)	(1.6–2.7)	(1.1–2.1)	(2.0–3.3)	(0.8–1.6)	(1.0–2.2)	(2.1–3.4)	(1.5–3.3)	(2.4–7.9)	(6.1–8.9)	(4.8–9.4)	(1.1–3.5)	(2.0–4.1)	(3.7–6.0)	(1.4–4.1)	(11.7–19.6)	(11.3–16.1)	(10.4–14.7)	(17.1–28.0)	(1.2–2.5)
Females	35.6 ± 0.5	9.7 ± 0.5	9.8 ± 1.1	4.0 ± 0.2	2.3 ± 0.4	1.7 ± 0.1	3.1 ± 0.5	1.4 ± 0.3	1.7 ± 0.3	2.9 ± 0.5	2.5 ± 0.4	2.9 ± 0.2	9.0 ± 1.1	8.8 ± 0.7	3.7 ± 0.7	3.9 ± 0.5	5.8 ± 0.8	4.1 ± 0.9	17.1 ± 2.0	16.3 ± 1.0	14.9 ± 0.7	24.3 ± 2.1	2.0 ± 0.3
*N* = 6	(35.1–36.5)	(9.0–10.2)	(8.6–11.0)	(3.6–4.2)	(1.9–3.0)	(1.6–1.9)	(2.4–3.6)	(1.0–1.7)	(1.2–2.1)	(2.3–3.6)	(1.7–2.9)	(2.6–3.1)	(7.7–10.1)	(7.7–9.7)	(2.9–4.5)	(3.3–4.7)	(4.9–7.0)	(3.1–5.2)	(14.9–19.8)	(15.5–17.8)	(13.9–15.7)	(21.8–27.2)	(1.5–2.4)
*Ophryophryne synoria*												
Males	45.7 ± 4.3	13.8 ± 0.9	13.5 ± 1.0	5.1 ± 0.3	3.5 ± 0.5	2.8 ± 0.4	3.8 ± 0.5	1.9 ± 0.4	1.6 ± 0.3	4.5 ± 0.6	3.1 ± 0.3	3.7 ± 0.4	12.6 ± 1.3	11.0 ± 1.1	4.6 ± 0.6	4.7 ± 0.6	7.5 ± 0.6	4.5 ± 0.6	20.6 ± 1.6	20.7 ± 2.2	18.4 ± 2.0	29.3 ± 2.8	2.5 ± 0.4
*N* = 14	(38.2–53.7)	(12.5–15.6)	(12.2–15.4)	(4.7–5.6)	(3.0–4.7)	(1.9–3.6)	(3.0–4.6)	(1.3–2.5)	(1.2–2.2)	(3.5–5.5)	(2.5–3.5)	(3.0–4.1)	(10.4–14.7)	(9.2–11.1)	(3.7–5.8)	(3.7–5.6)	(6.2–8.4)	(3.6–5.5)	(17.7–23.3)	(18.1–25.1)	(15.0–22.4)	(24.0–33.5)	(1.9–3.1)
Females	60.8 ± 9.7	16.6 ± 1.8	16.1 ± 1.8	5.3 ± 0.4	3.7 ± 0.2	3.3 ± 0.3	4.0 ± 0.3	2.0 ± 0.4	1.8 ± 0.4	4.7 ± 0.2	3.5 ± 0.6	4.1 ± 0.2	14.3 ± 1.0	13.7 ± 3.3	5.1 ± 0.5	5.9 ± 1.0	8.4 ± 0.9	5.1 ± 1.4	23.4 ± 2.0	24.3 ± 1.5	20.7 ± 4.6	32.3 ± 5.0	3.2 ± 0.4
*N* = 3	(51.4–70.7)	(14.6–18.0)	(14.0–17.4)	(5.0–5.7)	(3.4–3.9)	(3.0–3.6)	(3.7–4.3)	(1.6–2.4)	(1.3–2.2)	(4.4–4.9)	(3.0–4.1)	(3.8–4.2)	(13.6–15.5)	(11.0–17.4)	(4.7–5.7)	(5.1–7.0)	(7.6–9.3)	(4.3–6.7)	(21.3–25.3)	(22.7–25.7)	(15.7–24.8)	(26.8–36.4)	(2.8–3.6)

**Figure 4.**
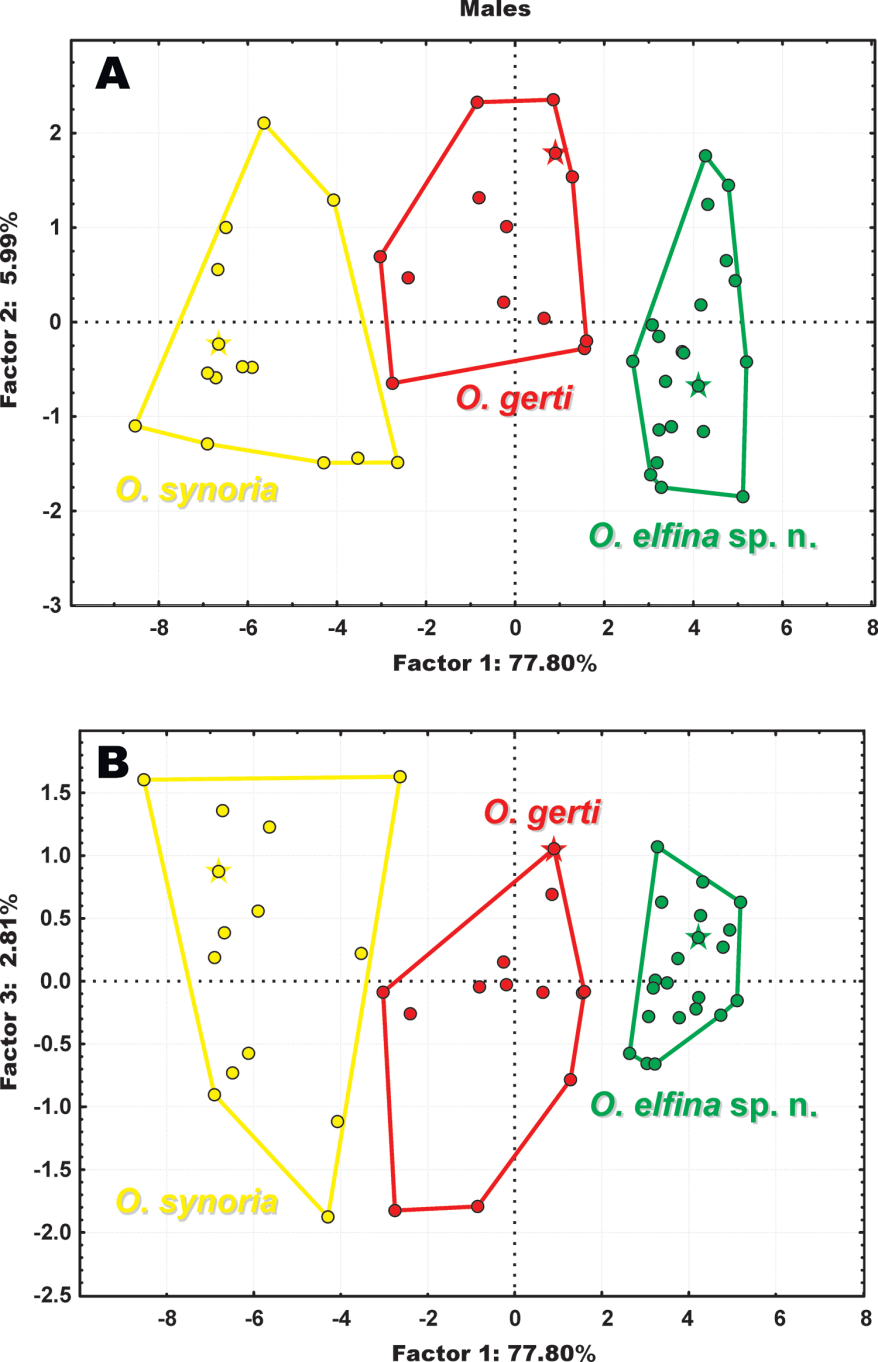
Two-dimensional plots of **A** the first two factors of PCA, and **B** the first and third factors of morphological characters for the *Ophryophryne* specimens examined. Data are given for males only. Star indicates the holotype specimen for each species. Circle color corresponds to those given in Fig. 1.

The results of the multivariate PCA-analysis of the morphometric data are shown in Fig. 4 (data given for males only). The discriminative power of PCA factors derived from analysis of morphometric characters is shown in Appendix 2. For males, F1 explained 77.80% of the variability and F2 explained 5.99%. The two-dimensional plots of both the first two principal components (Factor 1 and Factor 2; Fig. 4A) and the first vs. the third principal components (Factor 1 and Factor 3; Fig. 4B) for males completely discriminated the following three morpho-groups: (I) *O. gerti*, (II) *O. synoria*, and (III) the undescribed *Ophryophryne* species. Our multivariate analysis included meristic data for the holotypes of two *Ophryophryne* species: *O. synoria* (FMNH 262779, male) and *O. gerti* (BMNH 1921.4.1.324, male). Both holotypes were assigned correctly to the respective groups, representing *O. synoria* and *O. gerti*, in full concordance with the results of molecular analyses (Fig. 4).

In summary, both in body size and other meristic characters, all three examined lineages of Langbian *Ophryophryne* form clearly separated morphological groups, also supported by multivariate statistical analysis. The small-sized population of the undescribed *Ophryophryne* species appears to be morphologically distinct from sympatric *O. gerti* and *O. synoria*, as well as from other congeners (see Comparisons for details).

### Acoustic differentiation

**Figure 5.**
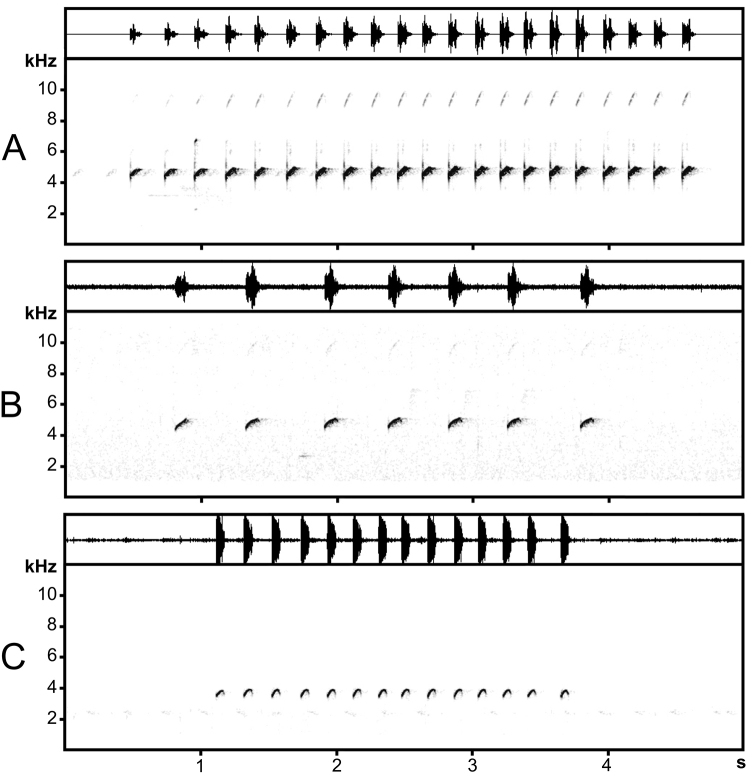
Oscillograms (top) and sonograms (bottom) of male advertisement calls of **A** *Ophryophryne elfina* sp. n. (Lam Dong Prov., Bidoup-Nui Ba N.P., 1935 m a.s.l., 17°C) **B** *O. gerti* (Dak Lak Prov., Chu Yang Sin N.P., 1020 m a.s.l., 22°C) **C** *O. synoria* (Dak Lak Prov., Chu Yang Sin N.P., 750 m a.s.l., 21°C). The sampling rate lowered to 22.05 kHz.

Measurements of advertisement call parameters for three *Ophryophryne* species found on the Langbian Plateau are given in Table 4, oscillograms and sonograms are given in Fig. 5. Advertisement calls of all three *Ophryophryne* species found on the Langbian Plateau represent a series of high whistling sounds, resembling vocalizations of passerine birds. All males vocalized in the evening from dusk until late night. The males of the undescribed *Ophryophryne* species called from stone walls above small streams or from fern leaves, the males of *Ophryophryne gerti* usually called while sitting on bush branches and fern leaves above small streams, and the males of *Ophryophryne synoria* called from stones on the rocky stream banks.

**Table 4.** Measurements of advertisement call parameters for three species of *Ophryophryne* from the Langbian Plateau, and one-way ANOVA/Kruskal-Wallis results for comparison (*p < 0.001) between *Ophryophryne elfina* sp. n., *O. gerti* and *O. synoria*. Parameter values are given as means ± SE (min–max). Abbreviations: *N* — number of recordings/series/calls, F — frequency, s — seconds, ms — milliseconds, Hz — hertz.

Parameters	*O. elfina* sp. n.	*O. gerti*	Tukey/ Mann-Whitney U post hoc tests	*O. synoria*	Tukey/ Mann-Whitney U post hoc tests	ANOVA/ Kruskal-Wallis results
Number of males	3	3	–	3	–	–
Number of recordings	5	3	–	3	–	–
Number of series	140	115	–	15	–	–
Number of calls	1797	533	–	200	–	–
Call repetition rate per recording (calls/s)	1.18 ± 0.2 (0.77–1.95) *N* = 5	0.35 ± 0.14 (0.11–0.59) *N* = 3	p < 0.05	3.07 ± 0.13 (2.82–3.24) *N* = 3	p < 0.001	F_2.8_ = 46.7*
Number of calls per series	12.84 ± 0.41 (2–22) *N* = 140	4.64 ± 0.16 (1–8) *N* = 115	p *<* 0.001	13.33 ± 1.4 (3–24) *N* = 15	p < 0.05	F_2.267_ = 151.4*
Series duration (s)	3.42 ± 0.11 (0.43–9.00) *N* = 140	2.18 ± 0.09 (0.57–8.31) *N* = 108	p < 0.001	2.59 ± 0.33 (0.62–5.21) *N* = 15	p = 0.88	F_2.267_ = 40.4*
Call repetition rate per series (calls/s)	3.87 ± 0.07 (1.33–5.49) *N* = 140	2.33 ± 0.03 (0.96–3.53) *N* = 108	p < 0.001	5.34 ± 0.15 (4.4–6.59) *N* = 15	p < 0.001	F_2.260_ = 220.7*
Call duration (ms)	73 ± 0.23 (25–112) *N* = 1797	104 ± 0.56 (75–152) *N* = 533	p < 0.001 (U = 12535.5)	62 ± 0.46 (37–85) *N* = 200	p < 0.001 (U = 64227.5)	H_2.2530_ = 1345.1*
Inter-calls interval (ms)	207 ± 2.06 (96–942) *N* = 1657	421.54 ± 4.17 (275–813) *N* = 418	p < 0.001 (U = 24725.5)	143 ± 3.32 (56–528) *N* = 185	p < 0.001 (U = 64860)	H_2.2260_ = 1008.5*
Inter-series interval (s)	6.51 ± 0.41 (1.26–31.65) *N* = 135	7.98 ± 0.55 (0.65–39.76) *N* = 112	p < 0.001 (U = 5593.5)	1.64 ± 0.19 (0.88–2.83) *N* = 12	p < 0.001 (U = 51)	H_2.259_ = 42.7*
F initial (Hz)	4348.02 ± 2.96 (3980–4680) *N* = 1797	4414.17 ± 5.12 (4070–4640) *N* = 533	p < 0.001 (U = 332858)	3449.55 ± 6.41 (3230–3700) *N* = 200	p < 0.001 (U = 0)	H_2.2530_ = 655.7*
F final (Hz)	4715.3 ± 3.29 (4260–5010) *N* = 1797	4888.76 ± 3.82 (4640–5150) *N* = 533	p < 0.001 (U = 142738)	3708.9 ± 9.28 (3420–3980) *N* = 200	p < 0.001 (U = 0)	H_2.2530_ = 1075.9*
F maximum (Hz)	4807.74 ± 3.46 (4260–5060) *N* = 1797	4998.74 ± 4.27 (4780–5250) *N* = 533	p < 0.001 (U = 138045)	3907.05 ± 4.22 (3750–4070) *N* = 200	p < 0.001 (U = 0)	H_2.2530_ = 1094.2*
F minimum (Hz)	4348.02 ± 2.96 (3980–4680) *N* = 1797	4414.17 ± 5.12 (4070–4640) *N* = 533	p < 0.001 (U = 309752.5)	3449.55 ± 6.41 (3230–3700) *N* = 200	p < 0.001 (U = 0)	H_2.2530_ = 689.5*
F range (Hz)	459.71 ± 3.27 (40–840) *N* = 1797	584.58 ± 3.87 (370–890) *N* = 533	p < 0.001 (U = 229934)	457.5 ± 5.86 (230–650) *N* = 200	p = 0.61 (U = 175721)	H_2.2530_ = 367.1*
F peak (Hz)	4645.94 ± 4.39 (4030–4920) *N* = 1797	4845.99 ± 4.22 (4450–5100) *N* = 533	p < 0.001 (U = 157981.5)	3798.9 ± 4.87 (3600–3890) *N* = 200	p < 0.001 (U = 0)	H_2.2530_ = 1030.2*

Advertisement calls of all studied species were similarly uttered in series (Fig. 5). The call repetition rate/recording/series were one of the most significant differentiating parameters between the three species (Table 4). Parameters values varied both between and within recordings for each of the three species. Though some parameters values overlapped between species, the means of most of the parameters differed significantly.

The frequency of maximum amplitude always coincided with the fundamental frequency and greatly varied within recordings: from 4030 to 4920 Hz for the undescribed *Ophryophryne* species, from 4450 to 5100 Hz for *O. gerti*, and from 3600 to 3890 Hz for *O. synoria*. The values of the maximum amplitude frequency in *O. synoria* were the lowest and least variable among the three species. The minimum fundamental frequency always coincided with the initial fundamental frequency whereas the maximum fundamental frequency either could coincide with the final fundamental frequency, or was close to it. Thus, the frequency modulation was expressed either in lift of fundamental frequency during the whole call, or in an unsymmetrical arch with the peak shifted to the end of the frequency band. The form of frequency modulation varied between these two forms for each species but arch-formed calls appeared most of time in *O. synoria*. The frequency range expressing depth of frequency modulation also varied within each species’ calls: from 40 to 840 Hz for the undescribed *Ophryophryne* species, from 370 to 890 Hz for *O. gerti* and from 230 to 650 Hz for *O. synoria* (Table 4).

Number of harmonics varied between/within recordings but this characteristic mostly depended on recording quality (e.g., sensitivity of recording equipment, distance from vocalizing animal, signal volume and background noise). Calls from the highest quality recordings (of the undescribed *Ophryophryne* species) contained two harmonics but a major portion of other calls contained only one harmonic.

We had two sets of the undescribed *Ophryophryne* species recordings which were made at different temperatures (11.3–11.4°C in February and 17.0–17.5°C in April). Values of frequency call parameters didn’t significantly differ between the two sets. However, statistically significant differences were found in several temporal parameters of the calls (data summarized in Appendix 3). For instance, the number of calls per series was 10.53 ± 0.72 (3–21, *N* = 47) in February vs. 14 ± 0.45 (2–22, *N* = 93) in April (F_1.138_ = 18.2, p < 0.001, one-way ANOVA), the inter-call duration comprised 271 ± 4.92 ms (102–942 ms, *N* = 448) in February vs. 184 ms ± 1.72 (96–621 ms, N = 1209) in April (H_1.1657_ = 349.2, p < 0.001, Kruskal-Wallis ANOVA), the call repetition rate per series was 3.18 ± 0.1 calls/s (1.33–4.91 calls/s, *N* = 47) in February vs. 4.22 ± 0.08 calls/s (2.92–5.49 calls/s, *N* = 93) in April (F_1.138_ = 63.9, p < 0.001, one-way ANOVA), call duration was also significantly longer in February 79 ± 0.32 ms (48–102 ms, *N* = 496) than in April 70 ± 0.27 ms (25–112 ms, *N* = 1301) (H_1.1798_ = 380.2, p < 0.001, Kruskal-Wallis ANOVA). April and February recordings showed no significant differences in series duration, inter-series interval duration, and call repetition rate per recording.

### Taxonomic implications

**Figure 6.**
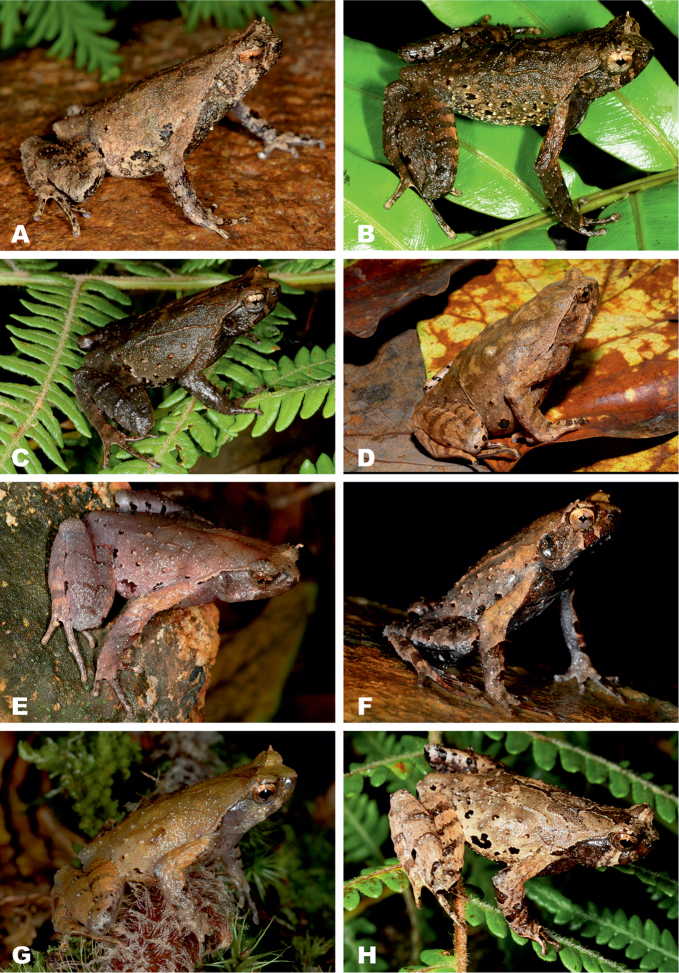
*Ophryophryne* species of the Langbian Plateau (Vietnam): **A** *O. gerti*, female, Chu Yang Sin N.P., Dak Lak Prov., 1000 m a.s.l. **B** *O. gerti*, male, Chu Yang Sin N.P., Dak Lak Prov., 1000 m a.s.l. **C** *O. gerti*, male, Bidoup–Nui Ba N.P., Lam Dong Prov., 1550 m a.s.l. **D** *O. synoria*, female, Bu Gia Map N.P., Binh Phuoc Prov., 400 m a.s.l. **E** *O. synoria*, male, Chu Yang Sin Mt., Chu Yang Sin N.P., Dak Lak Prov., 1000 m a.s.l. **F** *O. synoria*, male, Bidoup–Nui Ba N.P., Lam Dong Prov., 1550 m a.s.l. **G** *Ophryophryne elfina* sp. n., male, Chu Yang Sin Mt., Chu Yang Sin N.P., Dak Lak Prov., 2000 m a.s.l. **H** *Ophryophryne elfina* sp. n., male, Hon Giao Mt., Bidoup–Nui Ba N.P., Lam Dong and Khanh Hoa provincial border, 2000 m a.s.l. Photos by N.A. Poyarkov and N.L. Orlov.

Our study, based on three lines of evidence — phylogenetic analysis and distribution of mtDNA haplotypes (Figs 1, 2), multivariate statistical analysis of 23 standard morphometric traits (Fig. 4), and acoustic analysis of advertisement calls (Fig. 5), strongly indicate the presence of three independent and distinct evolutionary lineages of *Ophryophryne* on the Langbian Plateau and in surrounding areas of southern Truong Son Mountains in southern Vietnam and eastern Cambodia. Our examination of type material allowed us to identify the two larger lineages as *O. synoria* and *O. gerti*, whereas the smaller species of *Ophryophryne* represents an undescribed taxon (Fig. 6). In two of the 12 surveyed localities (see Fig. 1) all three species were recorded in sympatry, with *O. synoria* and *O. gerti* recorded syntopically in the same streams in environs of Giang Ly Ranger Station, Bidoup–Nui Ba N.P., Lam Dong Prov. (Loc. 6, Fig. 1) and in Chu Yang Sin N.P. in Dak Lak Prov. (Loc. 11, Fig. 1); the small-sized undescribed *Ophryophryne* species was usually found in higher elevations, but was nevertheless recorded synbiotopically with *O. gerti* in Chu Yang Sin N.P. in Dak Lak Prov. (Loc. 11, Fig. 1). Below we provide taxonomic remarks on *Ophryophryne* species of the Langbian Plateau along with the description of the new species.

#### Taxonomic remarks on Ophryophryne gerti

Taxon classificationAnimaliaAnuraMegophryidae

Ohler, 2003

##### Chresonymy:

[?] *Ophryophryne microstoma* — Orlov et al. 2002:84 (partim––Dac Lac [Dak Lak] and Lam Dong provinces, Vietnam; no reffered specimens indicated, inclusion *fide* N.L. Orlov).

*Ophryophryne gerti* Ohler 2003:25, fig. 1 (partim––BMNH 1921.4.1.324, BMNH 1921.4.1.323).

[?] *Ophryophryne gerti* — Ohler 2003:25; Stuart et al., 2006:135 (FMNH 252899, FMNH 252901).

[?] *Ophryophryne microstoma* — Nguyen et al. 2005:15 (partim––“Dak Lak and Lam Dong” provinces, Vietnam; following indication by Orlov et al. 2002).

“*Ophryophryne* sp. 2” — Orlov et al. 2008:82 (Chu Yang Sin N.P., Dak Lak Prov., Vietnam; ZISP 12836–12879).

*Ophryophryne gerti* — Nguyen et al. 2009:84–85 (partim––“Lam Dong (Cam Li, Dran)”, Vietnam).

[?] *Ophryophryne microstoma* — Nguyen et al. 2009:86 (partim––“Dak Lak, Lam Dong”, Vietnam; based on the record by Orlov et al. 2002).

[?] *Ophryophryne gerti* — Stuart et al. 2010:40 (eight uncataloged “topotype” females from Bidoup–Nui Ba N.P., Langbian Plateau, Vietnam).

*Ophryophryne gerti* — Poyarkov [Paiarkov] and Vassilieva 2011:202 (Bidoup–Nui Ba N.P., Lam Dong Prov., Vietnam; ZMMU A-4715, ZMMU A-4718).

##### Removed from chresonymy:

*Ophryophryne gerti* Ohler 2003:25 (partim––BMNH “1972.15.2.4” [sic. BMNH 1972.1524])

*Ophryophryne gerti* — Stuart 2005:475 (FMNH 258564)

*Ophryophryne gerti* — Bain et al. 2007:108 (AMNH A-169287, AMNH A-163668)

[?] *Ophryophryne gerti* — Orlov et al. 2015:2010, fig. 9 (Gia Lai Prov., Vietnam; based on identification of the specimen illustrated in fig. 9 as *Ophryophryne* cf. *poilani sensu auctorum*).

##### Holotype.

BMNH 1921.4.1.324, adult male from “Cam Ly (river), south-east of Da Lat (11°56'N; 108°25'E), Lang Bian Plateau, sLam [sic. = Lam] Dong Province, Vietnam”, collected by M.A. Smith, presented to BMNH in 1921 (Ohler 2003; NHMUK specimen catalogue). Re-examined by SM.

##### Paratypes.

BMNH 1921.4.1.323, immature female from “Dran (11°50'N; 108°34'E), Lang Bian Plateau, Lam Dong Province, Vietnam”, collected by M.A. Smith, presented to BMNH in 1921; BMNH 1972.1524 (see ‘Remarks’) adult male, “Huey Sapan, Pak Maat (precise location not found), Mekong, Laos”, collector M.A. Smith, accessioned in the BMNH from Smith’s private collection in 1972, collection date unknown (Ohler 2003; NHMUK specimen catalogue).

##### Remarks.

Ohler (2003) provides the paratype number BMNH 1972.15.2.4 for the Laos paratype specimen, however, the number on the specimen tag and NHMUK specimen catalogue reads BMNH 1972.1524 (Mahony 2011b). Our present data suggests that *O. gerti* is not found beyond the limits of the Langbian Plateau, thus we are confident that the paratype BMNH 1972.1524 does not represent this biological taxon. Ohler (2003: and by implication Stuart et al. 2006) preliminarily identified two specimens from Ankhe Dist. in northern Gia Lai Prov. as *O. gerti*. This locality is disconnected from the Langbian highlands by a wide lowland area, indicating that these specimens are biogeographically isolated from *O. gerti* *s. stricto.* Further work is necessary to ascertain the taxanomic status of these specimens. Orlov et al. (2002), without providing data on examined specimens, considered that the distribution of *O. microstoma* extended south in Vietnam as far as the Lam Dong and Dak Lak provinces. Our results suggest that these southern Vietnamese populations most likely represented the superficially similar *O. gerti*, *O. synoria*, or possibly the new taxon described below. Stuart (2005) reports *O. gerti* from Champasak Prov. in southern Laos, based on a single specimen (FMNH 258564: not examined here). This locality is biogeographically not connected to the known range of this species, as redefined here, thus the taxonomic status of this specimen requires further confirmation. Bain et al. (2007) identifies two specimens AMNH A-169287 (Thua Thien-Hue Prov., Vietnam) and AMNH A-163668 (Quang Nam Prov., Vietnam) as *O. gerti*. We re-examined these specimens and regard them to be morphologically more similar to *O. poilani*. Furthemore, both specimens were included in our molecular analysis and found to be distantly related to *O. gerti* *s. stricto* (Fig. 2). Stuart et al. (2010) provides the SVL range (SVL 37.5–42.5 mm, mean ± SD 40.4 ± 1.6, *N* = 8) for ‘topotype’ female specimens of *O.* “*gerti*”, however these specimens are smaller than females of *O. gerti* provided herein (SVL 43.1–47.4 mm, mean ± SD 45.07 ± 2.16, *N* = 3), and larger than the new taxon described below (SVL 35.1–36.5 mm, mean ± SD 35.6 ± 0.5, *N* = 6) (see Table 3 for details). The taxonomic status of these specimens remains unknown.

##### Vernacular name.

English: “*Gerti’s Mountain Toad*”; Vietnamese: “*Cóc Núi Got*” (Nguyen et al. 2009), “*Cóc Núi Goti*” (Nguyen et al. 2014).

##### Redescription of the holotype.

**Figure 7.**
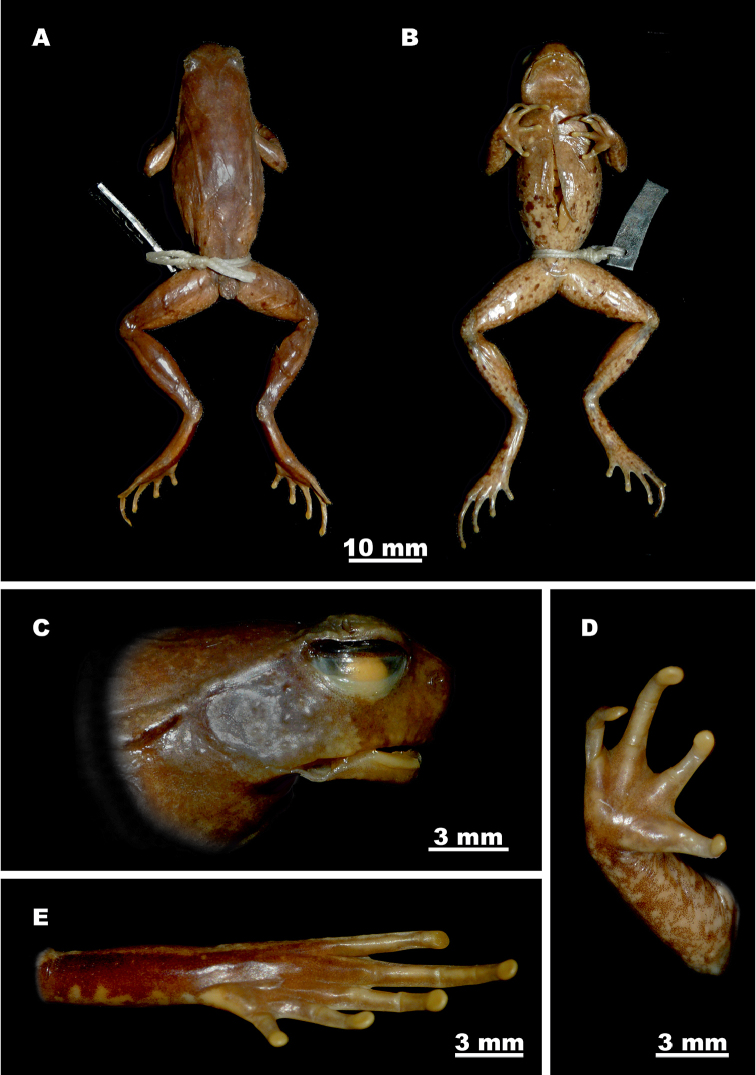
Holotype *Ophryophryne gerti* (BMNH 1921.4.1.324, male) in preservative: **A** dorsal view **B** ventral view **C** head, lateral view **D** volar view of the left hand **E** plantar view of the left foot. Photos by S. Mahony.

Mature male (SVL 35.7 mm), habitus slender (Fig. 7A, B). Specimen in good state of preservation; two incisions are present on trunk, one longitudinally orientated on mid-abdomen, another longitudinally oriented on upper flank on right side; liver and testes observable through incisions, testes enlarged; jaw is dislocated on right allowing visual access to buccal cavity.

Head moderately small (HL/SVL 25.5%; HW/SVL 29.7%), wider than long (HW/HL 116.5%), relatively deep; top of head flat; snout comparatively short (SL/HL 33.0%), truncated in dorsal view (Fig. 7A), projecting significantly beyond margin of lower jaw in profile (Fig. 7C); loreal region vertical; canthus rostralis distinct, moderately sharp; dorsal region of snout flat; eyes large (ED/HL 38.5%), slightly protuberant in dorsal view and in profile, horizontal eye diameter less than twice as long as maximum tympanum diameter (TYD/ED 62.9%) and longer than snout (ED/SL 116.7%); eye to tympanum distance approximately equal to maximum tympanum diameter (TYE/TYD 95.5%); tympanum distinct, circular, moderately large (TYD/HL 24.2%); pupil in preservation oval (Fig. 7C), vertically orientated; nostril opening oval, vertical, laterally orientated, medially located between eye and snout (EN/NS 100.0%); internarial distance subequal to upper eyelid width (IN/UEW 103.2%), and significantly less than narrowest point between upper eyelids (IN/IUE 145.5%); pineal ocellus not visible externally; vomerine ridges absent; maxillary and vomerine teeth absent; vocal sac gular, its’ openings not discernable; tongue moderately large, posterior end free, with weak notch posteriorly, lacking medial lingual process.

Forelimbs slender, forearm moderately long (FAL/SVL 22.4%) slightly enlarged relative to upper forelimb, and shorter than hand (FAL/HAL 95.2%); fingers long and narrow, dorsoventrally flattened; lateral fringes and webbing absent (Fig. 7D); finger length formula F1 = F2 < F4 < F3; subarticular tubercles absent, replaced by low callous dermal ridges; supernumerary tubercles absent; outer palmar (metacarpal) tubercle weak, longitudinally oval, elevated but with indistinct borders (Fig. 7D); thenar tubercles weak; finger tips rounded, weakly expanded relative to digit widths (wider than distal-most finger articulation), with circular pads (Fig. 7D); terminal grooves absent.

Hindlimbs slender, relatively long, shanks overlap when thighs are held at right angle to body; shank length less than half of snout to vent length (SHL/SVL 44.5%); thighs shorter than shanks (SHL/TL 106.0%), and feet (FOL/TL 106.4%); toes long and slightly dorsoventrally flattened (Fig. 7E), relative toe lengths T1 < T2 < T5 < T3 < T4; lateral fringes on toes, outer metatarsal tubercle, subarticular and supernumerary tubercles absent; inner metatarsal tubercle well developed with distinct borders, oval-shaped (IMT/FOL 11.3%) (Fig. 7E); weak ridge of callous tissue present on ventral surface of all toes, not continuing onto metatarsus; webbing between digits rudimentary; tarsal fold absent; toe tips not expanded relative to digit widths, with circular pads; terminal grooves absent.

##### Skin texture and skin glands in preservation.

Skin of dorsal and lateral surfaces of head, body and limbs smooth with numerous small tubercles finely and relatively evenly scattered on dorsal surfaces of trunk, head and limbs (Fig. 7A); small tubercles present on temporal region, tympanum smooth with borders weakly raised; tubercles arranged in distinct transverse ridges on dorsal surfaces of forearms, shanks and thighs; numerous large tubercles on flanks irregularly scattered from axilla to groin, intermixed with smaller tubercles; central portion of outer edge of upper eyelids slightly thickened, with a single short tubercular spine (Fig. 7C), transverse fold on posterior edges of upper eyelids absent; well-developed glandular supratympanic folds, narrow anteriorly, considerably widening posteriorly, from posterior corner of orbits, extending along upper margin of tympanum, terminating above forelimb insertions (Fig. 7C); dorsolateral glandular ridge well-developed, extending from posterior to supratympanic ridges to ca. 75% of trunk length, on each side; a moderately well-developed “ >–< ” shaped glandular parietoscapular-sacral ridge present on dorsum (see Fig. 7A); two small tubercles present above vent; gular region, chest, and ventral surfaces of limbs smooth to weakly shagreened, abdomen weakly granular; two nuptial pads per limb, one large on dorsal surface of F1 from base of metacarpal to near distal joint, another small pad on inner dorsal surface of F2 on metacarpal; pectoral glands round, weakly raised, positioned level with axilla; femoral glands slightly raised, average size, on posterior surface of thighs, situated slightly closer to knee than to cloaca; numerous small white asperities present on posterior half of dorsum, sparse anteriorly, increasing in density posteriorly to above cloaca, absent from all remaining surfaces.

##### Color of holotype in preservative

(Figure 7). Dorsal surfaces of head, body, forearms and hindlimbs mid to light brown, slightly lighter on flanks and dorsal surface of upper arms; a distinct darker brown “V”-shaped marking on dorsal surface of head; no distinct “X”-shaped or hourglass marking on mid dorsum; most flank tubercles are bordered below by a small dark brown spot anteriorly, increasing in size posteriorly towards groin; a broad brown stripe extends around lateral surfaces of snout, from anterior borders of orbits between canthus rostralis and the level of lower border of nostrils; two broad darker brown vertical stripes below orbits, one at level of anterior orbital border, and a second extends from central lower border of orbits to edge of jaw, a faint darker brown stripe extends from posterior border of orbits to cover tympanum; color of supratympanic folds same as surrounding sufaces, but lower border dark brown; edge of lower eyelid dark brown; dorsal surfaces of forearms each with two dark brown transverse blotches, and thighs and shanks with faint darker brown transverse stripes; ventral surfaces of throat, chest and anterior half of abdomen, and ventral surface of hands primarily plain light brown, fading to a mottled brownish beige with small dark brown blotches on posterior half of abdomen, and ventral surfaces of forelimbs, thighs and shanks, and dorsal surfaces of tarsi and feet; ventral surface of tarsi and feet dark brown fading distally on toes to a mid-brown; area surrounding cloaca dark brown, fading distally on lateral surfaces of thighs.

##### Measurements of the holotype

**(all in mm, taken by SM).** SVL 35.7; HW 10.6; HL 9.1; IFE 5.0; IBE 8.2; ED 3.5; TYD 2.2; TYE 2.1; SL 3.0; EN 1.5; NS 1.5; IUE 2.2; IN 3.2; UEW 3.1; FAL 8.0; HAL 8.4; FIL 3.4; FIIL 3.4; FIIIL 5.6; FIVL 3.6; SHL 15.9; TL 15.0; FOL 14.1; TFOL 21.7; IMT 1.6; TIL 1.7; TIIL 3.8; TIIIL 5.5; TIVL 6.7; TVL 3.5.

##### Distribution.

*Ophryophryne gerti* is herein confirmed from three localities on the Langbian Plateau in southern Vietnam, between 700–2000 m a.s.l. (Fig. 1): Cam Ly River and Nui Ba Mt. in environs of Dalat city, Lam Dong Prov., Vietnam (1000–1800 m a.s.l.) (Ohler 2003, this study); Environs of Bidoup Mt. (2000 m a.s.l.), and Giang Ly Ranger Station (1500 m a.s.l.), Bidoup–Nui Ba N.P., Lam Dong Prov., Vietnam (this study); Chu Yang Sin Mt. environs, Krong Kmar Commune, Krong Bong Dist., Dak Lak Prov., Vietnam (700–2000 m a.s.l.) (Orlov et al. 2008; this study). Additional localities reported in literature require confirmation pending further study of voucher material (see Remarks section above).

#### New records and range extension for Ophryophryne synoria

Taxon classificationAnimaliaAnuraMegophryidae

Stuart, Sok & Neang, 2006

##### Chresonymy:

*Ophryophryne synoria* Stuart et al. 2006:135, fig. 5.

“*Ophryophryne* sp. 3” — Orlov et al. 2008:82 (Chu Yang Sin N.P., Dak Lak Prov., Vietnam; ZISP 12811–12833).

[?]Ophryophryne sp. cf. poilani — Poyarkov [Paiarkov] and Vassilieva 2011:202 (Bidoup Mt., Bidoup–Nui Ba N.P., Lam Dong Prov., Vietnam; ZMMU A-4713).

*Ophryophryne synoria* — Vassilieva et al. 2016:54–56, figs. 44–47 (Binh Phuoc Prov. and Dong Nai Prov., Vietnam; ZMMU A-4516, ZMMU A-5003).

##### Holotype.

FMNH 262779, adult male from “O Chung Chry Stream, near 12°17'30"N, 107°03'06"E, 500 m elev., Seima Biodiversity Conservation Area, O’Rang District, Mondolkiri Province, Cambodia”, collected by Bryan Lynn Stuart, Sok Ko and Neang Thy (Stuart et al. 2006; FMNH specimen catalogue). Re-examined by SM.

##### Paratype.

FMNH 262778, adult male, collected with the holotype (Stuart et al. 2006).

##### Measurements of the holotype

**(all in mm, taken by SM).** SVL 48.8; HW 14.2; HL 13.1; IFE 6.1; IBE 10.8; ED 4.7; TYD 3.0; TYE 3.1; SL 4.3; EN 2.5; NS 1.9; IUE 3.5; IN 3.4; UEW 4.1; FAL 12.0; HAL 13.0; FIL 5.1; FIIL 5.1; FIIIL 8.4; FIVL 5.5; SHL 21.8; TL 21.2; FOL 20.0; TFOL 29.4; IMT 2.2.

##### Distribution and remarks.

Stuart et al. (2006) described a large-sized *Ophryophryne* from O’Rang (also spelled as “O’Reang”) District in eastern Cambodia, close to the Vietnamese border, as *O. synoria* (Loc. 1, Fig. 1). Subsequently, during field surveys in 2009–2011, the species was reported in southern Vietnam from Bu Gia Map N.P., Binh Phuoc Prov. (Loc. 2, Fig. 1) and Cat Tien N.P. in Dong Nai Prov. (Loc. 3, Fig. 1) based on morphological evidence (Vassilieva et al. 2016). Herein, we confirm the identity of these specimens based on morphological and molecular genetic evidence, and further expand its distribution in southern Vietnam to include medium and low elevation localities in the central and western parts of the Langbian Plateau (Dak Lak, Lam Dong, Dong Nai and Binh Phuoc provinces between 200 and 1500 m a.s.l.; its presence in Dak Nong Prov. is anticipated). We also identify two mtDNA lineages within *O. synoria* with a moderate level of sequence divergence (*p* = 2.6%: Table 2, Fig. 2): Subclade B inhabits mountain areas in Lam Dong and Dak Lak provinces and was also recorded for the lowland habitat in Dong Nai Prov. (Locs. 3–4, 6 and 11, Fig. 1) whereas Subclade C is only found in Mondolkiri Prov. of Cambodia and adjacent Binh Phuoc Prov. of Vietnam (Fig. 1, Locs 1–2) and corresponds to *O. synoria* *s. stricto.*

##### Variation.

The studied specimens of *O. synoria* showed substantial variation in morphological characters, including SVL (Fig. 3) and other morphometric characters (Fig. 4), coloration, and degree of development of palpebral projection (Fig. 6D, E and F). Overall morphology, coloration, and skin glands of the newly discovered populations of *O. synoria* are in general agreement with the description of the holotype by Stuart et al. (2006). Young specimens from Bidoup–Nui Ba N.P. in life often have reddish or orange coloration of thighs and groin, which was not observed in the type specimens from Cambodia (Stuart et al. 2006), nor in the Bu Gia Map population. The degree of development of short dorsolateral glandular folds varied among specimens, but they were always distinct (Fig. 6D, E and F). The holotype and the Bu Gia Map population (Subclade C) have the finger length formula F1 = F2 < F4 < F3, while in populations from Bidoup–Nui Ba N.P. and Chu Yang Sin N.P. (Subclade B), the finger length formula is F1 = F4 < F2 < F3. Subclade B populations also tend to have a slightly larger tympanum (TYD/ED 68.4%–80.1%; TYE/TD 73.3%–80.6%) than the nominative *O. synoria* (TYD/ED 62.0%–71.9%; TYE/TD 83.9%–103.3%). Though the taxonomic status of the two revealed lineages is not completely clear, herein we tentatively regard them as deep intraspecific mtDNA lineages based on observed genetic differentiation and overall morphological similarity.

##### Vernacular name.

English: “*O’Reang Mountain Toad*” (this paper); “*O’Reang horned frog*” (Vassilieva et al. 2016); Vietnamese: “*Cóc Núi O-Reng*” (Vassilieva et al. 2016).

### Description of a new species of *Ophryophryne*

Based upon several lines of evidence, including the analyses of diagnostic morphological characters, acoustic analyses of advertisement calls and phylogenetic analyses of mtDNA sequences for the 12S rRNA–16S rRNA genes, the new species of *Ophryophryne* from mid to high elevations of the western Langbian Plateau represents a highly divergent mtDNA lineage, clearly distinct from all other *Ophryophryne* species. These results support our hypothesis that this recently discovered lineage of *Ophryophryne* represents an undescribed species, described below:

#### Ophryophryne elfina sp. n.

Taxon classificationAnimaliaAnuraMegophryidae

http://zoobank.org/481B0CFA-5428-40E1-A7A3-C132DC840EC0

Figs 6G, H , 8 , 9 , 10 , 11 , 12 , 13C , 14A (right), 14B, C

##### Chresonymy:

“*Ophryophryne* sp. 1” — Orlov et al. 2008:82 (Chu Yang Sin N.P., Dak Lak Prov., Vietnam; ZISP 12880–12884).

[?] *Ophryophryne gerti* — Stuart et al. 2010:40 (eight uncataloged topotype females from Bidoup–Nui Ba N.P., Langbian Plateau, Vietnam).

“*Ophryophryne* sp.” — Poyarkov [Paiarkov] and Vassilieva 2011:174, 202; fig. 5.6 (Bidoup Mt., Bidoup–Nui Ba N.P., Lam Dong Prov., Vietnam; ZMMU A-4716, ZMMU A-4788, ZMMU A-5674, ZMMU A-5675).

*Ophryophryne gerti* — Nguyen et al. 2014:148–149; fig. 2 (partim––Hon Ba Mt., Hon Ba Nature Reserve [hereafter N.R.], Khanh Hoa Prov., Vietnam; VNMN 983, ZFMK 94220).

##### Holotype.

**Figure 8.**
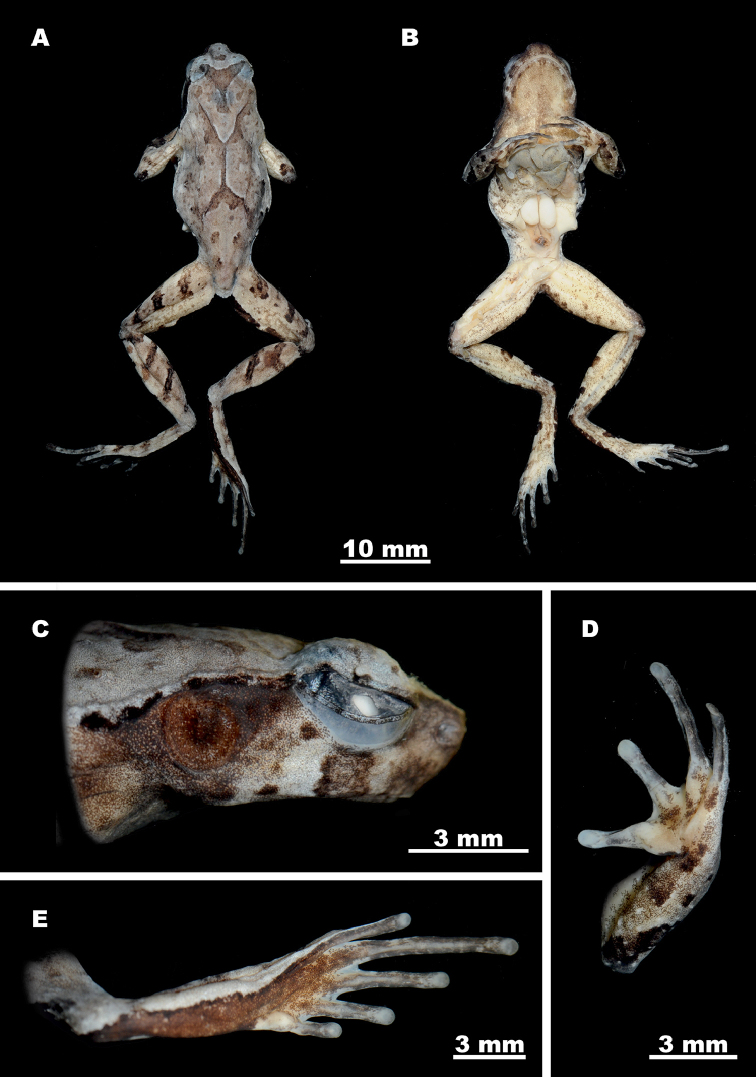
Holotype *Ophryophryne elfina* sp. n. (ZMMU A-5669, male, field number NAP-02658) in preservative: **A** dorsal view **B** ventral view **C** head, lateral view **D** volar view of the left hand **E** plantar view of the left foot. Photos by N.A. Poyarkov.

**Figure 9.**
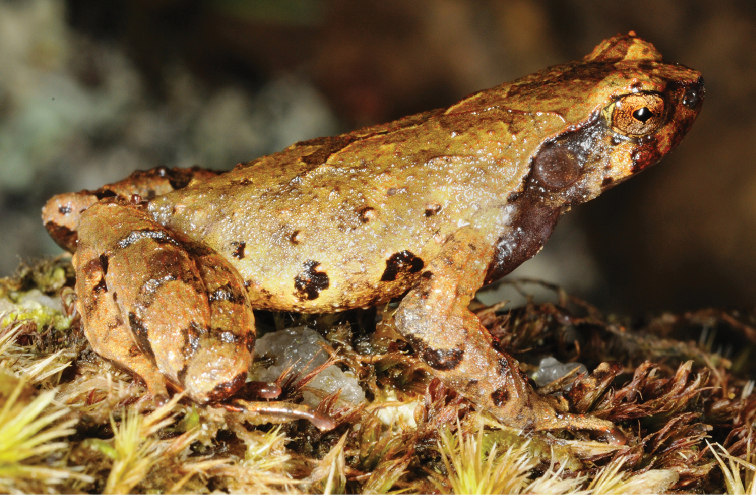
Holotype of *Ophryophryne elfina* sp. n. in life (ZMMU A-5669, male, field number NAP-02658), dorsolateral view. Photos by N.A. Poyarkov.

ZMMU A-5669 (field number NAP-02658), adult male from the northern slope of Chu Pan Fan Mountain, Chu Yang Sin National Park, Bong Krang Commune, Lak District, Dak Lak Province, Vietnam (coordinates 12°22'31.90"N; 108°21'14.10"E, elevation 1725 m a.s.l.), collected along a mountain stream in mixed evergreen montane tropical forest by N.A. Poyarkov on 07 April 2012 (Figs 8 and 9).

##### Paratypes.

ZMMU A-5691 (field numbers ABV-00580; ABV-00581), two juveniles from the north-western slope of Chu Yang Sin Mountain, Chu Yang Sin N.P., Hoa Le Commune, Krong Bong Dist., Dak Lak Prov., Vietnam (12°24'47.70"N; 108°24'25.40"E, 1975 m a.s.l.), collected in leaf litter in mixed evergreen montane tropical forest by N.A. Poyarkov on 25 May 2014; ZMMU A-5692 (field number NAP-00582), adult male from the northern slope of Bidoup Mountain, Bidoup–Nui Ba N.P., Da Chais Commune, Lac Duong Dist., Lam Dong Prov., Vietnam (12°06'5.60"N; 108°39'34.20"E, 2035 m a.s.l.), collected along mountain stream in evergreen montane elfin forest by N.A. Poyarkov on 09 May 2009; ZMMU A-5675 (field numbers NAP-01456; NAP-01459), two adult males from the northern slope of Bidoup Mountain, Bidoup–Nui Ba N.P., Da Chais Commune, Lac Duong Dist., Lam Dong Prov., Vietnam (12°06'5.60"N; 108°39'34.20"E, 2035 m a.s.l.), collected on stones and vegetation along a mountain stream in mixed evergreen montane elfin forest by N.A. Poyarkov and A.B. Vassilieva on 25 June 2010; ZMMU A-4788 (field numbers NAP-01455; NAP-01449; NAP-01450; NAP-01460), four adult males from the southern slope of Hon Giao Mountain Ridge, Bidoup–Nui Ba N.P., Da Chais Commune, Lac Duong Dist., border of Lam Dong Prov. and Khanh Hoa Prov., Vietnam (12°11'33.10"N; 108°42'41.80"E, 1890 m a.s.l.), collected along a mountain stream sitting on stones and tree branches near the water edge, in mixed evergreen montane elfin forest by N.A. Poyarkov and A.B. Vassilieva on 30 June 2010; ZMMU A-5674 (field numbers NAP-01451; NAP-01452), two adult males from the southern slope of Hon Giao Mountain Ridge, Bidoup–Nui Ba N.P., Da Chais Commune, Lac Duong Dist., border of Lam Dong Prov. and Khanh Hoa Prov., Vietnam (12°11'33.10"N; 108°42'41.80"E, 1890 m a.s.l.), collected along a mountain stream on stones and vegetation in evergreen montane elfin forest by N.A. Poyarkov and A.B. Vassilieva on 29 June 2010; ZMMU A-5170 three adult males (field numbers ABV-00454; ABV-00472; ABV-00471), and one adult female (ABV-00455) from the northern slope of Bidoup Mountain, Bidoup–Nui Ba N.P., Da Chais Commune, Lac Duong Dist., Lam Dong Prov., Vietnam (12°06'5.60"N; 108°39'34.20"E, 2035 m a.s.l.), collected along a mountain stream in mixed evergreen montane tropical forest by A.B. Vassilieva on 16 April 2014.

**Referred specimens.** ITBCZ 2786, ITBCZ 2788, ITBCZ 2792, ITBCZ 2828, three adult females and one adult male collected along a mountain stream in evergreen mountain forest on the summit of Hon Ba Mountain, Hon Ba N.R., Dien Khanh Dist., Khanh Hoa Prov., Vietnam (12°07'10.60"N; 108°56'51.60"E, 1510 m a.s.l.), by Sang Ngoc Nguyen, Luan Thanh Nguyen and Vu Dang Hoang Nguyen on 22–24 December 2015; ITBCZ 2908–2909, ITBCZ 2918–2919, ITBCZ 3502, five adult males collected along a mountain stream in evergreen montane forest on the summit of Hon Ba Mountain, Hon Ba N.R., Dien Khanh Dist., Khanh Hoa Prov., Vietnam (12°07'28.80"N; 108°58'14.20"E, 950 m a.s.l.), by Sang Ngoc Nguyen, Luan Thanh Nguyen, and Vu Dang Hoang Nguyen on 22–28 March 2016; ZMMU A-5679 (field number NAP-01169), 7 larvae collected in a cascade mountain stream on the northern slope of Bidoup Mountain, Bidoup–Nui Ba N.P., Da Chais Commune, Lac Duong Dist., Lam Dong Prov., Vietnam (12°06'5.60"N; 108°39'34.20"E, 2035 m a.s.l.), by N.A. Poyarkov on 03 May 2009; ZMMU A-5684 (field number NAP-02673), 4 larvae collected in a cascade mountain stream on the northern slope of Chu Pan Fan Mt., Chu Yang Sin N.P., Bong Krang Commune, Lak Dist., Dak Lak Prov., Vietnam (coordinates 12°22'31.90"N; 108°21'14.10"E, elevation 1725 m a.s.l.), by N.A. Poyarkov on 07 April 2012.

##### Etymology.

The specific epithet is an adjective (in agreement with the genus name in feminine gender), derived from “*elf*”, the English spelling of “*alfus*” in Latin, referring to usually forest-dwelling supernatural mythological creatures in Germanic mythology and folklore; the name is given in reference both to the funny appearance and small size of the new species, as well as to the their endangered habitat, restricted to wet evergreen montane forests at high elevations of the Langbian Plateau; such forests are often called “*elfin forests*”.

##### Recommended vernacular name.

The recommended common name in English is “Elfin Mountain Toad”. The recommended common name in Vietnamese is “Cóc Núi Tiểu Yêu Tinh”.

##### Diagnosis.

The species is allocated to *Ophryophryne* based on its obvious similarities with its sister taxa, its molecular phylogenetic affinities, and the absence of maxillary teeth considered diagnostic for the genus (previous authors, e.g. Ohler 2003, Delorme et al. 2006 and Fei et al. 2009 also indicated a horizontal pupil and the absence of vomerine teeth as diagnostic for *Ophryophryne*, this is reconsidered by Mahony et al. 2017). *Ophryophryne elfina* sp. n. is distinguished from its congeners by a combination of the following morphological attributes: (1) small adult body size, male SVL 26.9–33.9 mm (*N* = 29), female SVL 35.1–36.5 mm (*N* = 6); (2) snout sharply protruding in profile; (3) tympanum diameter approximately half of eye diameter; tympanum to eye distance approximately 70–90% of tympanum diameter; (4) finger length formula: F1 < F4 ≤ F2 < F3, or F1 ≤ F2 < F4 < F3; toe webbing rudimentary, toe length formula: T1 < T5 < T2 < T3 < T4; (5) short dorsolateral glandular ridge present above shoulder; (6) palpebral projection present as a small single tubercle to moderately developed single projection; (7) dermal cloacal protuberance and dermal flaps above cloacal opening absent; (8) skin of dorsal and lateral surfaces of head, body and limbs shagreened with numerous small tubercles, large warts on the flanks; (9) skin on dorsal and lateral surfaces of body, head and limbs with numerous bright orange-red (in life) asperities; (10) males with a red-orange (in life) nuptial pad on F1; (11) dorsal coloration light yellow-brown with dark hourglass-shaped marking on dorsum usually edged with white or beige (in life); (12) posterior suborbital light bar well-defined, usually clearly separated from dark-brown temporal triangular spot, uniformly covering temporal area and tympanum.

The new species is also markedly distinct from all congeners for which comparable sequences are available (16S rRNA mitochondrial gene; uncorrected genetic distance > 8.2%). The advertisement call of the new species consists of whistling notes uttered in series: average 12.84 ± 0.41 calls per series, with an average dominant frequency of 4645.94 ± 4.39 Hz, repetition rate per recording/series 1.18 ± 0.2 calls/s and 3.87 ± 0.07 calls/s, respectively, with average call duration 73 ± 0.23 ms and inter-call interval 207 ± 2.06 ms, also distinguishes the new species from *Ophryophryne* species for which calls are known, including the two species found in sympatry.

##### Description of holotype.

Mature male (SVL 27.2 mm); habitus slender (Figs 8A, B, and 9). Specimen in good state of preservation; median abdomen dissected, dissection length ca. 9.0 mm; liver and testes observable through incision, testes white, enlarged (testes length 3.9 mm; Fig. 8B); ventral right femur dissected for molecular sampling, dissection length 9.8 mm.

Head moderately large (HL/SVL 29.2%; HW/SVL 29.5%), almost as wide as long (HW/HL 101.0%), triangular in dorsal view; top of head flat; snout comparatively short (ES/HL 30.9%), narrow (IFE/HW 39.4%), somewhat truncated in dorsal view (Fig. 8A), sharply protruding in profile, without rostral appendage (Figs 8C, 9); snout projecting significantly beyond margin of lower jaw (Fig. 8C); loreal region slightly concave; canthus rostralis distinct, sharp; dorsal region of snout flat; eyes large (ED/HL 44.8%), slightly protuberant in dorsal view and in profile, eye less than twice as long as maximum tympanum diameter (TYD/ED 53.7%) and half times longer than snout (ED/SL 145.3%); eye-tympanum distance less than maximum tympanum diameter (TYE/TD 83.3%); tympanum distinct, almost circular shaped with vertical diameter slightly exceeding horizontal diameter, tympanum large (TYD/HL 24.1%); pupil in preservation diamond-shaped (Fig. 8C), horizontally orientated; nostril oval-shaped, vertical, laterally orientated, located as far from eye as from snout (EN/NS 100.8%); internarial distance greater than eyelid width (IN/UEW 119.5%), and subequal to narrowest point between upper eyelids (IN/IUE 103.7%); pineal ocellus not visible externally (Fig. 8A); vomerine ridges not absent; maxillary and vomerine teeth absent; vocal sac gular, its openings not discernable; tongue moderately large, with free posterior end, not notched posteriorly, lacking medial lingual process.

Forelimbs slender, forearm moderately long (FAL/SVL 25.7%), slightly enlarged relative to upper forelimb, and shorter than hand (FAL/HAL 96.1%); fingers long and narrow, dorsoventrally flattened, weak lateral fringes present on third and fourth fingers (Fig. 8D), finger length formula F1 < F4 < F2 < F3; fingers completely free of webbing; subarticular tubercles absent, replaced by low callous dermal ridges; supernumerary tubercles absent; outer palmar (metacarpal) tubercle small, round, elevated but with indistinct borders (Fig. 8D); thenar tubercle weak; finger tips in life rounded, weakly expanded relative to digit widths (wider than the distal-most finger articulation), with circular pads (Fig. 8D); terminal grooves absent.

Hindlimbs slender, relatively long, shanks overlap when thighs are held at right angle to body; shank length less than half of snout–vent length (SHL/SVL 48.7%); thighs shorter than shanks (SHL/TL 109.2%), and feet (FOL/TL 110.9%); toes long and slightly dorsoventrally flattened (Fig. 8E), relative toe lengths T1 < T5 < T2 < T3 < T4; toe tips slightly expanded relative to digit widths (wider than the distal-most toe articulation), with circular pads; terminal grooves absent; lateral fringes on toes, outer metatarsal tubercle, subarticular and supernumerary tubercles absent; inner metatarsal tubercle well developed with distinct borders, ca. two times longer than wide, oval-shaped (IMT/FOL 9.3%); well-developed dermal ridge of callous tissue present on ventral surface of all toes and continuing to metatarsus; rudimentary webbing present between all five toes, basal web distinct between toes T2–T3 and T3–T4, but is completely reduced between toes T1–T2 and T4–T5; tarsal fold absent.

##### Skin texture and skin glands.

Skin of dorsal and lateral surfaces of head, body and limbs shagreened, with numerous small skin asperities present on anterior two thirds of dorsum, sparse posteriorly, increasing in density along dermal ridges, densely covering dorsal and lateral surfaces of head, upper eyelids, and dorsal surfaces of thighs, shanks, upper forelimbs, forearms, hands, feet and digits, and absent from all remaining surfaces. Small tubercles finely and relatively evenly scattered on dorsal surfaces of trunk, head and limbs, including maxilla, mandible, eyelids and dorsal surfaces of head, forelimbs and hindlimbs (Figs 8A and 9); small tubercles present on temporal region, tympanum smooth, tympanic rim distinct but not elevated relative to skin of temporal region; on dorsal surfaces tubercles arranged in distinct longitudinal ridges on upper forelimbs, forearms, shanks and thighs, becoming less distinct on dorsal surfaces of hands, feet and digits (Fig. 9); six large tubercles on left flank and seven large tubercles on right flank irregularly scattered from axilla to groin, intermixed with smaller tubercles; central portion of outer edge of upper eyelid slightly thickened, with a distinct small single tubercle on a thickened ridge (Figs 8C, 9); distinct thick glandular supratympanic fold, narrow anteriorly, considerably widening posteriorly, extending from posterior corner of eye gently sloping down towards dorsal margin of tympanum (but not concealing it), where it broadly curves down, terminating above axilla (Figs 8C, 9); short dorsolateral glandular ridge present above shoulders, on anterior part of dorsum, its length comparable with eye diameter (Fig. 9); a weak “ >–< ”-shaped glandular dermal parietoscapular-sacral ridge present on dorsum (Figs 8A, 9); transverse fold at head basis absent; dermal projection above cloaca absent; gular region, chest, abdomen and ventral surfaces of limbs smooth to weakly shagreened (especially on the posterior surface of abdomen); nuptial pad present, single, covered with microgranules, covering entire dorsal metacarpal of first finger extending distally to ca. 3/4 basal phalange length; pectoral glands round, flat, of medium size, positioned level with axilla; femoral gland flat, indistinct, on posterior surface of thighs.

##### Color of holotype in life.

Entire dorsum light olive-brown to yellow-brown with large irregular brownish grey spots; dorsal surfaces of head yellowish brown from tip of snout to eyes; small oval-shaped spot with irregular borders on dorsal surface of snout between anterior canthi; a small dark dot on dorsal surface of snout tip; similar single dark dots on anterior parts of upper eyelids; brown “V”-shaped marking on crown between supraorbital horns with apex pointing posteriorly, outlined with thin light-beige edging; round brownish spot at head basis; “ >–< ”-shaped marking surrounded with dark olive-brown, outlined with thin light-beige edging forming a hourglass-shaped dorsal marking (Fig. 8A); two small roundish brown spots at sacrum (Fig. 8A); supratympanic fold dorsally light yellowish brown, ventrally dark-brown; front and lateral surfaces of snout and lateral canthus rostralis dark reddish brown; lateral surfaces of maxilla dark brown with four distinct orange-brown to yellowish beige bars extending from orbits towards edge of maxilla: smallest anteriormost light band borders nostril ventrally, with two posterior light bands extend from posterior corner of eye towards angle of mouth (Fig. 9); axilla purplish brown; tympanum uniform purplish brown; temporal region uniform dark purplish brown, clearly defined from light beige area on posterior part of maxilla; pupil black, outlined in copper-gold; iris golden dorsally and ventrally, copper-orange at medial part, with tiny dark reticulations spreading from pupil; sclera lemon-yellow; upper surface of limbs yellowish brown with irregular dark-brown spots on forearms and transverse spots forming dark-brown and greyish bands across shanks, thighs and tibio-tarsus (three complete transverse bands on left leg, two complete [both on shank and thigh] and one incomplete [on shank only] transverse bands on right leg: Figs 8 and 9), knee joint dark brown; sides beige-yellow with indistinct greyish white flecking and large black spots with irregular borders: marking location of large warts on each side of body, four smaller spots located dorsally, and two large brown-black spots located ventrally, top of larger flank tubercles brownish cream (Fig. 9); throat brownish to purplish grey with greyish white flecking and irregular dark-brown spots; chest and anterior half of abdomen purplish grey with whitish flecking and grey-brown blotches; posterior half of abdomen lighter greyish pink with irregular dark blotches; lower surface of limbs purplish grey with white and beige flecking; area surrounding vent and posterior surface of thighs dark black-brown with whitish spots, posterior surface of thighs near tibio-tarsal articulation black-brown with sparse whitish dots; dorsal surface of feet and shanks yellowish beige with brown flecking; ventral surface of feet and shanks brown-black. Pectoral and femoral glands creamy white. Nuptial pad, outer metacarpal (palmar) and metatarsal tubercles pink-red to orange-red. Asperities covering dorsal surfaces of body, head, limbs and digits, lateral sides of head and anterior part of chest in life bright orange-red, forming reddish rows and ridges on dorsal surfaces of limbs as well as on edge of upper eyelid, palpebral projection also with orange-red asperities.

##### Color of holotype in preservative.

In preservative coloration faded to light grey-brown on dorsum and flanks, with slightly paler limbs and greyish beige to whitish on venter; reddish and orange tints, as well as iris coloration, faded completely; dark markings on dorsum, sides and venter and other features remain without significant change (Fig. 8). Banding on limbs is less pronounced. Chest, abdomen, throat, interior portions of forelimbs and thighs are pale greyish brown; formerly brightly colored dorsal asperities and nuptial pads, palmar and metatarsal tubercles turned transparent or creamy white (Fig. 8C, E).

##### Measurements of the holotype

**(all in mm, taken by NAP).** SVL 27.2; HW 8.0; HL 7.9; IFE 3.2; IBE 6.8; ED 3.6; TYD 1.9; TYE 1.6; SL 2.5; EN 1.3; NS 1.3; IUE 2.3; IN 2.3; UEW 2.0; FAL 7.0; HAL 7.3; FIL 2.4; FIIL 2.8; FIIIL 4.6; FIVL 2.8; SHL 13.2; TL 12.1; FOL 11.9; TFOL 18.9; IMT 1.8; TIL 1.7; TIIL 3.8; TIIIL 5.5; TIVL 6.7; TVL 3.5.

##### Variation.

**Figure 10.**
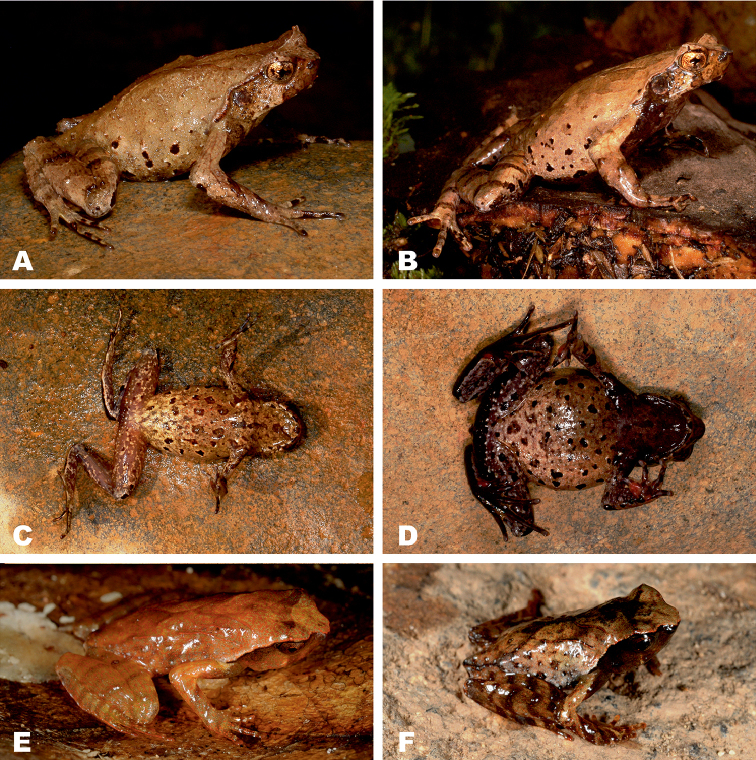
Paratypes of *Ophryophryne elfina* sp. n. in life. **A–D** Bidoup Mt., Bidoup–Nui Ba N.P., Lam Dong Prov., 2000 m a.s.l.: **A** ZMMU A-4788 (field number NAP-01449), male, dorsolateral view **B** ZMMU A-4788 (field number NAP-01455), female, dorsolateral view **C** ZMMU A-4788 (field number NAP-01449), male, ventral view **D** ZMMU A-4788 (field number NAP-01455), female, ventral view **E–F** Chu Yang Sin Mt., Chu Yang Sin N.P., Dak Lak Prov., 1800 m a.s.l.: **E** ZMMU A-5691 (field number ABV-00580), metamorph, dorsolateral view **F** ZMMU A-5691 (field number ABV-00581), metamorph, dorsolateral view. Photos by N.A. Poyarkov.

Morphometric variation within the type series and other referred specimens of the new species is shown in Table 5. Individuals of the type series are similar in morphology and body proportions (Figs 9, 10). There is a clear and significant difference in body size between males and females (Fig. 3): females (SVL 35.1–36.5 mm, *N* = 6) are significantly bigger than males (SVL 26.9–33.9 mm, *N* = 29) (Duncan test, p < 0.05); sexual differences were not significant for other mensural characters possibly due to the small sample size of females. Certain variation is observed in finger lengths: most of the examined specimens have the finger length formula F1 < F4 < F2 < F3 (*N* = 14), in some specimens, including the holotype, the second and the fourth fingers are of equal length (F1 < F4 = F2 < F3; *N* = 5), or the fourth finger is longer than the second (F1 < F2 < F4 < F3; *N* = 5); rarely the second finger is as long as the first finger (F1 = F2 < F4 < F3; *N* = 2). Specimens vary in the number and size of black spots and blotches on flanks (Fig. 10A, B). In life, both sexes of the new species have lighter dorsum and belly coloration when nocturnally active. Other in-life variation was observed for throat coloration: throat can be dark brownish with clear dark-grey blotches (Fig. 10C) to almost uniform brown-violet to purple with dark blotches not discernable (Fig. 10D). There is significant variation in dorsal pattern: in some specimens the dorsum looks almost uniform yellowish brown with an indistinct hourglass-shaped figure (Figs 6G, 10A, B, 14 [right]) whereas in other specimens the hourglass-shaped figure is distinct, dark brown and edged with light beige (Figs 6H, 9). There is some variation in the length of palpebral projections, from a small almost indistinct tubercle (Figs 6H, 13C) to a moderately well-developed projection (Figs 6G, 14). Coloration of the lateral surfaces of the head vary, but on all specimens two light suborbital bars are distinct, clearly separated from the uniform dark-brown coloration of the tympanal area. Iris coloration shows insignificant variation: Nui Chua Mt. population appear to have copper-red coloration of the entire iris “(Fig. 14B), somewhat different from the coloration of the holotype (Fig. 9). Recently metamorphosed and juvenile specimens have numerous bright red-orange tubercles (Fig. 10E, F) which are more conspicuous than in adults. Excluding the presence of nuptial pads on males, the new species shows no significant variation in dermal characters among sexes (Fig. 10); in preservative smaller tubercles become flattened and less distinct.

**Table 5.** Measurements of the *Ophryophryne elfina* sp. n. specimens. For museum accession numbers relative to specimen collection numbers (NAP, ABV, HB, ROM, CYS) see Appendix 1. For locality details relative to population number see Table 1 and Fig. 1. For abbreviations see Material and methods. All measurements are given in mm. Table is continued on the next two pages.

Specimen ID	Population	Sex	Type status	FAL	HAL	FIL	FIIL	FIIIL	FIVL	SHL	TL	FOL	TFOL	IMT	IMT
NAP-02658	10	m	holotype	27.2	8.0	7.9	3.6	1.9	1.6	2.5	1.3	1.3	2.3	2.3	2.0
NAP-01455	6	m	paratype	33.9	10.1	9.8	3.8	2.3	1.9	2.7	1.3	1.3	2.8	3.1	2.9
NAP-01449	6	m	paratype	31.0	9.3	9.8	3.8	2.2	1.6	2.5	1.3	1.2	3.1	2.9	3.5
NAP-01450	6	m	paratype	29.2	9.0	9.0	3.7	2.3	1.4	3.0	1.2	1.4	3.2	2.5	3.2
NAP-01460	6	m	paratype	29.6	8.7	8.4	3.4	2.7	1.1	2.1	1.1	1.0	2.5	1.5	2.6
ABV-00316	8	m		28.9	8.8	9.1	3.8	2.6	1.6	2.2	1.3	1.1	2.8	1.8	3.1
HB-36-1	8	m		27.5	8.6	9.2	4.1	2.4	1.3	2.6	1.4	1.4	2.6	2.1	2.6
ROM-36525	11	m		26.9	7.6	8.1	3.6	1.9	1.3	2.9	1.3	1.2	2.3	1.7	2.8
ROM-36523	11	m		27.8	8.2	8.4	4.0	2.2	1.8	2.8	0.9	1.5	2.1	1.6	2.9
ROM-36529	11	m		28.3	7.9	8.6	4.3	2.2	1.6	2.4	1.1	1.5	2.8	2.0	2.7
NAP-01757	6	m		32.0	9.2	9.2	4.0	2.3	1.8	2.6	1.3	1.3	2.3	2.3	2.5
NAP-01782	6	m		31.1	9.3	9.1	3.8	2.2	1.7	2.3	1.1	1.0	2.8	2.6	7.9
NAP-01758	7	m		32.1	9.2	9.3	3.4	2.4	1.7	2.4	0.8	1.3	3.1	2.0	2.4
NAP-01871	7	m		32.8	8.8	9.6	3.7	2.2	1.3	2.7	0.9	1.1	2.9	1.6	2.4
NAP-01783	7	m		31.3	9.0	9.2	4.0	2.2	1.6	2.8	1.2	1.1	3.1	2.1	2.8
ABV-00471	6	m	paratype	30.7	8.5	8.5	3.7	2.3	1.3	2.6	0.9	1.4	2.7	2.4	2.7
ABV-00454	6	m	paratype	31.4	8.8	9.3	3.8	2.1	1.5	2.0	1.0	1.1	2.5	1.9	2.9
ABV-00472	6	m	paratype	30.8	8.9	9.3	3.3	2.3	2.1	2.3	1.6	1.0	3.2	2.1	2.6
ROM-36524	11	m		28.5	8.3	8.5	3.6	2.1	1.2	2.8	0.8	1.6	2.4	2.2	2.5
ROM-36527	11	m		27.7	7.4	7.8	3.1	2.0	1.7	3.0	1.3	1.8	2.7	2.1	2.7
ROM-36522	11	m		28.1	7.9	8.4	3.2	1.7	1.3	2.8	1.2	1.5	2.6	2.1	2.8
ROM-36528	11	m		28.2	7.2	7.4	3.1	1.6	1.5	3.3	1.0	1.8	3.4	1.9	2.9
ROM-36526	11	m		27.1	7.6	8.2	3.6	2.1	1.5	2.9	1.1	2.1	2.6	2.1	2.4
ITBCZ 2828	8	m		27.9	8.2	8.9	3.9	2.2	1.2	2.5	1.1	1.4	2.1	2.8	3.1
ITBCZ 2908	8	m		29.4	9.6	9.1	3.4	2.3	1.4	3.3	1.1	2.2	2.7	3.3	2.7
ITBCZ 2909	8	m		30.1	9.5	9.8	4.1	2.2	1.8	2.9	1.3	1.6	2.4	3.3	2.7
ITBCZ 2918	8	m		29.6	8.7	9.3	3.4	2.2	1.3	2.8	1.1	1.7	2.2	2.9	2.7
ITBCZ 2919	8	m		30.8	9.2	9.6	3.4	2.1	1.6	3.3	1.4	1.9	2.4	2.6	2.6
ITBCZ 3502	8	m		28.6	8.7	8.9	3.5	2.3	1.8	3.3	1.2	2.1	2.6	2.8	2.5
ABV-00455	6	f	paratype	35.2	9.3	8.9	4.2	2.4	1.7	2.4	1.7	1.2	2.8	1.7	2.6
CYS-10-10	11	f		35.1	9.4	9.1	3.9	1.9	1.9	3.6	1.6	2.1	3.5	2.6	3.0
ROM-36530	11	f		35.7	9.0	8.6	3.6	2.1	1.6	3.3	1.0	1.9	3.6	2.4	2.8
ITBCZ 2786	8	f		36.5	10.0	11.0	4.1	2.4	1.8	2.7	1.2	1.5	2.7	2.8	3.1
ITBCZ 2788	8	f		35.8	10.1	10.9	4.2	3.0	1.6	3.2	1.3	1.9	2.3	2.6	2.8
ITBCZ 2792	8	f		35.3	10.2	10.3	4.1	2.2	1.8	3.3	1.5	1.8	2.8	2.9	3.1
**Specimen ID**	**Population**	**Sex**	**Type status**	**FAL**	**HAL**	**FIL**	**FIIL**	**FIIIL**	**FIVL**	**SHL**	**TL**	**FOL**	**TFOL**	**IMT**	
NAP-02658	10	m	holotype	7.0	7.3	2.4	2.8	4.6	2.8	13.2	12.1	18.9	11.9	1.8	
NAP-01455	6	m	paratype	8.5	9.4	2.3	4.1	6.0	3.7	16.1	14.8	14.7	28.0	2.0	
NAP-01449	6	m	paratype	7.1	7.5	2.8	3.0	4.1	3.0	14.4	13.4	11.8	19.4	1.7	
NAP-01450	6	m	paratype	7.5	7.8	2.1	2.9	4.9	2.6	12.9	13.8	11.1	19.7	1.9	
NAP-01460	6	m	paratype	6.5	6.7	2.3	2.9	4.4	2.4	13.4	11.3	10.6	17.5	1.3	
ABV-00316	8	m		7.3	6.7	2.4	2.9	4.9	2.9	13.2	13.5	11.6	19.2	2.5	
HB-36-1	8	m		6.6	7.1	2.1	3.2	4.6	3.0	12.6	13.4	11.6	18.9	1.5	
ROM-36525	11	m		6.9	6.6	2.0	2.4	4.5	2.7	11.7	11.9	10.6	17.1	1.2	
ROM-36523	11	m		6.4	6.0	2.9	2.9	4.8	3.0	12.8	13.2	11.4	18.3	1.6	
ROM-36529	11	m		6.7	6.4	2.9	3.0	4.5	3.0	13.5	13.5	11.0	19.1	1.6	
NAP-01757	6	m		8.4	7.4	2.5	2.5	4.5	3.1	13.3	14.5	11.6	19.6	1.2	
NAP-01782	6	m		6.1	4.8	1.1	2.0	3.7	1.4	12.7	13.1	11.0	18.7	1.4	
NAP-01758	7	m		8.1	6.6	2.1	3.2	4.6	2.5	14.8	15.3	13.3	20.4	1.8	
NAP-01871	7	m		6.5	6.8	2.3	2.8	4.6	3.1	14.2	14.6	10.8	19.2	1.2	
NAP-01783	7	m		7.6	6.1	2.4	3.4	4.8	3.0	14.0	15.7	10.4	18.1	1.6	
ABV-00471	6	m	paratype	8.6	7.1	3.0	3.4	4.9	3.2	14.2	14.5	13.1	19.6	1.2	
ABV-00454	6	m	paratype	8.2	7.2	3.0	3.2	5.1	3.2	15.7	15.3	13.1	22.0	1.3	
ABV-00472	6	m	paratype	7.9	6.5	2.5	3.0	5.2	3.1	14.7	11.9	12.6	21.4	1.2	
ROM-36524	11	m		6.8	6.4	2.2	2.9	4.5	2.4	13.0	13.0	10.8	17.4	1.5	
ROM-36527	11	m		6.4	6.7	2.4	2.6	4.9	2.5	12.2	11.9	11.9	19.0	1.5	
ROM-36522	11	m		6.8	6.6	2.2	2.7	4.3	2.3	12.3	12.9	11.3	18.7	1.2	
ROM-36528	11	m		6.1	5.8	2.2	2.4	4.2	2.5	12.5	13.0	11.7	18.4	1.7	
ROM-36526	11	m		6.1	6.3	2.3	2.5	4.3	2.7	12.7	12.4	12.7	18.2	2.2	
ITBCZ 2828	8	m		7.8	7.1	3.2	2.9	4.1	3.4	4.6	12.0	11.2	19.6	1.8	
ITBCZ 2908	8	m		7.5	8.0	3.5	2.8	5.1	3.9	14.8	14.9	12.7	22.8	1.9	
ITBCZ 2909	8	m		7.8	8.2	3.1	3.8	5.2	4.1	16.3	16.1	12.6	21.9	2.1	
ITBCZ 2918	8	m		7.0	7.3	3.4	3.3	5.0	3.5	19.6	13.4	12.1	20.0	1.7	
ITBCZ 2919	8	m		8.9	8.3	3.4	3.0	6.0	3.6	16.0	15.1	12.3	21.9	2.1	
ITBCZ 3502	8	m		7.8	7.7	3.2	3.0	5.5	3.5	15.0	14.7	12.6	20.7	1.8	
ABV-00455	6	f	paratype	8.3	7.7	2.9	3.3	4.9	3.1	16.1	16.2	14.2	22.6	1.7	
CYS-10-10	11	f		7.9	8.6	3.0	3.4	5.2	3.2	15.3	15.5	13.9	21.8	2.4	
ROM-36530	11	f		7.7	8.7	3.3	4.7	5.6	3.6	14.9	15.8	15.1	23.3	1.5	
ITBCZ 2786	8	f		9.8	9.2	4.1	3.9	6.3	4.6	19.8	17.8	15.7	27.2	2.3	
ITBCZ 2788	8	f		10.1	9.7	4.2	4.1	7.0	4.9	17.7	15.5	15.3	25.0	2.0	
ITBCZ 2792	8	f		10.1	9.1	4.5	4.1	5.8	5.2	19.0	17.2	15.1	26.0	2.1	

##### Tadpole description.

Tadpoles were allocated to *Ophryophryne elfina* sp. n. based on the following evidence: (1) morphological features characteristic for megophryine larvae in general; *Ophryophryne* or *Megophrys* *s. lato* in particular (elliptical shaped body with long muscular tail, oral disk forms a dorsally oriented funnel); (2) collected in the stream where calling males of the new species were recorded; (3) species identification confirmed by mtDNA sequences of short 16S rRNA gene fragment (up to 500 bp) (GenBank Acession numbers: KY515232–KY515233, see Table 1).

Standard tadpoles measurements (mean ± SD, *N* = 5, Stage 25; ZMMU A-5679, field number NAP-01169, collected from Bidoup Mt., 1900 m a.s.l., Bidoup–Nui Ba N.P., Lam Dong Prov.) (all in mm, taken by NAP): TOL = 28.4 ± 1.3 (27.4–30.2); BL = 8.6 ± 0.1 (8.4–8.7); TAL = 19.8 ± 1.2 (18.9–21.5); BW = 4.4 ± 0.4 (3.8–4.6); BH = 3.6 ± 0.2 (3.4–3.7); TH = 4.5 ± 0.4 (4.0–4.8); SVL = 9.2 ± 0.3 (9.0–9.5); SSp = 4.8 ± 0.2 (4.5–4.9); UF = 1.4 ± 0.1 (1.3–1.5); LF = 1.1 ± 0.0 (1.1–1.1); IN = 2.6 ± 0.1 (2.5–2.6); IP = 2.8 ± 0.2 (2.7–3.2); RN = 1.7 ± 0.1 (1.7–1.8); NP = 0.8 ± 0.1 (0.7–0.8); ED = 0.8 ± 0.0 (0.8–0.9).

**Figure 11.**
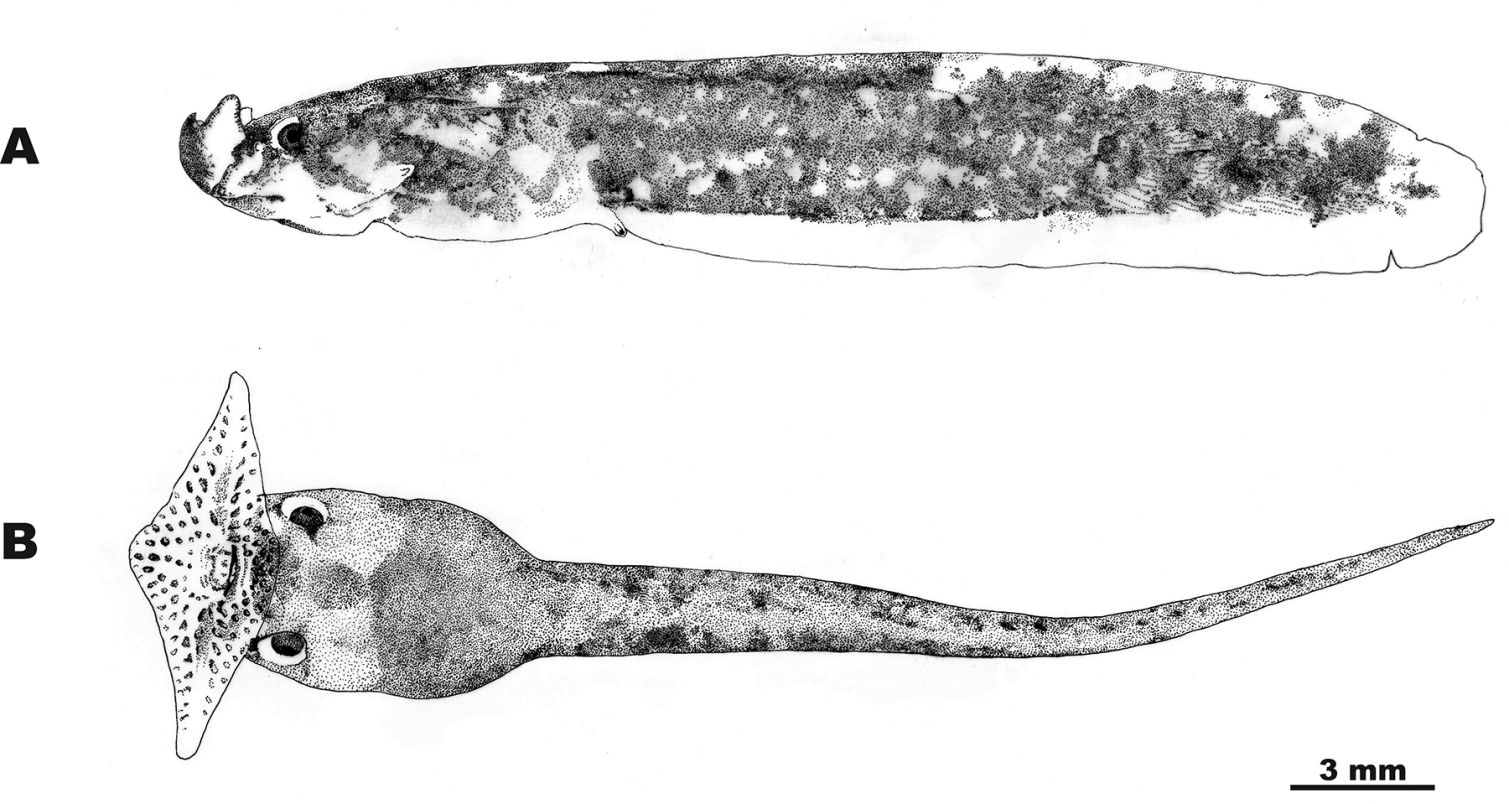
External morphology of the tadpole of *Ophryophryne elfina* sp. n. (ZMMU A-5679, field number NAP-01169; Stage 25, TOL = 30.2 mm). **A** lateral view, mouth funnel closed (in preservative) **B** dorsal view, mouth funnel open (in life). Drawings by V.D. Kretova.

**Figure 12.**
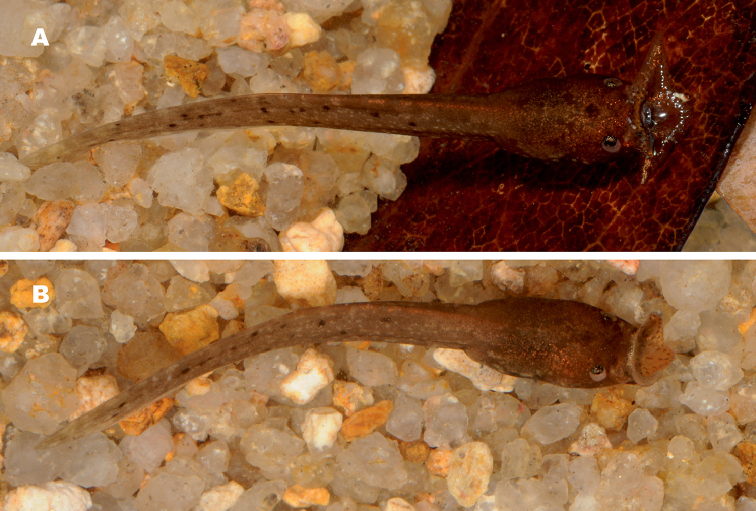
Coloration of *Ophryophryne* tadpoles in life: **A** *Ophryophryne elfina* sp. n. “(ZMMU A-5684, field number NAP-02673; Stage 25) from Chu Pan Fan Mt., 1900 m a.s.l. in Chu Yang Sin N.P., Dak Lak Prov., mouth funnel open **B** tadpole from the same locality and collection data (ZMMU A-5684, field number NAP-02673; Stage 25) with mouth funnel closed.. Photos by N.A. Poyarkov.

The following description is based on five tadpoles at stage 25 (ZMMU A-5679, field number NAP-01169). In lateral view (Fig. 11A), body slightly compressed dorsoventrally (BH/BW 83.5 ± 4.09%), especially anteriorly, convex both dorsally and ventrally. Body elliptical in dorsal view (Fig. 11B), with maximum width at middle of body (BW/BL 51.0 ± 4.2%); snout short, rounded, blunt. Eyes of moderate size (ED/BL 9.8 ± 0.3%), not bulging, separated by a distance which equals approximately 1.1 times internarial distance (IP/IN 110.2 ± 6.8%), directed and positioned dorsolaterally, not visible in ventral view; pupils oriented dorsolaterally. Nares tubular, positioned dorsally (near anterior edge of eye), much closer to pupils than to tip of snout; directed laterally. Spiracle sinistral, conical, very short, opening at half of distance from snout tip to vent (SSp/SVL 52.2 ± 3.4%); spiracle attached to body wall for most of its length, extremity is free, positioned at the level of longitudinal axis, oriented dorsoposteriorly, opening varies from rounded to oval. Tail long, more than two times longer than body (TAL/BL 231.3 ± 11.0%), lanceolate; almost equal in height along its length (point of maximum height of tail located just anterior to midlength of tail); tail tip bluntly rounded, without terminal filament; tail musculature strong, gradually tapering, almost reaching tail tip. Tail fins shallow, moderately well developed, not extending onto body: dorsal fin originating almost at body-tail junction, much shorter than lower fin proximally and nearly equal in height to it on middle of tail; dorsal fin slightly higher than ventral fin on distal half of tail (LF/UF 77.1 ± 4.9%); free margin of dorsal fin horizontal and shallow on anterior half of tail; free margin of ventral fin parallel to tail musculature. Vent opening medial, tubular, directed posteriorly, not linked to ventral tail fin. Neither skin glands nor neuromasts visible in preservative, but neuromasts of the lateral line system are distinct in life (Fig. 12A) forming two curved lines running from snout towards orbits and along orbital margins ventrally. Subterminal oral disk with lips expanded vertically forming a dorsally oriented funnel (Fig. 11B); lateral corners of funnel distinct; upper lip notably smaller than lower; lips lack keratodonts, but bear short, low ridges, more densely arranged on upper than on lower labium, arranged in 18–24 (mean = 22) longitudinal rows and from 2–4 (mean = 3) transverse rows on upper labium to 4–6 (mean = 5) transverse rows on lower labium. Marginal papillae absent. Width of expanded funnel comprises over 75% of body length (Fig. 12A) and just 30% when folded in preservation (Fig. 12B).

In life tadpoles have dorsal side of body and upper flanks uniform brownish red or brownish orange (Fig. 12A, B). Lower flanks weakly mottled with dark brown, few round blackish spots on tail and dorsum; with orange neuromasts visible on dorsal surface (Fig. 12A). Abdomen light brownish orange, intestine not visible through body wall. Caudal muscles pale; tail fins translucent with a few darker spots (more on upper than on lower fin); dorsally tail with indistinct middorsal orange line (Fig. 12A). Eyes golden with black reticulations. Oral funnel pinkish orange with brownish red papillae (Fig. 12B). In preservative tadpole coloration gets much duller, but the general coloration pattern is still visible after 7 years in ethanol.

##### Advertisement call characteristics.

Refer to the Acoustic differentiation section, Table 4 and Fig. 5, for bioacoustic comparison of the new species with *O. synoria* and *O. gerti*. Refer to Appendix 3 for call variation data in *Ophryophryne elfina* sp. n.

##### Position in mtDNA phylogeny and sequence divergence.

The new species is reconstructed as a member of the *Ophryophryne* Group II (Fig. 2), forming a sister group with respect to the clade joining *O. gerti* and *O. synoria* (see Fig. 2). Uncorrected genetic *p*-distances between *Ophryophryne elfina* sp. n. partial sequences for the 16S rRNA gene and all homologous sequences available on GenBank included in the analysis (see Table 1) varied from 8.2% (with *O. gerti* *s. stricto*, clade A) to 10.0% (with *O. hansi* and *O. synoria*, clade C) (see Table 2). This degree of pairwise divergence in the 16S rRNA gene is greater than that usually representing differentiation at the species level in anura (Vences et al. 2005a, 2005b, Vieites et al. 2009, Poyarkov et al. 2015a, 2015b). Intraspecific variation in this gene fragment for *Ophryophryne elfina* sp. n. is significant — maximum sequence divergence between Nui Chua Mt. population (Fig. 2, clade D) and populations from the rest of the species range (Fig. 2, clade E) is *p* = 3.1%. Intraspecific variation in 16S rRNA gene fragment within one geographic population was lower, and ranged from 0.0% to 0.8% of substitutions.

**Figure 13.**
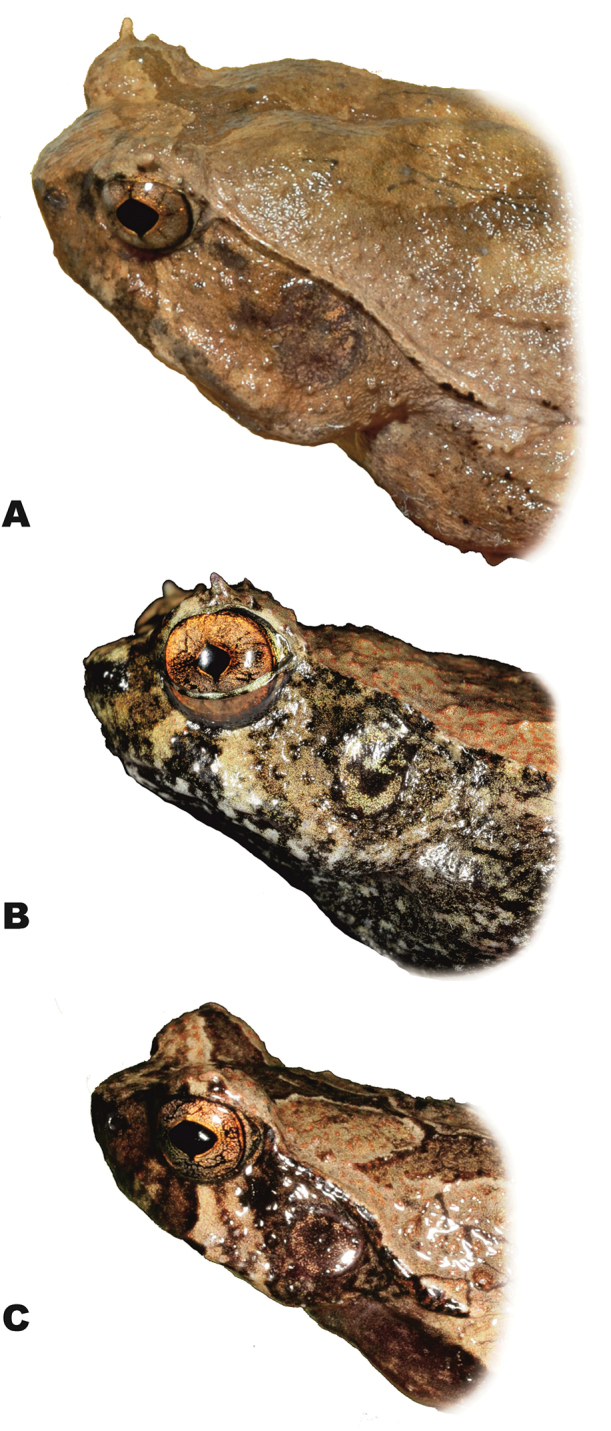
Comparison of the head coloration in life of three *Ophryophryne* species from the Langbian Plateau: **A** *Ophryophryne synoria*, Bu Gia Map N.P., Binh Phuoc Prov., Vietnam **B** *Ophryophryne gerti*, Chu Yang Sin N.P., Dak Lak Prov., Vietnam **C** *Ophryophryne elfina* sp. n., Hon Giao Mt., Bidoup–Nui Ba N.P., Lam Dong Prov. Photos by N.A. Poyarkov and N.L. Orlov.

##### Distribution.

*Ophryophryne elfina* sp. n. is found to be endemic to five provinces in (Lam Dong, Dak Lak, Khanh Hoa, Ninh Thuan and Phu Yen) in the northern and eastern part of the Langbian Plateau and its foothills in southern Vietnam (localities 6–12, Fig. 1). The new species is restricted to wet evergreen montane tropical and elfin forests, receiving high precipitation from the sea. Such wet forests are found only on high elevations in the central parts of the Langbian Plateau (e.g. 1900–2100 m a.s.l. on Bidoup Mt., Lam Dong Prov., Fig. 1, Loc. 6) or peripheral mountains remote from the sea (e.g. 1900–2300 m a.s.l. on Chu Pan Fan and Chu Yang Sin Mts., Dak Lak Prov., Fig. 1, Locs 10 and 11), but on the eastern foothills of the plateau which receive more precipitation, the new species is found at lower elevation (from 950 to 1510 m a.s.l. on Hon Ba Mt., Khanh Hoa Prov., Fig. 1, Loc. 8; 780 m a.s.l. on Nui Chua Mt., Ninh Thuan Prov., Fig. 1, Loc. 9; and 700 m in Phu Yen Prov., Fig. 1, Loc. 12).

##### Ecology.

All specimens were collected at night after heavy rains along montane cascade rocky streams, along small waterfalls, or intermittent rocky brooks; or found during the day time under tree-logs and within leaf litter in the limited fragments of primary montane wet polydominant evergreen tropical forests, with a high abundance of large rocks and fallen trees covered with a thick layer of mosses. This including high montane forests that are composed of the specific floral community known as “elfin” forests, with miniature trees (up to 10 m tall). These areas always have high precipitation and have much milder climate than other tropical forests in southern Vietnam: active breeding of the new species was recorded in February with temperatures of ca. 11–12°C.

On Bidoup Mt. summit (Lam Dong Prov.), *Ophryophryne elfina* sp. n. was recorded from 1890 to 2035 m a.s.l. in montane polydominant high canopy (trees up to 35 m tall) and elfin (trees up to 10 m tall) (sub)tropical forests with the predominance of trees of the family Fagaceae (*Lithocarpus* sp., *Castanopsis* sp.), Elaeocarpaceae (*Elaeocarpus* sp.), Lauraceae (*Machilus* sp.), Magnoliaceae (*Magnolia* sp., *Michelia* sp.), and occasional large trees of *Fokienia hodginsii* (Cupressaceae). These forests have thick leaf litter, numerous fallen logs and rocks covered with mosses, and an undergrowth that is predominated by ferns (mostly *Asplenium* sp., Aspleniaceae) (see Kuznetsov and Kuznetsova 2011) (Fig. 15A). On Hon Giao mountain ridge, the new species was found along mountain streams from 1800 to 1900 m a.s.l. in montane polydominant elfin forests with the predominance of trees of the family Fagaceae (*Castanopsis* sp., *Lithocarpus* sp.), Lauraceae (*Cinnamomum* sp., *Neolitsea* sp.), Ericaceae (*Rhododendron* sp.), Magnoliaceae (*Mangletia* sp., *Michelia* sp.), Elaeocarpaceae (*Elaeocarpus* sp.) and Podocarpaceae (*Podocarpus neriifolius*). This forest had a dense undergrowth of mosses, orchids (*Coelogyne* sp., *Dendrobium* sp.; Orchidaceae) and occasional ferns (*Cyathea* sp., Cyatheaceae) (see Kuznetsov and Kuznetsova 2011) (Fig. 15B). In Chu Yang Sin N.P. (Dak Lak Prov.), the new species was found from 1800 to 2100 m a.s.l. in montane forests with the predominance of trees of the families Pinaceae (*Pinus krempfii*; *Pinus kesiya*) and Fagaceae (*Lithocarpus* sp., *Castanopsis* sp.), with dense undergrowth of ferns, numerous rocks and fallen trees covered with mosses (Fig. 15C). In Hon Ba N.R. (Khanh Hoa Prov.), the new species was found from 950 to 1510 m a.s.l. along mountain streams in forests having polydominant composition including Fagaceae (*Lithocarpus* sp., *Quercus* sp.), Elaeocarpaceae (*Elaeocarpus* sp.), Theaceae (*Thea* sp., *Camellia* sp.), Lauraceae (*Cinnamomum* sp., *Neolitsea* sp.), Araliaceae (*Schefflera* sp.) and Rutaceae (*Euodia* sp.).

**Figure 14.**
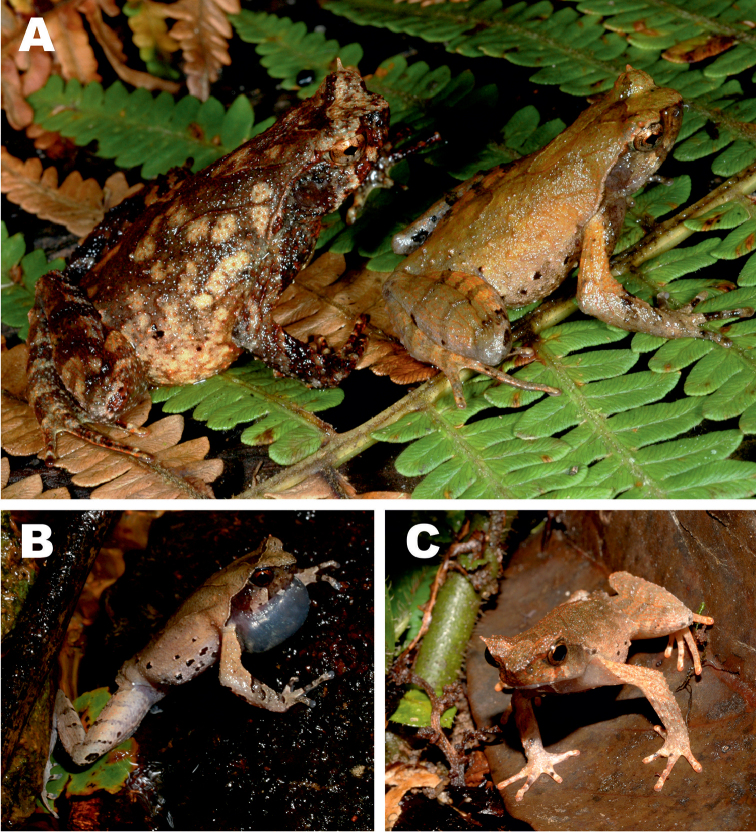
*Ophryophryne elfina* sp. n. in situ: **A** Two syntopically collected males of *Ophryophryne gerti* (left) and *Ophryophryne elfina* sp. n. (right) in Chu Yang Sin N.P., Dak Lak Prov., Vietnam, 1750 m a.s.l., photo by N.L. Orlov **B** calling adult male of *Ophryophryne elfina* sp. n. in Nui Chua Mt., Nui Chua N.P., Ninh Thuan Prov., Vietnam, 780 m a.s.l., photo by S.N. Nguyen **C** adult male of *Ophryophryne elfina* sp. n. in calling position in Hon Ba N.R., Khanh Hoa Prov., Vietnam, 1510 m a.s.l., photo by L.T. Nguyen.

On Bidoup Mt. summit (1890–2035 m a.s.l.; Lam Dong Prov.) *Ophryophryne elfina* sp. n. occurs in syntopy with *Leptobrachium pullum* (Smith, 1921), *Leptobrachium leucops* Stuart, Rowley, Tran, Le & Hoang, 2011, *Leptolalax bidoupensis* Stuart, Rowley, Tran, Le & Hoang, 2011, *Leptolalax pallidus* Rowley, Tran, Le, Dau, Peloso, Nguyen, Hoang, Nguyen & Ziegler, 2016, *Ingerophrynus galeatus* (Günther, 1864), *Hylarana montivaga* (Smith, 1921), *Rhacophorus vampyrus* Rowley, Le, Thi, Stuart & Hoang, 2010, *Theloderma palliatum* Rowley, Le, Hoang, Dau & Cao, 2011 and *Raorchestes gryllus* (Smith, 1924). On Hon Giao Mt. (1900–2000 m a.s.l.; borders of Lam Dong and Khanh Hoa provinces), *Ophryophryne elfina* sp. n. occurs in syntopy with *Leptobrachium leucops*, *Leptolalax bidoupensis*, *Duttaphrynus melanostictus* (Schneider, 1799), *Hylarana montivaga*, *Rhacophorus vampyrus* and *Raorchestes gryllus*. On Chu Pan Fan and Chu Yang Sin Mts. (1900 m a.s.l., Dak Lak Prov.), the new species is found in syntopy with Xenophrys cf. maosonensis (Bourret, 1937), *Leptobrachium* sp., *Leptolalax* sp., *Hylarana montivaga*, *Rhacophorus vampyrus* and *Raorchestes gryllus*. On Chu Yang Sin Mt. (1700–1800 m a.s.l., Dak Lak Prov.), the new species is also found in syntopy with *Ophryophryne gerti* (Fig. 14A). On Hon Ba Mt. (1500 m a.s.l., Khanh Hoa Prov.), the new species was recorded in syntopy with *Leptobrachium leucops*, *Leptolalax* sp., *Microhyla arboricola* Poyarkov, Vassilieva, Orlov, Galoyan, Tran, Le, Kretova & Geissler, 2014, *Theloderma truongsonense* (Orlov & Ho, 2005) and *Raorchestes gryllus*.

**Figure 15.**
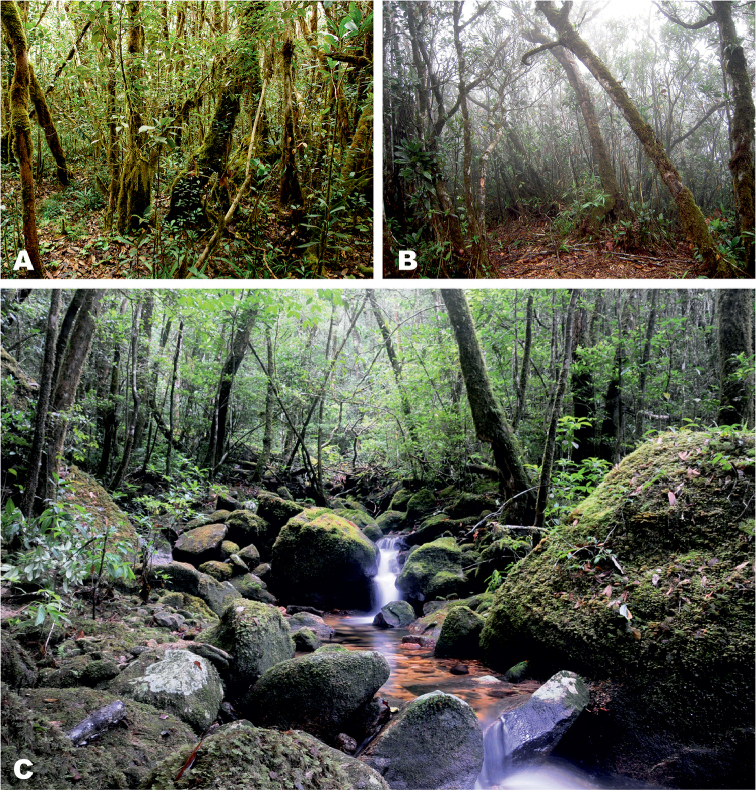
Natural habitat of *Ophryophryne elfina* sp. n. on Langbian Plateau, southern Vietnam. **A** Elfin forest on the top of Bidoup Mountain (ca. 2100 m a.s.l.), Bidoup–Nui Ba N.P., Lam Dong Prov. **B** elfin forest on the top of Hon Giao Mountain (ca. 2000 m a.s.l.), Bidoup–Nui Ba N.P., border of Lam Dong and Khanh Hoa provinces **C** typical breeding site along a mountain stream in an evergreen mixed subtropical montane forest on northern slopes of the Chu Pan Fan Mountain, Chu Yang Sin N.P., Dak Lak Prov. (type locality) (ca. 1900 m a.s.l.). Photos by N.A. Poyarkov and O.V. Morozova.

Reproductively active males were found while calling along streams, usually sitting on leaves of ferns or on the stone banks, rarely on rocks or large stones (see Fig. 14B, C). Some specimens were collected hiding amongst fern stems and were difficult to locate. Females were found hiding under tree logs or in the forest litter.

The ovaries of females contained well-developed unpigmented eggs with a diameter of approximately 2.2–2.8 mm (*N* = 15; measured from ZMMU ABV-00455, gravid female). On Hon Ba Mt., calling males were observed between 22 to 24 December, 22 to 28 March and 15 to 18 October. On Bidoup, Hon Giao and Chu Yang Sin Mts., reproductive activity and calling males were recorded from 10 February until mid-July. Tadpoles were found from April until July in the same streams where calling males were recorded; during the day time tadpoles hide under flat stones or dead leaves on the stream bed, but come out and can be visible in the shallow sandy parts of the stream at night. Metamorphosed individuals were observed in Chu Yang Sin N.P. in May.

##### Conservation status.

The full extent of the distribution of *Ophryophryne elfina* sp. n. is unknown, and the discovery of new localities on mountain ridges at elevations above 1500 m are highly anticipated. To date the species’ range includes the following nature conservation areas: Bidoup–Nui Ba N.P. (Lam Dong Prov.), Chu Yang Sin N.P. (Lam Dong Prov.), Hon Ba N.R. (Khanh Hoa Prov.) and Nui Chua N.P. (Ninh Thuan Prov.). However, population size and population dynamics of the new species are unknown. Given the available information, we suggest *Ophryophryne elfina* sp. n. to be considered as a Data Deficient species following IUCN’s Red List categories (IUCN Standards and Petitions Subcommittee 2016).

##### Comparisons.

*Ophryophryne elfina* sp. n. is one of the smallest species of its genus, with adult male size (SVL 26.9–33.9 mm) similar to that of *O. pachyproctus* (adult male SVL 28.0–30.0 mm).

*Ophryophryne elfina* sp. n. differs from allopatric *O. hansi* (central Vietnam and neighboring southeastern Laos and northeastern Cambodia; Ohler 2003, Stuart 2005, Stuart et al. 2006) by its much smaller adult size: *Ophryophryne elfina* sp. n. male SVL 26.9–33.9 mm, *N* = 29, female SVL 35.1–36.5 mm, *N* = 6 (vs. *O. hansi* male SVL 33.4–43.1 mm, *N* = 12, female SVL 45.1–53.9 mm, *N* = 5; our data), by skin of dorsal and lateral surfaces of head, body and limbs shagreened with numerous small tubercles (vs. notably tubercular and warty skin on dorsal surfaces), and short dorsolateral glandular ridge above each shoulder (vs. dorsolateral glandular ridges absent).

*Ophryophryne elfina* sp. n. differs from allopatric *O. pachyproctus* (Yunnan Prov. in southern China, central Vietnam and possibly adjacent regions of Laos; Bain et al. 2007, Fei et al. 2009, 2010, 2012) by lacking dermal protuberance with dermal flaps above cloacal opening (vs. present on *O. pachyproctus*), short dorsolateral glandular ridge above each shoulder not connected to posterior tips of “ >–< ”-shaped glandular parietoscapular-sacral ridge (vs. dorsolateral glandular ridge connected to posterior tips of “H”- or “Y”-shaped glandular parietoscapular-sacral ridge), supratympanic fold light brown dorsally on males (vs. white or light beige dorsally on males), and males with nuptial pad on first finger only (vs. one nuptial pad each on first and second fingers).

*Ophryophryne elfina* sp. n. differs from allopatric *O. microstoma* (Guangxi and Yunnan Provs., southern China to northern Vietnam, and northern Thailand; Khonsue and Thirakhupt 2001, Chan-ard 2003, Fei et al. 2009, 2010, 2012, Nguyen et al. 2009), by much smaller adult size: *Ophryophryne elfina* sp. n. male SVL 26.9–33.9 mm, N = 29, female SVL 35.1–36.5 mm, N = 6 (vs. *O. microstoma* male SVL 34.3–44.4 mm, N = 10, female SVL 39.4–57.0 mm, *N* = 7; Ohler 2003, Fei et al. 2009, Stuart et al. 2010; our data), short anterior dorsolateral glandular ridge above each shoulder (vs. dorsolateral ridges long, ca. 65–90% trunk length), and large tubercles posteriorly on dorsal surface of body (vs. large tubercles absent).

*Ophryophryne elfina* sp. n. differs from allopatric *O. poilani* (found in the mountains of the Tay Nguyen Plateau of central Vietnam and in adjacent areas of northeastern Cambodia, and, possibly, Laos; Bourret 1937, Stuart et al. 2010) by having smaller adult body size: *Ophryophryne elfina* sp. n. male SVL 26.9–33.9 mm, N = 29, female SVL 35.1–36.5 mm, N = 6 (vs. *O. poilani* male SVL 32.6–38.1 mm, N = 14, female SVL 47.4–50.8 mm, *N* = 2; Stuart et al. 2010), short dorsolateral glandular ridge above the shoulder not connected to posterior tips of “ >–< ”-shaped parietoscapular-sacral glandular ridge (vs. dorsolateral glandular ridge connected to posterior tips of “ >–< ”-shaped parietoscapular-sacral glandular ridge), and lacking characteristic dark “mask-like” coloration of temporal region, and supratympanic fold being dark brown ventrally and light brown dorsally on males (vs. temporal region and lateral surfaces of head uniformly dark-colored forming a dark “mask”, extending posteriorly towards axilla; supratympanic fold edged with white on males).

*Ophryophryne elfina* sp. n. differs from sympatric *O. synoria* (found at lower elevation from 200 to 1500 m a.s.l. in the foothills of the Langbian Plateau in southern Vietnam and adjacent easternmost hilly Cambodia) by much smaller adults body size: *Ophryophryne elfina* sp. n. male SVL 26.9–33.9 mm, N = 29, female SVL 35.1–36.5 mm, N = 6 (vs. *O. synoria* male SVL 38.2–53.7 mm, N = 14, female SVL 51.4–70.7 mm, N = 3; our data; Fig. 3), red-orange nuptial pad (in life) on first finger only (vs. two nuptial pads, covered in brown microgranules, large on first finger, covering entire dorsal metacarpal extending to 3/4 basal phalange length, on second finger medium sized on metacarpal extending to mid basal phalange on inner dorsal side), numerous bright red-orange asperities (in life) on dorsal and lateral surfaces of body, head and dorsal surfaces of limbs (vs. black and white asperities, small sized, spinular, moderately dense in narrow band along lower jaw, and on posterior upper jaw, few on tympanic region [exlcuding tympanum], along supratemporal folds and on posterior upper eyelids; some on anterior dorsum, becoming moderately dense posteriorly, above and surrounding cloaca, few on dorsal shanks, and absent on remaining surfaces on holotype of *O. synoria*), and smaller tympanum/eye diameter ratio, TYD/ED 48.9%–62.6%, N=29 (vs. TYD/ED 64.8%–85.2%, N=14).

*Ophryophryne elfina* sp. n. differs from sympatric *O. gerti* (found at mid-elevations from 700 to 2000 m a.s.l. in the central and northern parts of the Langbian Plateau in southern Vietnam) by typically smaller adults body size: *Ophryophryne elfina* sp. n. male SVL 26.9–33.9 mm, N = 29, female SVL 35.1–36.5 mm, N = 6 (vs. *O. gerti* male SVL 31.7–42.2 mm, N = 15, female SVL 43.1–47.4 mm, *N* = 3; our data; Fig. 3), bright red-orange nuptial pads on males in life (vs. grey or black-brown nuptial pads on males in life), short dorsolateral glandular ridge above each shoulder, not connected to posterior tips of “ >–< ”-shaped parietoscapular-sacral glandular ridge, see Figs 6G–H, and 8 (vs. strong dorsolateral glandular ridge from above each shoulder to approximately 4/5 distance between axilla and groin, connecting with posterior tips of “ >–< ”-shaped parietoscapular-sacral glandular ridge; see Figs 6A–C, 7), skin on dorsal and lateral surfaces of body shagreened with numerous small tubercles (vs. skin on dorsum and sides of body granular, with numerous small and medium-sized tubercles and larger warts, see Fig. 6A–C), dark hourglass-shaped markings on dorsum normally edged with white, see Figs 6H and 8 (vs. dark hourglass-shape on dorsum indistinct or, if present, unclear and not edged with white, Fig. 6A–C), and throat, chest and abdomen having generally lighter coloration than in *O. gerti*.

Though available information on tadpole morphology of *Oprhyophryne* is very limited (Liu and Hu 1962, Huang et al. 1991, Grosjean 2003, Fei et al. 2009), the tadpoles assigned to the new species based on the analysis of short 16S rRNA gene sequences (Table 1) have certain morphological characteristics that could be useful for distinguishing the larval stage of *Ophryophryne elfina* sp. n. from other *Ophryophryne* species. From tadpoles of *O. microstoma*, described in detail by Grosjean (2003), tadpoles of *Ophryophryne elfina* sp. n. differ mainly by possessing a longer tail: TOL/BL ratio 231.3 ± 11%, *N* = 5 (vs. TOL/BL < 210%, *N* = 52 on *O. microstoma*), and tail tip rounded (vs. tail tip bluntly pointed), mean = 22 longitudinal rows of papillae and from 2–4 (mean = 3) transverse rows of papillae on the upper labium and 4–6 (mean = 5) transverse rows of papillae on the lower labium, *N* = 5 (vs. mean = 20 longitudinal rows of papillae, and two upper labium and four lower labium transverse rows of papillae [without clear limits], *N* = 52); however, some of these differences may relate to the fact that Grosjeans’ description was based on later developmental stages (Gosner stage 37) than our sampling (Gosners’ stage 25).

DNA-barcoding using short sequences for 16S rRNA (Table 1) also enabled us to identify tadpoles of sympatric *Ophryophryne* species from the Langbian Plateau, and though our sampling is not big enough to provide detailed morphological descriptions of larval morphology for *O. gerti* and *O. synoria*, we found some differences in coloration of tadpoles which may be useful for preliminary diagnostics of the three sympatric *Ophryophryne* species in the wild. Despite overall morphological similarity, both *O. gerti* and *O. synoria* show the presence of light golden to copper blotches on dorsal surfaces of the body and tail, whereas *Ophryophryne elfina* sp. n. tadpoles always have distinctive uniform brownish coloration with small coppery dots (Fig. 12A–B).

Despite overall similarity, advertisement calls of each *Ophryophryne* species inhabiting the Langbian Plateau are easily diagnosable based on acoustic parameters. Some parameter values clearly differ between all of the studied species (Fig. 5). For example, the highest values of the repetition rate per recording and per series are found for *O. synoria* (3.07 ± 0.13 calls/s, *N* = 3, and 5.34 ± 0.15 calls/s, *N* = 15, respectively) while the lowest are reported for *O. gerti* (0.35 ± 0.14 calls/s, *N* = 3, and 2.33 ± 0.03 calls/s, *N* = 108, respectively). *Ophryophryne elfina* sp. n. occupies an intermediate position between these two species (1.18 ± 0.2 calls/s, *N* = 5, and 3.87 ± 0.07 calls/s, *N* = 140, respectively; differences significant, F_2.8_ = 46.7, p < 0.001 and F_2.260_ = 220.7, p < 0.001, respectively, one-way ANOVA) .

The call temporal parameters for *Ophryophryne elfina* sp. n. compared to sympatric *O. synoria* and *O. gerti*, also differ for series duration, which is the highest in calls of *Ophryophryne elfina* sp. n., comprising 3.42 ± 0.11 s, *N* = 140 (see Table 4) (differences with *O. gerti* significant, F_2.267_ = 40.4, p < 0.001, one-way ANOVA; differences with *O. synoria* not significant, see Table 4 for details). The advertisement call of the new species is further significantly different from calls of *O. gerti* for values of a number of acoustic parameters (see Table 4), such as the number of calls per series (12.84 ± 0.41, *N* = 140, versus 4.64 ± 0.16, N = 115, for *O. gerti*; differences significant, F_2.267_ = 151.4, p < 0.001, one-way ANOVA), the call duration (73 ± 0.23 ms, *N* = 1797, versus 104 ± 0.5 ms, *N* = 533, for *O. gerti*; differences significant, H_2.2530_ = 1345.1, p < 0.001, Kruskal-Wallis ANOVA) and inter-calls interval (207 ± 2.06 ms, *N* = 1657, versus 421.54 ± 4.17 ms, *N* = 418, for *O. gerti*; differences significant, H_2.2260_ = 1008.5, p < 0.001, Kruskal-Wallis ANOVA). The advertisement call of *Ophryophryne elfina* sp. n. is further different from calls of *O. synoria* in the frequency of maximum amplitude (4645.94 ± 4.39 Hz, *N* = 1797, versus 3798.9 ± 4.87 Hz, *N* = 200; differences significant, H_2.2530_ = 1030.2, p < 0.001 (U = 0), Kruskal-Wallis ANOVA).

Finally, the new species is markedly distinct from all other congeners for which comparable sequences are available, including it closest relatives *O. gerti* and *O. synoria*, by relatively large genetic distances in 16S rRNA mtDNA gene fragment (*p* ≥ 8.2%).

## Discussion

The data presented here provide the most extensive molecular sampling for the elucidation of phylogentic relationships within the genus *Ophryophryne*. According to our data, genetic variation within *Ophryophryne* appears to be strongly geographically structured. Thus, our results indicate the division of the genus *Ophryophryne* into two major reciprocally monophyletic groups: one corresponding to species found on the Langbian Plateau (Group II, Fig. 2), and another joining species found outside the plateau from central and northern Truong Son and adjacent areas (Group I, Fig. 2). Our data support the hypothesis that eastern Indochina, including the central and southern parts of the Truong Son Mountains (known also as Tay Nguyen Plateau), host the highest diversity of *Ophryophryne*, and was the center of radiation for this genus (Orlov and Ananjeva 2007, Mahony et al. 2017). Similar patterns of geographic structuring of mtDNA lineages were reported for the genus *Leptolalax*, another megophryid genus inhabiting the Truong Son Mountains (Poyarkov et al. 2015a, Rowley et al. 2016).

A hidden diversity of *Ophryophryne* frogs is revealed in the mountains of the Langbian Plateau, where previously only one species, *O. gerti*, was correctly reported (Ohler 2003, Nguyen et al. 2009, Stuart et al. 2010). In our study it is shown that the previous records of O. cf. gerti from central Vietnam and Laos (Ohler 2003, Bain et al. 2007) actually belong to different species of *Ophryophryne* and thus we clarify the range of *O. gerti* showing that this species is likely endemic to the Langbian Plateau. The known distribution of *O. synoria* is also extended, previously known exclusively from Cambodia (Stuart et al. 2006) and adjacent provinces of Vietnam (Vassilieva et al. 2016), and demonstrate that this species has a considerably wider range encompassing the central, northern and western edges of the Langbian Plateau. Finally, we describe the new species *Ophryophrye elfina* sp. n., which is endemic to the northern and eastern edges of the plateau. Thus, the Langbian Plateau was a center of *Ophryophryne* radiation and cradles three endemic species of these frogs; all of them are sympatric in eastern and northern parts of the plateau and often can be recorded in synbiotopy.

*Ophryophrye elfina* sp. n. represents one of the smallest known species of the genus *Ophryophryne*. We found that the three *Ophryophryne* species of the Langbian Plateau are differentiated in body size with the largest species *O. synoria* preferring lowland and foothill monsoon forests at elevations from 200 to 1500 m a.s.l., medium-sized *O. gerti* found in evergreen montane tropical forests at mid-elevations from 700 to 2000 m a.s.l. and the smallest species *Ophryophryne elfina* sp. n. being restricted to wet montane subtropical forests at elevations from 700 to 2100 m a.s.l., including elfin forests at high elevations. It is probably not surprising that advertisement calls of the three occasionally sympatric *Ophryophryne* species show significant differences both in call structure and frequency parameters (see Table 4, Fig. 5), and the three studied species are characterized by relatively high values of the frequency parameters (as compared to several other Megophryidae species studied acoustically, especially of the genera *Leptolalax*, see review in Rowley et al. 2016, and *Leptobrachium*, see e.g. Stuart et al. 2010). The high frequency parameters may be related with their tendency to vocalize in close proximity to mountain cascade streams, which would create a low-frequency background noise (Preininger et al. 2007). It was shown that low background noise may induce frogs to call at higher frequency rates than expected from their body size, thereby improving the signal-to-noise ratio of their calls (Penna et al. 2005, Wells 2007, Goutte et al. 2016). Our study also recorded that the values of some temporal call parameters of *Ophryophryne elfina* sp. n. significantly differ between February (average temperature 11.3°C) and April recordings (average temperature 17.5°C; see Appendix 3 for details). Our results correspond with previous reports that intraspecific variation of temporal parameters of anuran calls can depend upon temperature (e.g., Gerhardt and Huber 2002).

The frequency of maximum amplitude coincides with the fundamental frequency for all *Ophryophryne* species, and have almost equal values for *Ophryophryne elfina* sp. n. and *O. gerti* (4645.94 ± 4.39 Hz, *N* = 1797, and 4845.99 ± 4.22 Hz, *N* = 533, respectively). The frequency of maximum amplitude of *O. synoria* is significantly lower (3798.9 ± 4.87 Hz, *N* = 200; see Table 4 for details), which may be related to the larger body size of the latter species (Stuart et al. 2006, Wells 2007). Further studies on acoustic communication of Langbian *Ophryophryne* species in areas of allopatry and sympatry would be valuable for better understanding the bioacoustic patterns observed here.

The Langbian Plateau is known for its high herpetofaunal diversity and endemism, a significant portion of which has been discovered only recently (e.g., Orlov et al. 2008, 2012, Rowley et al. 2010c, 2011a, 2011b, 2016, Stuart et al. 2011, Poyarkov [Paiarkov] and Vassilieva 2011, Nazarov et al. 2012, Chan et al. 2013, Hartmann et al. 2013, Geissler et al. 2014, 2015, Vassilieva et al. 2014, Poyarkov et al. 2014, 2015a, 2015b). Despite this increase in species discoveries, many areas of the Annamites have received little scientific attention and are very likely to host further previously unknown diversity. The need for biological exploration in this region is made more urgent given the ongoing loss of natural habitats due to logging, road construction, increasing agricultural pressure and other human activities (Meijer 1973, De Koninck 1999, Laurance 2007, Meyfroidt and Lambin 2008, Kuznetsov and Kuznetsova 2011).

Habitat loss is the greatest threat to amphibians in southeast Asia, and the amphibians of the region appear to be particularly vulnerable to habitat alterations (Rowley et al. 2010b). Frogs of the genus *Ophryophryne* depend on fast-flowing clean mountain streams for reproduction, and appear to be restricted to relatively undisturbed broadleaf evergreen forests: such habitat specialist range-restricted species are likely to be most at risk (Poyarkov et al. 2012, Rowley et al. 2010b, 2016). Deforestation, habitat loss and modification are continued threats in southern Indochina (Meyfroidt and Lambin 2008), and further studies on herpetofaunal biodiversity in this region are urgently required for elaboration of effective conservation measures.

### Addendum (added post manuscript acceptance)

Due to the simultaneous review period of the present paper, and the now recently published Mahony et al. (2017), we chose to preliminarily use *Ophryophryne* at the genus level (following Chen et al. 2017; published online 1 December 2016), pending the publication of the taxonomic justification by Mahony et al. (2017) which supports a subgenus level classification of *Ophryophryne* within *Megophrys*. Mahony et al. (2017) also provided the replacement name Megophrys (Ophryophryne) koui Mahony, Foley, Biju & Teeling, 2017 for *Ophryophryne pachyproctus* Kou, 1985. We suggest that the new species combination *Ophryophryne elfina* sp. n. should hereafter be referred to as Megophrys (Ophryophryne) elfina (Poyarkov, Duong, Orlov, Gogoleva, Vassilieva, Nguyen, Nguyen, Nguyen, Che & Mahony) to reflect this revised taxonomy.

## Author contributions

NA Poyarkov envisioned the original idea of the manuscript, collected material and data in the field and in the lab, executed this study and wrote the manuscript; TV Duong performed morphometric, molecular and phylogenetic analyses; NL Orlov collected material in the field; SS Gogoleva collected data in the field and performed acoustic analyses and wrote the relevant parts of the manuscript; AB Vassilieva collected material and data in the field; LT Nguyen collected material in the field and assisted with morphological analysis; VDH Nguyen, J Che and SN Nguyen collected material in the field and provided additional molecular data; S Mahony examined type and comparative specimens, performed molecular analysis, provided redescription of types, and edited the manuscript. All authors contributed to this paper sufficiently.

## Supplementary Material

XML Treatment for Ophryophryne gerti

XML Treatment for Ophryophryne synoria

XML Treatment for Ophryophryne elfina

## Appendix 1

Examined material, museum IDs given in bold.

*Ophryophryne elfina* sp. n.: **ZMMU A-5669** (Vietnam, Dak Lak Prov., Chu Yang Sin N.P.; NAP-02658; 1 adult male; holotype); **ZMMU A-5170** (Vietnam, Lam Dong Prov., Bidoup–Nui Ba N.P., Bidoup Mt.; ABV-00455; ABV-00454; ABV-00472; ABV-00471; 4 adults; paratypes); **ZMMU A-4788** (Vietnam, Lam Dong Prov., Bidoup–Nui Ba N.P., Hon Giao Mt., Bidoup Mt.; NAP-01455; NAP-01449; NAP-01450; NAP-01460; 4 adults; paratypes); **ZMMU A-5674** (Vietnam, Lam Dong Prov., Bidoup–Nui Ba N.P., Hon Giao Mt.; NAP-01451; NAP-01452; 2 adults; paratypes); **ZMMU A-5675** (Vietnam, Lam Dong Prov., Bidoup–Nui Ba N.P., Bidoup Mt.; NAP-01456; NAP-01459; 2 adults; paratypes); **ZMMU A-5691** (Vietnam, Dak Lak Prov., Chu Yang Sin N.P.; ABV-00580, ABV-00581; 2 juveniles; paratypes); **ZMMU A-5650** (Vietnam, Dak Lak Prov., Chu Yang Sin N.P.; 10 adults); **ZISP 12836**–**12879** (Vietnam, Dak Lak Prov., Chu Yang Sin N.P.; 44 adults; tentative identification); **ZMMU A-5665** (Vietnam, Dak Lak, Chu Yang Sin N.P.; ROM 36348; ROM 36289; ROM 36554; ROM 36470; 4 adults); **ZISP 12880**–**12884** (Vietnam, Dak Lak Prov., Chu Yang Sin N.P.; ROM 36471; ROM 36472; ROM 36469; ROM 36366; ROM 36349; 5 adults); **ZMMU A-3937** (Vietnam, Khanh Hoa Prov., Hon Ba N.R.; 1 adult); **ZMMU A-4935** (Vietnam, Khanh Hoa Prov., Hon Ba N.R.; ABV-00316; 1 adult); **ZMMU A-4716** (Vietnam, Lam Dong Prov., Bidoup–Nui Ba N.P., Hon Giao Mt., Bidoup Mt.; NAP-01871; NAP-01782; NAP-01758; NAP-01757; NAP-01783; 5 adults); **ITBCZ 2786, 2788, 2792, 2828, 2908**–**09, 2918**–**19, 3502** (Vietnam, Khanh Hoa Prov., Hon Ba N.R.; 9 adults); **ZMMU A-5679** (Vietnam, Lam Dong Prov., Bidoup–Nui Ba N.P., Bidoup Mt.; NAP-01169; 7 larvae); **ZMMU A-5684** (Vietnam, Dak Lak Prov., Chu Yang Sin N.P., Chu Pan Fan Mt.; NAP-02673; 4 larvae).

*Ophryophryne gerti*: **BMNH 1921.4.1.324** (Vietnam, Lam Dong Prov., sout-east of Da Lat, Cam Ly river; 1 adult male; holotype); **BMNH 1921.4.1.323** (Vietnam, Lam Dong Prov., Dran; 1 immature female; paratype); **ZMMU A-4714** (Vietnam, Lam Dong Prov., Bidoup–Nui Ba N.P., Giang Ly; NAP-01878; 1 adult); **ZMMU A-4715** (Vietnam, Lam Dong Prov., Bidoup–Nui Ba N.P., Giang Ly; NAP-01790; NAP-01788; 2 adults); **ZMMU A-4718** (Vietnam, Lam Dong Prov., Bidoup–Nui Ba N.P., Giang Ly; NAP-01789; 1 adult); **ZMMU A-4843** (Vietnam, Dak Lak Prov., Chu Yang Sin N.P.; ABV-00067; 1 adult); **ZMMU A-5670** (Vietnam, Dak Lak Prov., Chu Yang Sin N.P.; NAP-02758; NAP-02759; NAP-02760; NAP-02761; 4 adults); **ZMMU A-5673** (Vietnam, Dak Lak Prov., Chu Yang Sin N.P.; ABV-00530; ABV-00577; 2 adults); **ZISP 12740–12747** (Vietnam, Dak Lak Prov., Chu Yang Sin N.P.; ROM 36522–36529; 8 adults).

*Ophryophryne hansi*: **FMNH 252879, FMNH 252880, FMNH 252882, FMNH 252884** (Vietnam, Gia-Lai Prov., Ankhe Dist., 60 km to the northwest from Kannack; 1 adult male; holotype; 2 adult males and 1 adult female; paratypes); **FMNH 252873** (Vietnam, Gia-Lai Prov., Ankhe Dist., 20 km to the northwest from Kannack; 1 adult male); **AMNH 163680** (Vietnam, Quang Nam Prov., Tra My Dist., Tra Don Commune; 1 adult male); **AMNH 169284** (Vietnam, Thua Tien Hue Prov., A Luoi Dist., Tram Tra Ve (Forestry station of Huong Giang State Forestry Enterprise); 1 adult male); **AMNH 169286** (Vietnam, Thua Tien Hue Prov., Huong Thuy Dist., Khe Dau Station; 1 adult male); **AMNH 161353** (Vietnam, Ha Tinh Prov., Huong Son, Huong Son Reserve; 1 adult male); **FMNH 258008–258009, FMNH 258046–258051** (Lao P.D.R., Xe Kong Prov., Kaleum Dist., Xe Sap National Biodiversity Conservation Area; 5 adult males and 4 adult females).

*Ophryophryne miscrostoma*: **BMNH 1947.2.22.50, BMNH 1947.2.22.52** (Vietnam, Mau Son Mts.; 2 adult females; lectotype & paralectotype); **AMNH 168682** (Vietnam, Lao Cai Prov., Van Ban Dist., Nam Tha Commune; 1 adult male).

*Ophryophryne pachyproctus*: **YU A8311032** (China, Yunnan Prov., Xishuangbanna Pref., Mengla Co., Yiwu, Zhoushihe river; 1 adult male; holotype); **YU A8311033–A8311037, YU A845099–A845100** (China, Yunnan Prov., Xishuangbanna Pref., Mengla Co., Yiwu, Zhoushihe river; 8 adult males; paratypes).

Ophryophryne cf. poilani: **AMNH 169287** (Vietnam, Thua Tien Hue Prov., A Luoi Dist., A Pat Forestry Protection Department Range Station; 1 adult female); **AMNH 163668** (Vietnam, Quang Nam Prov., Tra My Dist., Tra Don Commune; 1 adult female).

*Ophryophryne synoria*: **FMNH 262779** (Cambodia, Mondolkiri Prov., O’Rang Dist., O Chung Chry stream; 1 adult male; holotype); **FMNH 262778** (Cambodia, Mondolkiri Prov., O’Rang Dist., O Chung Chry stream; 1 adult male; paratype); **ZMMU A-5003** (Vietnam, Binh Phuoc Prov., Bu Gia Map N.P.; ABV-00379; ABV-00380; ABV-00376; ABV-00381; ABV-00377; 5 adults); **ZMMU A-4516** (Vietnam, Binh Phuoc Prov., Bu Gia Map N.P.; NAP-00729; NAP-00727; NAP-00728; NAP-00730; NAP-00731; 5 adults); **ZMMU A-4864** (Vietnam, Lam Dong Prov., Loc Bac forestry; ABV-00159; ABV-00209; 2 adults); **ZMMU A-5676** (Vietnam, Lam Dong Prov., Bidoup–Nui Ba N.P., Giang Ly; NAP-01438; NAP-01437; 2 adults).

*Ophryophryne* sp.: **BMNH 1972.1524** (Lao P.D.R., “Pak Maat”; 1 adult male; paratype of *O. gerti*).

## Appendix 2

**Table:** Factor coordinates of the morphometric characters used in PCA analysis, based on correlations (factors 1 to 3).

Character	factor 1	factor 2	factor 3
**SVL**	-0.969013	-0.052119	-0.061729
**HW**	-0.972214	-0.094198	-0.099264
**HL**	-0.963376	-0.112316	-0.089300
**ED**	-0.872338	-0.060587	0.293135
**TYD**	-0.853903	-0.302433	0.054377
**TYE**	-0.869868	-0.053510	-0.256344
**SL**	-0.817708	0.454058	-0.126722
**EN**	-0.774425	-0.018511	-0.529049
**NS**	-0.302259	0.915825	0.017344
**IUE**	-0.866068	-0.171158	0.204049
**IN**	-0.818811	0.248915	0.129748
**UEW**	-0.848819	0.181381	0.241783
**FAL**	-0.937173	-0.162120	-0.073309
**HAL**	-0.957549	0.054374	-0.001476
**FIL**	-0.931528	-0.047484	0.090017
**FIIL**	-0.939026	-0.049085	0.087852
**FIIIL**	-0.963650	0.030269	-0.041650
**FIVL**	-0.902097	0.102388	-0.091818
**SHL**	-0.950468	-0.104557	0.046962
**TL**	-0.921529	-0.091021	0.019328
**FOL**	-0.918076	0.080433	0.100931
**TFOL**	-0.971401	0.003596	0.002474
**IMT**	-0.761261	-0.079858	0.068715

## Appendix 3

**Table:** Measurements of advertisement call temporal parameters and one-way ANOVA/Kruskal-Wallis results for comparison (*p < 0.001) between April and February sets of call recordings for *Ophryophryne elfina* sp. n. Seconds (s), milliseconds (ms).

Parameters	*O. elfina* sp. n. 10–15 April 2014	*O. elfina* sp. n. 10 February 2015	Tukey/ Mann-Whitney U post hoc tests	ANOVA/ Kruskal-Wallis results
Temperature of recording	11.3–11.4°C	17.0–17.5°C	–	–
Number of males	2	1	–	–
Number of recordings	3	2	–	–
Number of series	93	47	–	–
Number of calls	1301	496	–	–
Call repetition rate per recording (calls/s)	1.25 ± 0.35 (0.77–1.95) *N* = 3	1.07 ± 0.06 (1.01–1.12) *N* = 2	p = 0.71	F_1.3_ = 0.2
Number of calls per series	14 ± 0.45 (2–22) *N* = 93	10.53 ± 0.72 (3–21) *N* = 47	p < 0.001	F_1.138_ = 18.2*
Series duration (s)	3.37 ± 0.1 (0.43– 5.05) *N* = 93	3.52 ± 0.27 (0.61–9) *N* = 47	p = 0.52	F_1.138_ = 0.4
Call repetition rate per series (calls/s)	4.22 ± 0.08 (2.92–5.49) *N* = 93	3.18 ± 0.1 (1.33–4.91) *N* = 47	p < 0.001	F_1.138_ = 63.9*
Call duration (ms)	70 ± 0.27 (25–112) *N* = 1301	79 ± 0.32 (48–102) *N* = 496	p < 0.001 (U = 131097)	H_1.1798_ = 380.2*
Inter-calls interval (ms)	184 ± 1.72 (96–621) *N* = 1209	271.17 ± 4.92 (102–942) *N* = 448	p < 0.001 (U = 109162)	H_1.1657_ = 349.2*
Inter-series interval (s)	6.72 ± 0.54 (1.26–31.65) *N* = 90	6.09 ± 0.56 (1.42–22.14) *N* = 45	p = 0.83 (U = 2022)	H_1.136_ = 0.05
